# Diacylglycerol kinase *kappa* is a target of the Prader–Willi syndrome gene *SNORD116*

**DOI:** 10.26508/lsa.202603723

**Published:** 2026-11-04

**Authors:** Yiying Zhang, Kaiying Guo, Gabriel F Batzli, Haoquan Zhao, Lisa C Burnett, Charles A LeDuc, George Stratigopoulos, Daniel G Carolan, Claudia A Doege, Yufeng Shen, Rudolph L Leibel

**Affiliations:** 1 Department of Pediatrics, Division of Molecular Genetics, Columbia University Irving Medical Center , New York, NY, USA; 2 Department of Systems Biology, Columbia University Irving Medical Center , New York, NY, USA; 3 Foundation for Prader-Willi Research, Covina, CA, USA; 4 Department of Pediatrics, Division of Genetics and Genomics, Boston Children’s Hospital and Harvard Medical School, Boston, MA, USA; 5 Department of Neuroscience, University of Minnesota, Minneapolis, MN, USA; 6 Brain Sciences Center, Minneapolis VA Health Care System, Minneapolis, MN, USA; 7 Department of Pathology and Cell Biology, Naomi Berrie Diabetes Center, Columbia University Irving Medical Center , New York, NY, USA

## Abstract

*SNORD116* is a small nucleolar RNA deficient in Prader–Willi syndrome individuals. The mechanisms by which *SNORD116* conveys its effects are unknown. Here, transcriptomic analysis showed that diacylglycerol kinase *kappa* (*Dgkk*) expression was elevated in the hypothalamus of *Snord116*-deficient mice. *In silico* analyses predicted a strong RNA:RNA interaction between *Snord116* and *Dgkk* mRNA that is fully conserved in mouse and human. In vitro experiments in human cellular models demonstrated that *SNORD116* mediates 2′-O-methylation of *DGKK* mRNA and regulates *DGKK* mRNA and protein expression. In *Snord116*-deficient mouse neurons, increased *Dgkk* expression was associated with attenuated diacylglycerol-mediated activation of ERK and PKC, critical elements in neuronal signaling cascades. In mice, brain *Dgkk* expression peaks during late embryonic and early postnatal periods, coinciding with peak rates of neurogenesis; its expression in adults is restricted mainly to brain regions implicated in Prader–Willi syndrome pathophysiology, including the arcuate nucleus of the hypothalamus. Fasting acutely decreased hypothalamic *Dgkk* expression in wild-type mice. Increased *Dgkk* expression in *Snord116*-deficient mice could reflect derangements in this pathway that affects both brain development and cell-specific functions.

## Abstract (precis)

This study examines the impacts of *SNORD116* on the expression and RNA modifications of diacylglycerol kinase *kappa*, an enzyme implicated in neurogenesis and synaptic function that are likely relevant to aspects of the pathophysiology of Prader–Willi syndrome.

## Introduction

**Figure 1.**
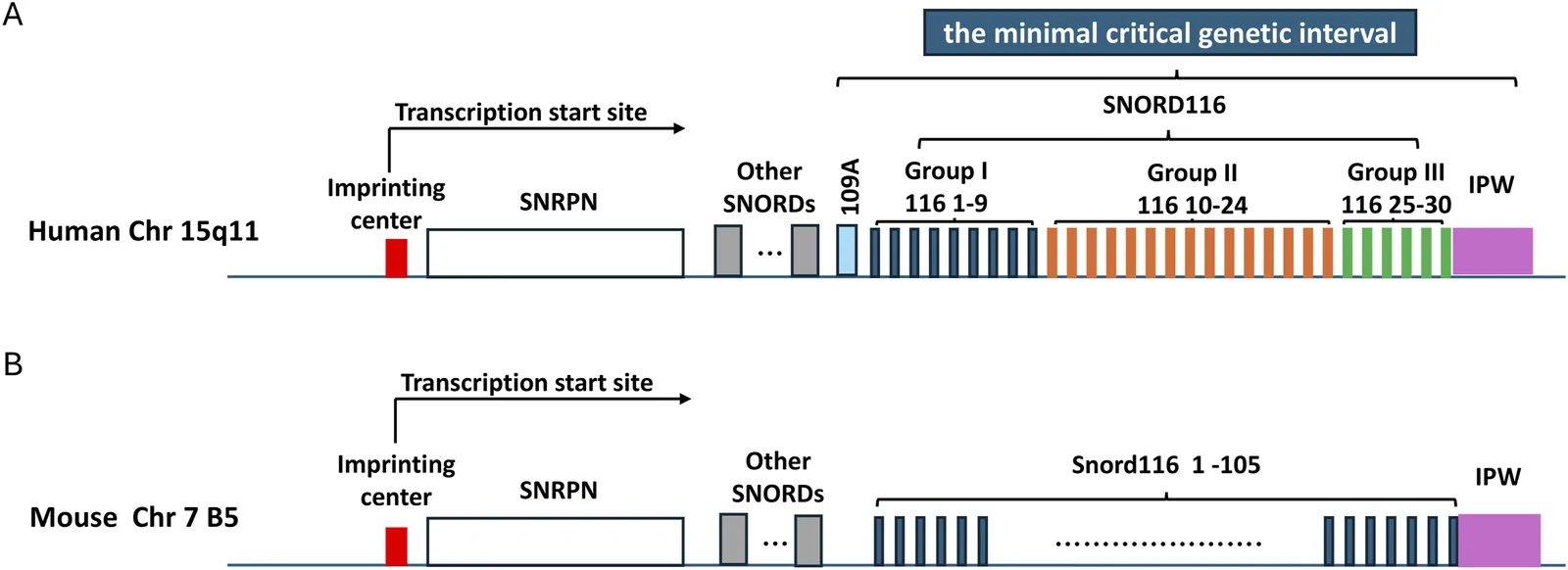
Human *SNORD116* subfamilies and their positions relative to the transcription start site and the *SNRPN* transcript and the mouse syntenic region. **(A)** Minimal critical genetic interval (MCR) of PWS on human Chr15q11. Groups 1-III of human *SNORD116* in the MCR are shown. Group 1 includes *SNORD116* 1–9, group II includes *SNORD116* 10–24, and group III includes *SNORD116* 25–30 (Good & Kocher, 2017). Baldini et al reported a slightly different classification of human *SNORD116* elements (Baldini et al, 2022). The position of human *SNORD116* elements relative to the transcription start site and the *SNRPN* transcript are indicated (Runte et al, 2001). **(B)** Mouse syntenic region of PWS is on Chr 7B5. All mouse *Snord116* elements are virtually identical to each other and share about 78% sequence homology with group I human *SNORD116* (Good & Kocher, 2017). Human PWS MCR also contains the *SNORD109A* locus (pale blue box) that is absent in the mouse syntenic region.

Prader–Willi syndrome (PWS) is a complex genetic disorder caused by the loss of expression of a group of paternally expressed genes in the imprinted 15q11-q13 chromosomal region (Cassidy et al, 2012; Butler et al, 2019). PWS is characterized by multiple metabolic, neurodevelopmental, and behavioral impairments, including failure to thrive during infancy, growth retardation, hypogonadism, sleep disorder, and in many cases severe hyperphagic obesity and autism spectrum disorder (Cassidy et al, 2012; Butler et al, 2019). The molecular mechanisms underlying the pathogenesis of PWS remain unknown. The minimal critical genetic interval of PWS, which is deleted or silenced in all PWS individuals, encodes only three noncoding RNAs, including the box C/D small nucleolar RNA (snoRNA) *SNORD116* cluster (Fig 1A). The available mouse models of *Snord116* deficiency, *Snord116* knockout (*Snord116* KO), and the paternal Imprinting Center deletion (IC del), which results in the loss of all paternally expressed genes, including *Snord116*, do not recapitulate all clinical features of PWS. However, they do share salient PWS traits in humans, including impaired somatic growth, abnormal neuroendocrine function, hyperghrelinemia, and motor learning deficits (Yang et al, 1998; Ding et al, 2008; Cassidy et al, 2012; Adhikari et al, 2019). The molecular functions and mechanisms of action of snoRNAs generally and *SNORD116* specifically remain poorly understood, but molecular processes attributed to specific SNORDs include ribosomal RNA processing, RNA methylation, and splicing regulation (Deogharia & Majumder, 2018; Monaco et al, 2018; Bratkovič et al, 2020).

SNORDs interact with target RNAs via base pairings, which could influence 2′-O-methylation of RNA, pre-RNA processing, and RNA stability and/or translation (Falaleeva et al, 2016; Cavaillé, 2017; Huang et al, 2017; Monaco et al, 2018; Elliott et al, 2019; Bratkovič et al, 2020). Thirty human *SNORD116* elements comprise three groups based on their chromosomal location and sequence homology: *SNORD116-1* to *SNORD116-9* (group 1), *SNORD116-10* to *SNORD116-24* (group II), and *SNORD116-25* to *SNORD116-30* (group III). The group I *SNORD116s* are physically closest to the transcription start site and *SNURF-SNRPN* transcripts and are expressed at the highest levels among the constituent *SNORD116* groups (Runte et al, 2001; Castle et al, 2010; Cavaillé, 2017; Good & Kocher, 2017; Baldini et al, 2022; Fig 1A). The mouse *Snord116* elements are highly homologous to each other and share the highest sequence homology with human group I *SNORD116s*, especially in the region between C box and D′ box, that is, antisense element 1 (Good & Kocher, 2017; Fig 1B). The PWS minimal critical interval also contains *SNORD109A*, which is absent in the mouse syntenic region (Fig 1A and B). These specific differences are schematically illustrated in Fig 1.

About 600 genes are predicted to be *SNORD116* targets based on their primary sequence complementarity and secondary structures of the RNA targets (Bazeley et al, 2008; Bochukova et al, 2018). Several genes, including *NHLH2,* diacylglycerol kinase *kappa* (*DGKK*)*,* and *NLGN3*, display strong and phylogenetically conserved sequence complementation with *SNORD116*, and their expression levels were influenced by the *SNORD116* expression level in cultured cells (Kocher et al, 2021; Baldini et al, 2022). A recent study by Ye et al has implicated group II SNORD116 in 2′-O-methylation of *DGKK* mRNA in neurons derived from human embryonic stem cells (Ye et al, 2025 *Preprint*). However, none of these genes have been studied in PWS animal models, and evidence for direct interactions of *SNORD116* with its potential targets and the involvement of any of these potential *SNORD116* targets in the pathophysiology of PWS is modest.

This study investigated the molecular physiology of congenital *Snord116* deficiency in the mouse hypothalamus. Using two mouse models of *Snord116* deficiency—the *Snord116* KO and the paternal IC del models (Yang et al, 1998; Ding et al, 2008; Resnick et al, 2013)—we analyzed hypothalamic transcriptomes of postnatal day 5 (P5) mice to capture potential effects of *Snord116* deficiency in the proliferating neural progenitor populations (Wang et al, 2012; Maggi et al, 2014; Burbridge et al, 2016). We found that *Dgkk* was differentially expressed in both *Snord116* KO and IC del mice. *Dgkk* expression was confirmed by qRT–PCR to be elevated in the hypothalamus of *Snord116* KO mice relative to wild-type (WT) controls at all ages examined. We further investigated effects of *SNORD116* deficiency on protein expression, RNA stability, and 2′-O-methylation of *DGKK* in human cellular models. Compensatory mutation analysis confirmed that the predicted molecular interaction between *SNORD116* and *DGKK* was required for 2′-O-methylation of *DGKK*. We also examined the expression pattern of *Dgkk* in adult mouse brain, effects of fasting and diet-induced obesity on *Dgkk* expression in the mouse hypothalamus, and effects of *Snord116* deficiency on group 1 metabotropic glutamate receptor (mGluR1/5)–mediated PKC and ERK activation in primary mouse neurons. Elevated expression of diacylglycerol kinases (DGKs), including DGKK, which converts diacylglycerol (DAG) to phosphatidic acid (PA), could decrease DAG-mediated PKC and ERK activation in the intracellular signal transduction pathways—such as mGluR1/5—that employ DAG as a second messenger (see Fig 12 in Discussion) (Mao et al, 2022).

Our results substantiate existing evidence in the literature (Baldini et al, 2022; Ye et al, 2025 *Preprint*) and suggest that *DGKK* is a bona fide *SNORD116* target and that *SNORD116* deficiency affects mRNA and protein levels, as well as 2′-O-methylation of *DGKK,* with predicted functional consequences on intracellular signal transduction.

## Results

### Identification of hypothalamic genes and pathways affected by *Snord116* deficiency in mice

We conducted RNA-sequencing (RNAseq) analysis of the whole hypothalamus from P5 pups of two *Snord116*-deficient models: paternal *Snord116* deletion (*Snord116* KO) and paternal IC del (Yang et al, 1998; Ding et al, 2008). The maternally transmitted allele is silenced by imprint. *Snord116* KO mice do not express paternal *Snord116* because of a ∼150-kb deletion of the genomic region containing the *Snord116* cluster (Ding et al, 2008). IC del abolishes the expression of all paternally expressed genes in the mouse syntenic PWS region, including *Snord116* (Yang et al, 1998).

**Figure 2.**
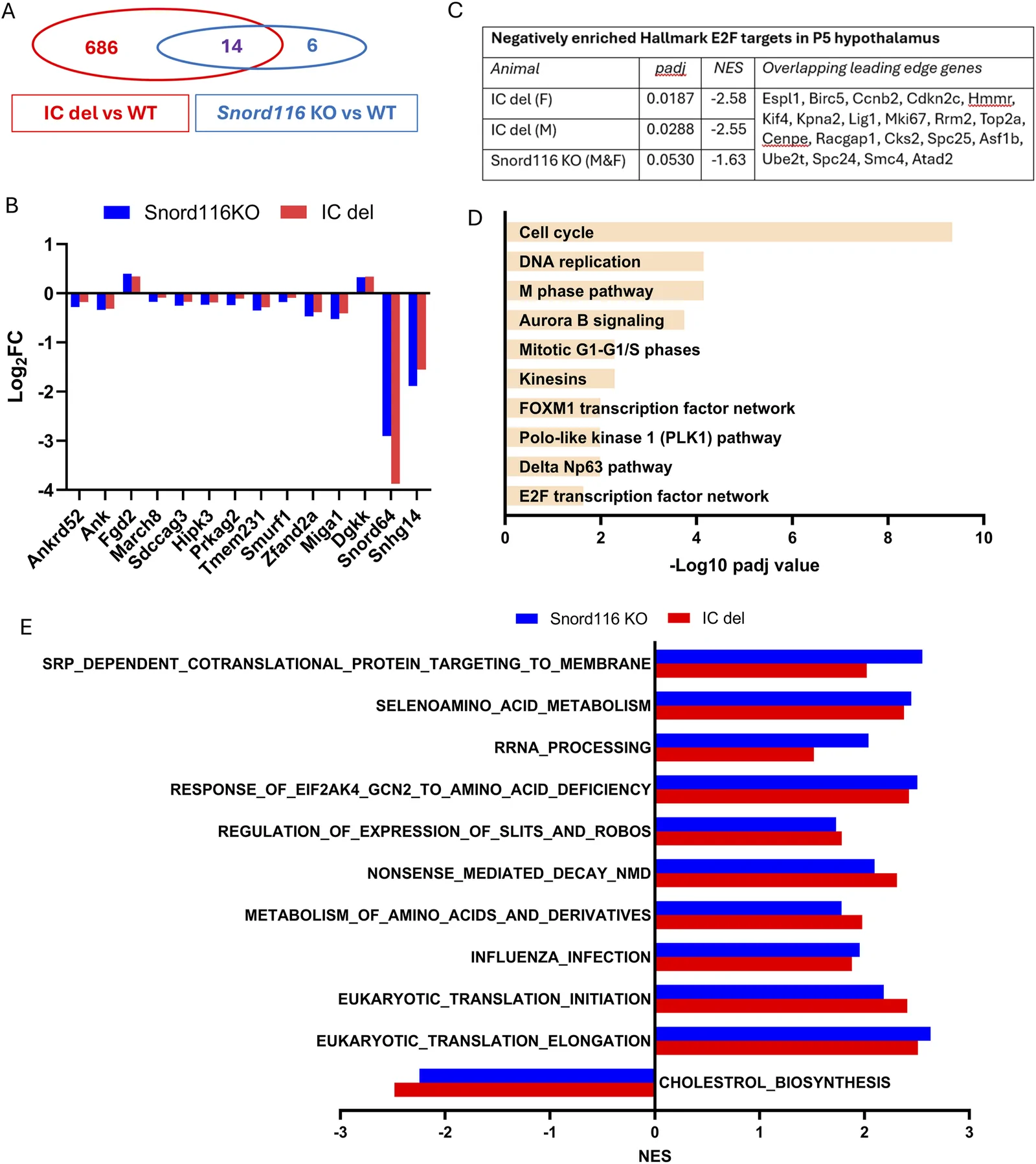
Comparative analyses of P5 hypothalamic transcriptomes of *Snord116* KO and IC del mouse models. **(A)** Numbers of DEGs in the hypothalamus of P5 IC del pups (red oval, n = 10, 5 M and 5F) and in the hypothalamus of P5 *Snord116* KO pups (blue oval, n = 5, 3M and 2F) compared with their respective WT littermates (FDR < 0.1). **(B)** Shared DEGs and their Log2FC in the P5 hypothalamus of IC del and *Snord116* KO pups relative to their respective WT controls. **(C)** Enrichment statistics of MSigDB Hallmark E2F_Targets in the P5 hypothalamus of *Snord116* KO and IC del mice relative to their respective WT controls and the overlapping leading-edge genes. NES, normalized enrichment score. **(D)** BioPlanet pathways enriched in the overlapping E2F_Targets leading-edge genes in (C) identified by Enrichr analysis. **(E)** Enriched Reactome pathways identified by GSEA shared by IC del and *Snord116* KO mice, all *padj* < 0.1, *P* < 0.01. NES, normalized enrichment score. Log2FC, Log2 fold change.

A total of 700 and 20 differentially expressed genes (DEGs, *FDR* < 0.1) were identified in IC del and *Snord116* KO mice, respectively, compared with their respective WT littermates (Fig 2A; Tables S1 and S2). Fourteen of these DEGs were changed in the same directions in both IC del and *Snord116* KO mice (Fig 2B). Among the shared DEGs, the two genes with the largest fold changes were *Snhg14* and *Snord64*, both of which are localized to the mouse syntenic PWS region on chromosome 7. *Snhg14*, the host gene of *Snord116*, was reduced in both IC del and *Snord116* KO mice as expected. *Snord64*, another paternally expressed gene in the mouse syntenic PWS region, was reduced in IC del mice as expected. However, the reduced expression of *Snord64* in *Snord116* KO mice was unexpected, as the deletion of the *Snord116* cluster does not overlap with the genomic region that encodes *Snord64*. These results suggest that deletion of the *Snord116* cluster may indirectly affect the expression of *Snord64* through secondary aberrant chromatin structure or functional alterations.

Table S1. DEGs in the P5 hypothalamus of IC del mice versus WT control (FDR < 0.1).

Table S2. DEGs in the P5 hypothalamus of Snord116 KO mice versus WT controls (FDR < 0.1).

Gene set enrichment analyses (GSEA) of RNAseq data were performed to identify biological processes and pathways affected by *Snord116* deficiency in the P5 hypothalamus (Mootha et al, 2003; Subramanian et al, 2005). Among the curated collection of MSigDB Hallmark gene sets (Liberzon et al, 2015), significant negative enrichments of E2F_Targets were observed in IC del mice (NES = −2.6, *padj* < 0.05 for both) and *Snord116* KO mice (NES = −1.63, *padj* < 0.1) (Fig 2C). There were also substantial overlaps in the E2F targets identified in IC del and *Snord116* KO mice; 20 shared leading-edge genes and their *t*-values are shown in Fig 2C and Table S3, suggesting that dysregulations of E2F targets in IC del and *Snord116* KO likely share *Snord116* deficiency as the etiology of these expression effects. E2F is a key regulator of cell cycle progression. Gene ontology analysis using Enrichr confirmed that the shared leading-edge E2F targets are involved in the regulation of cell cycle, DNA replication, M phase control, Aurora B signaling, kinesins, apoptotic DNA fragmentation, and tissue homeostasis (all *padj* < 0.05) (Fig 2D), suggesting that there is a decrease in cell proliferation in the P5 hypothalamus of these mutants. Aurora B signaling has also been implicated in axonal growth (Shlevkov et al, 2019; Blazejewski et al, 2021).

Table S3. t-Statistics (*t*-values) of the shared Hallmark_E2F_Targets leading-edge genes in the P5 hypothalamus of IC del and *Snord116* KO mice.

**Figure S1.**
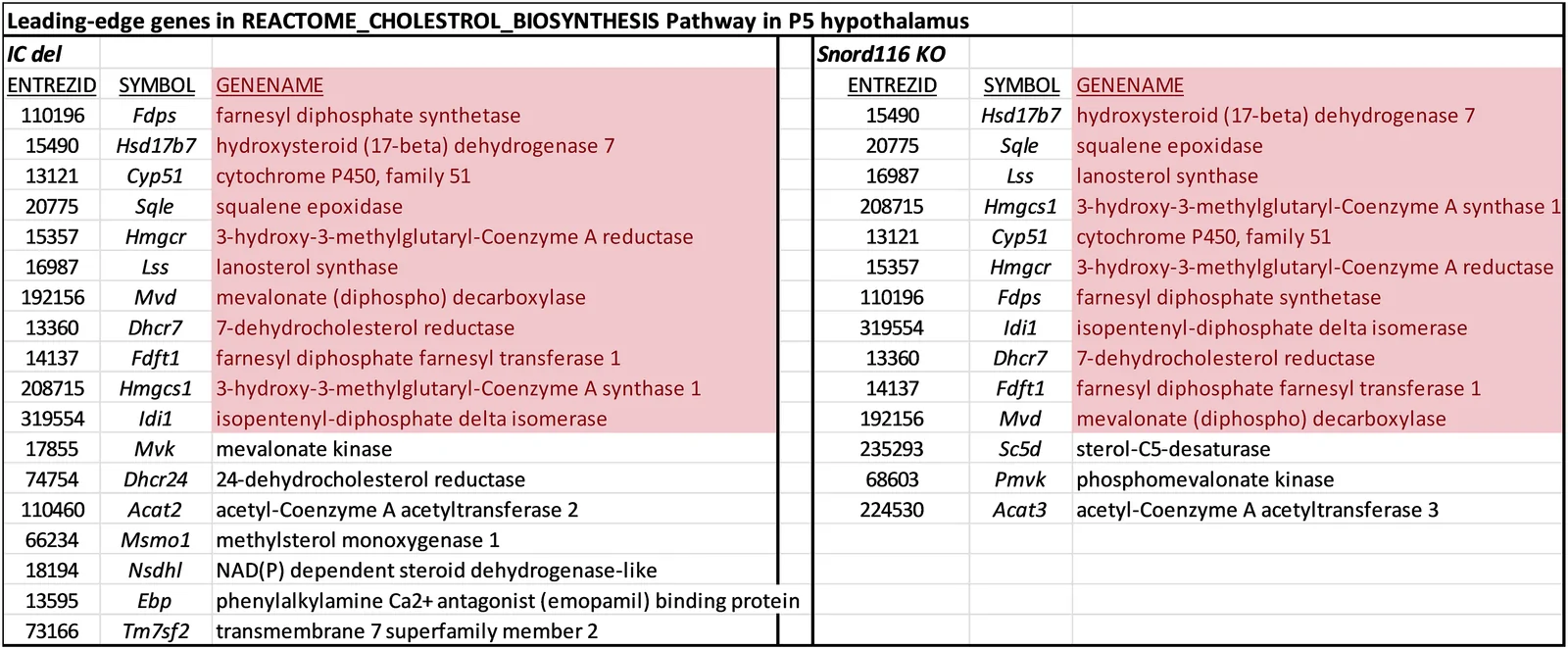
Leading-edge genes of the Reactome Cholesterol_Biosynthesis pathway in the P5 hypothalamus of IC del and *Snord116* KO mice. Shared leading-edge genes in IC del and Snord116 KO models are highlighted in red.

GSEA of the RNAseq data also identified enrichments of 135 and 37 Reactome pathways in IC del and *Snord116* KO mice, respectively (*padj* < 0.1) (Tables S4 and S5). Eleven pathways were enriched in the same direction in both models: amino acid and cholesterol metabolism, protein translation, SRP-dependent cotranslational protein targeting to membrane, and regulation of expression of Slits and Robos (all *padj* < 0.1) (Fig 2E). These pathways have been implicated in neurogenesis, neuron survival, and axon guidance (Andrews et al, 2008; Borrell et al, 2012; Kim et al, 2015b; Tong et al, 2019; Romanov et al, 2020). We also observed significant overlaps in the leading-edge genes of these shared pathways. In the Cholesterol_Biosynthesis pathway, which was negatively enriched in both IC del and *Snord116* KO mice, 11 out of 18 and 11 out 14 of the leading-edge genes in IC del and *Snord116* KO mice, respectively, were shared (Fig S1), again consistent with shared etiologic contributions. The Reactome pathway, Regulation_of_Cholesterol_Biosynthesis_by_SREBP-SREBF, was also negatively enriched in both IC del (NES = −1.74, *padj* = 0.09) and *Snord116* KO mice (NES = −1.9, *padj* = 0.17) (Tables S4 and S5), suggesting a coordinated down-regulation of cholesterol synthesis in these mutant mice. For the Protein_Translation and SRP-dependent_Co-translational_Protein_Targeting_To_Membrane pathways, which were positively enriched in both IC del and *Snord116* KO, changes in the expression levels of multiple ribosomal proteins were the primary factors contributing to the enrichments. Many of these ribosomal proteins (>30), as well as two elongation factors, *Eef2* and Eef1a1, and the gamma subunit of the ER protein translocation apparatus, *Sec61g*, were among the shared leading-edge genes in IC del and *Snord116* KO mice. *Isl1, Slit2, Robo2*, and *Msi2*, in addition to many ribosomal proteins, were among the shared leading-edge genes in the Regulation_of_Expression_of_Slits_and_Robos pathway (Tables S4 and S5). Furthermore, the Signaling_by_Robo_Receptors pathway was also positively enriched in both IC del mice (NES = 1.63, *padj* = 0.068) and *Snord116* KO mice (NES = 1.48, *padj* = 0.127) (Tables S4 and S5), suggesting that increased expression of Slits and Robos likely led to elevated downstream gene expression responses in IC del and *Snord116* KO mice. Slit-Robo signaling plays an important role in the production and positioning of periventricular dopamine neurons during hypothalamic development (Romanov et al, 2020).

Table S4. Enriched Reactome pathways in the P5 hypothalamus of IC del mice (padj < 0.1).

Table S5. Enriched Reactome pathways in the P5 hypothalamus of Snord116 KO mice (padj < 0.1).

### *SNORD116* deficiency is associated with elevated *DGKK* expression in mouse hypothalamus, human neuronal models, and induced pluripotent stem cells

**Figure S2.**
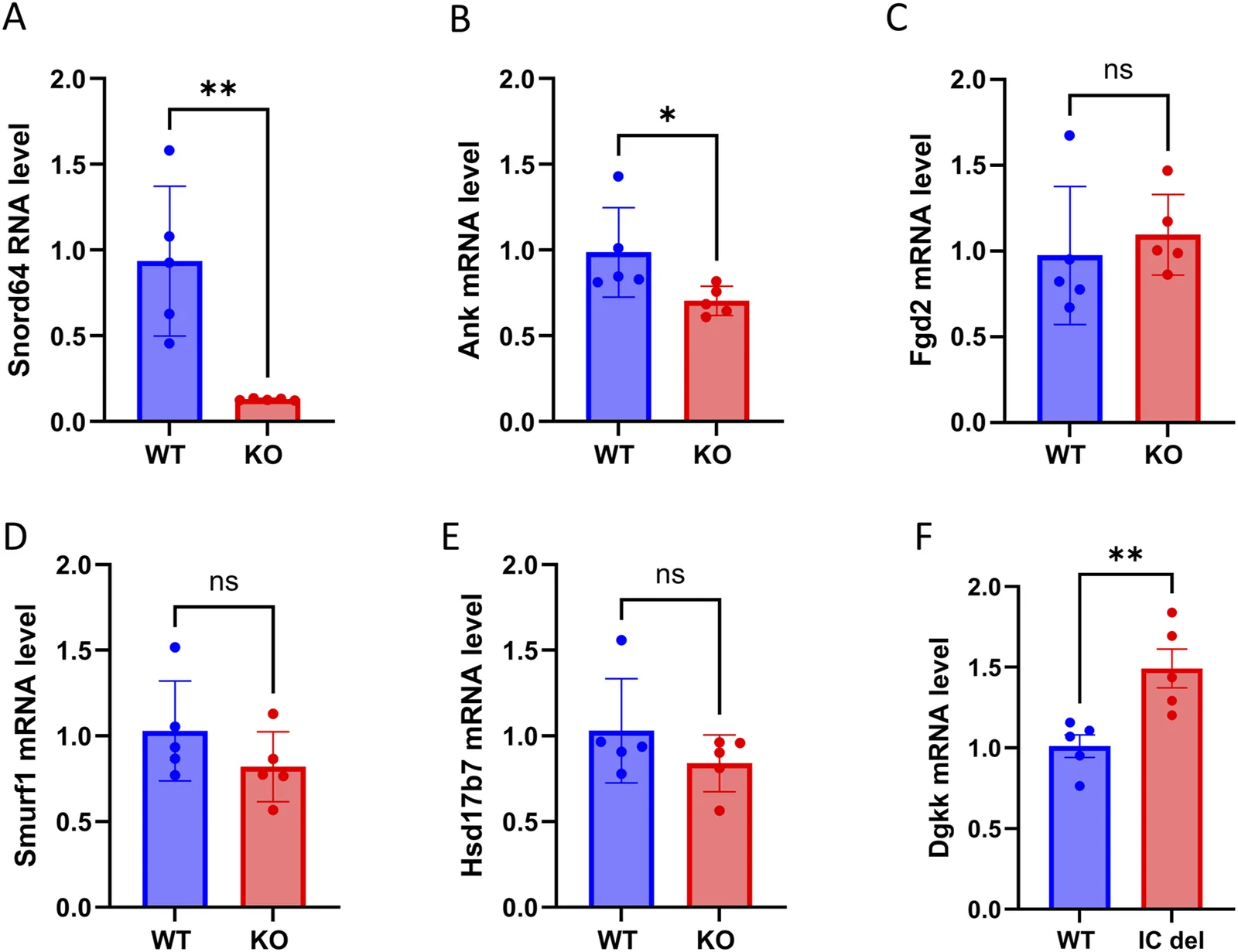
qRT–PCR analysis of the shared DEGs in P5 hypothalami of *Snord116* KO and IC del mice. **(A, B, C, D, E)** qRT–PCR analysis of expression levels of selected DEGs, *Snord64*, *Ank*, *Fgd2*, *Smurf1* (A, B, C, D), and *Hsd17b7*, a gene in the cholesterol biosynthesis pathway (E), in P5 hypothalami of *Snord116* KO mice and WT controls. * and ** indicate *P *< 0.05 and 0.01 respectively, *Snord116 *KO versus WT by two-tailed* t* test. **(F)** qRT–PCR analysis of *Dgkk* expression in P5 hypothalami of IC del mice and WT controls. ** indicates *P *< 0.01, IC del versus WT by two-tailed *t* test.

**Figure S3.**
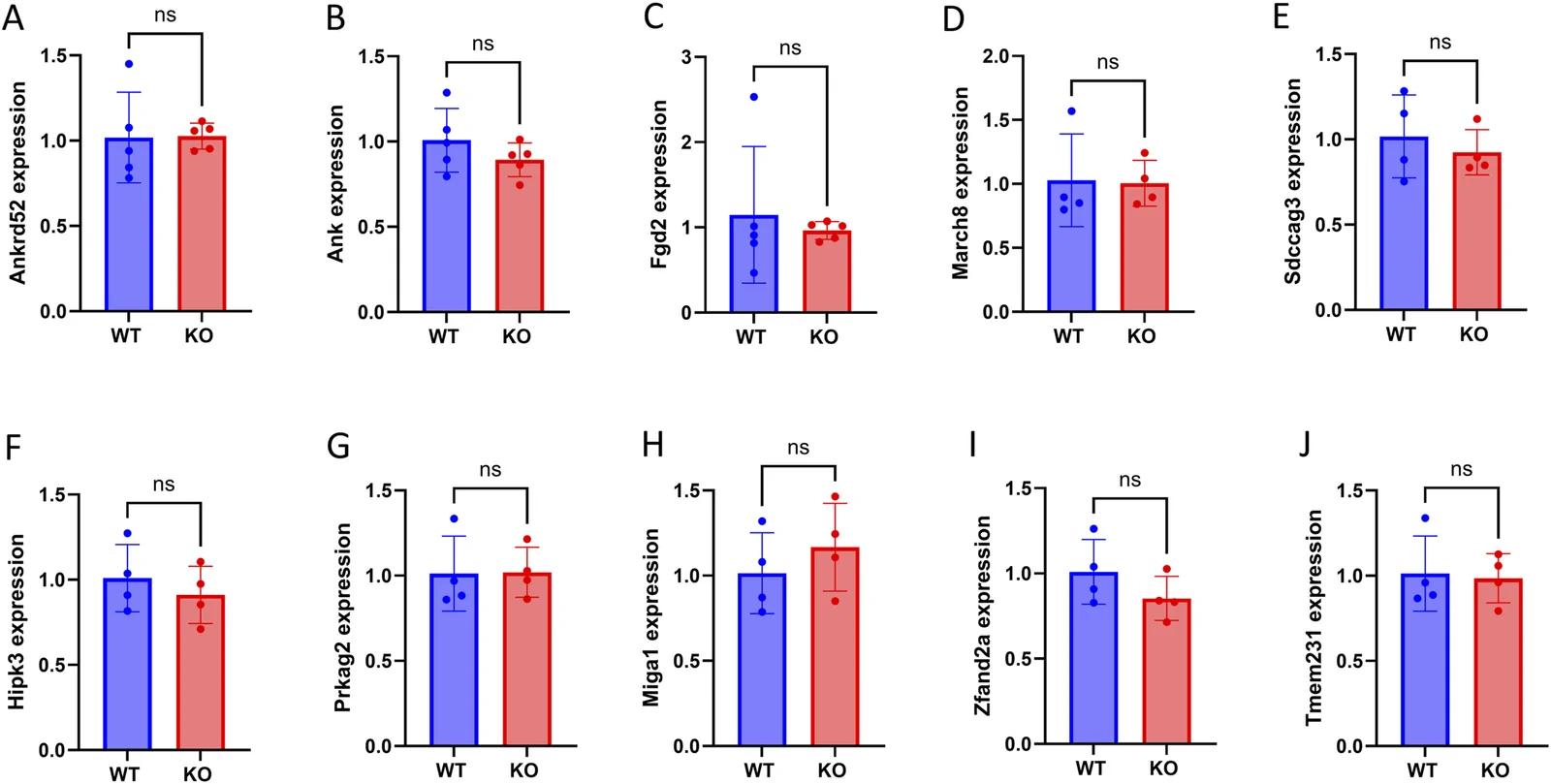
qRT–PCR analysis of the shared DEGs in 12-wk-old hypothalami of *Snord116* KO mice. **(A, B, C, D, E, F, G, H, I, J)** qRT–PCR analyses of expression levels of the shared DEGs in the hypothalamus of 12-wk-old male *Snord116* KO and sex-matched WT controls. Expression levels of *Ankrd52*, *Ank*, *Fgd2*, *March 8*, *Sdccag3*, *Hipk3*, *Prkag2*, *Miga1*, *Zfand2a*, and *Tmem231* are shown, respectively.

**Figure 3.**
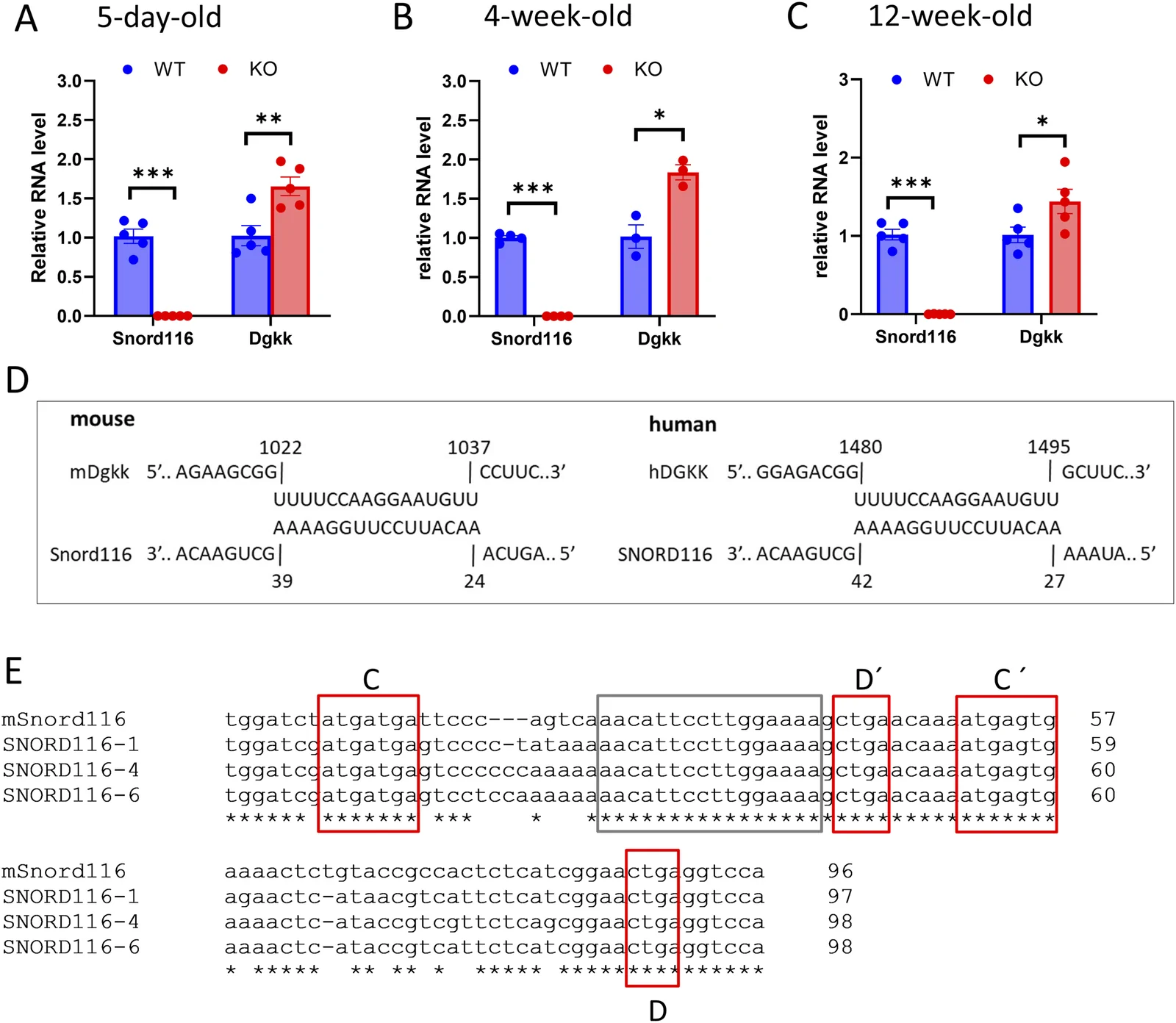
*Snord116* deficiency increases hypothalamic *Dgkk* transcript level from early postnatal period to adulthood and the predicted RNA:RNA interactions between *Snord116* and *Dgkk*. **(A, B, C)** qRT–PCR analysis of hypothalamic *Snord116* and *Dgkk* mRNA levels in 5-d-old (A), 4-wk-old (B), and 12-wk-old *Snord116* KO mice (C) and WT controls (n = 3–5). *, **, *** indicate *P* < 0.05, 0.01, and 0.001, respectively, *Snord116* KO versus WT by a two-tailed *t* test. **(D)** Predicted RNA:RNA interactions (base-pairing) between *Snord116* and *Dgkk* in mouse (left) and human (right) by IntaRNA 2.0. **(E)** Sequence alignments of the mouse *Snord116* and three human group 1 *SNORD116s*. The conserved C, D′, C′, and D boxes are boxed in red, and the predicted *Dgkk*-interacting region is boxed in gray.

Preliminary vetting of the shared DEGs in P5 hypothalamus of *Snord116* KO and IC del mice, including in silico analysis of RNA:RNA by IntaRNA 2.0 (Mann et al, 2017) and qRT–PCR analyses of gene expression in the mouse hypothalamus at different ages, was conducted to further evaluate these potential *Snord116* targets (Table S6, Figs S2A–F, and S3A–J). *Dgkk* was one of the top DEGs shared by IC del and *Snord116* KO mice, being increased in both IC del and *Snord116* KO mice relative to their respective WT controls (Log_2_FC of 0.37 and 0.33, *padj* < 0.05). qRT–PCR analyses showed that *Dgkk* mRNA levels were elevated by 1.7-, 1.8-, and 1.4-fold in 5-d-old, 4-wk-old, and 12-wk-old *Snord116* KO mice relative to their WT controls, respectively (all *P* < 0.05) (Fig 3A–C), indicating that the effect of *Snord116* deficiency on *Dgkk* expression persists from the early postnatal stage to adulthood. Consistent with results reported by Baldini et al (2022), IntaRNA 2.0 analysis detected a strong molecular interaction between the antisense element 1 of *Snord116* and exon 8 of *Dgkk* mRNA in both mouse and human with hybridization energies at −25.2 and −25.6 kcal/mol, respectively (Fig 3D). The nominal interacting sequences of both *Snord116* and *Dgkk* are 100% conserved in mice and human (group 1 *SNORD116s*) (Fig 3D and E). None of the other shared DEGs were consistently differentially expressed in the hypothalami of the mutant mice and their WT controls (Table S6, Figs S2A–F, and S3A–J).

Table S6. Summary of preliminary evaluation results of the shared DEGs in the P5 hypothalamus of Snord116 KO and IC del models.

**Figure 4.**
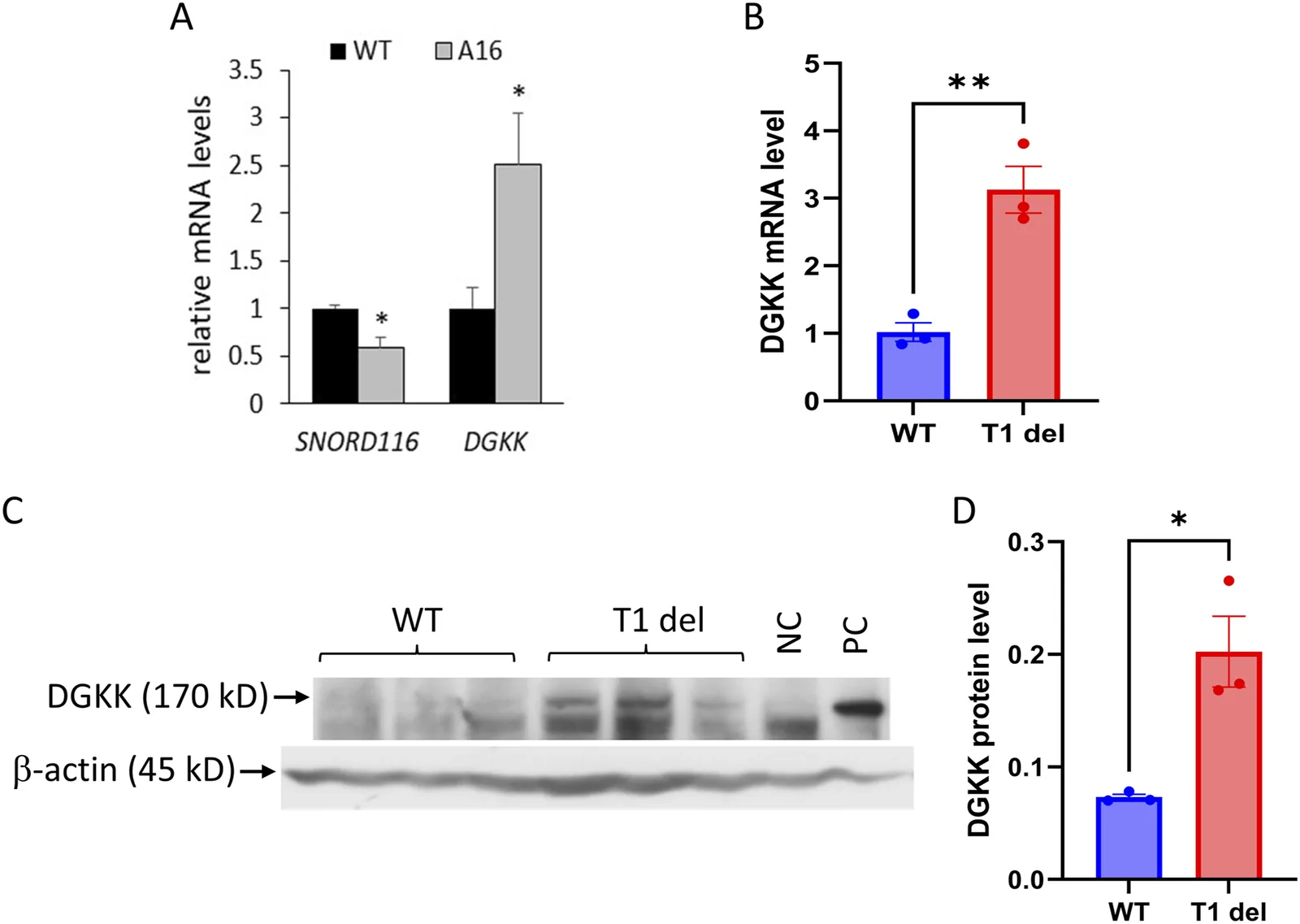
Effects of *SNORD116* deficiency on *DGKK* mRNA and protein levels in human cells. **(A)** qRT–PCR analysis of *SNORD116* and *DGKK* mRNA levels in the WT and A16 (*SNORD116* KD) SH-SY5Y cells (n = 3). * indicates *P* < 0.05, A16 versus WT by a two-tailed *t* test. **(B)** *DGKK* mRNA level in the normal control iPSCs (MGH2069) (WT) and the isogenic line with PWS T1 del. ** indicates *P *< 0.01, T1 del versus WT by two-tailed *t* test. **(C)** Western blots of the DGKK protein level and β-actin in the WT and T1 del iPSCs. NC: untransfected HEK293 cell lysate (30 μl); PC: cell lysate of HEK293 transfected with the hDGKK-FLAG plasmid (10 μl). **(D)** Quantification of the DGKK protein in the Western blot. DGKK protein levels were expressed as DGKK-to-β-actin ratios. * indicates *P* < 0.05, WT versus T1 del by a two-tailed *t* test in (B, D). NC, negative control; PC, positive control. Source data are available for this figure.

Consistent with the results from mouse hypothalamus, *DGKK* mRNA levels were 2.5-fold higher in an *SNORD116* knockdown (KD) human neuroblastoma SH-SY5Y cell line (A16) relative to the control SH-SY5Y line (Fig 4A). The A16 line was created using CRISPR-mediated deletion of a translocated *SNORD116* cluster, which results in ∼50% reduction in *SNORD116* expression in these cells (Bochukova et al, 2018). Human induced pluripotent stem cells (iPSCs) express high levels of *DGKK* and *SNORD116*. *DGKK* mRNA levels were elevated by 3.1-fold (*P* < 0.01) in an iPSC line with the PWS type 1 deletion (T1 del), which does not express *SNORD116* and all other paternally expressed genes in the region, relative to its isogenic normal control line (WT, MGH2069) (Fig 4B).

**Figure S4.**
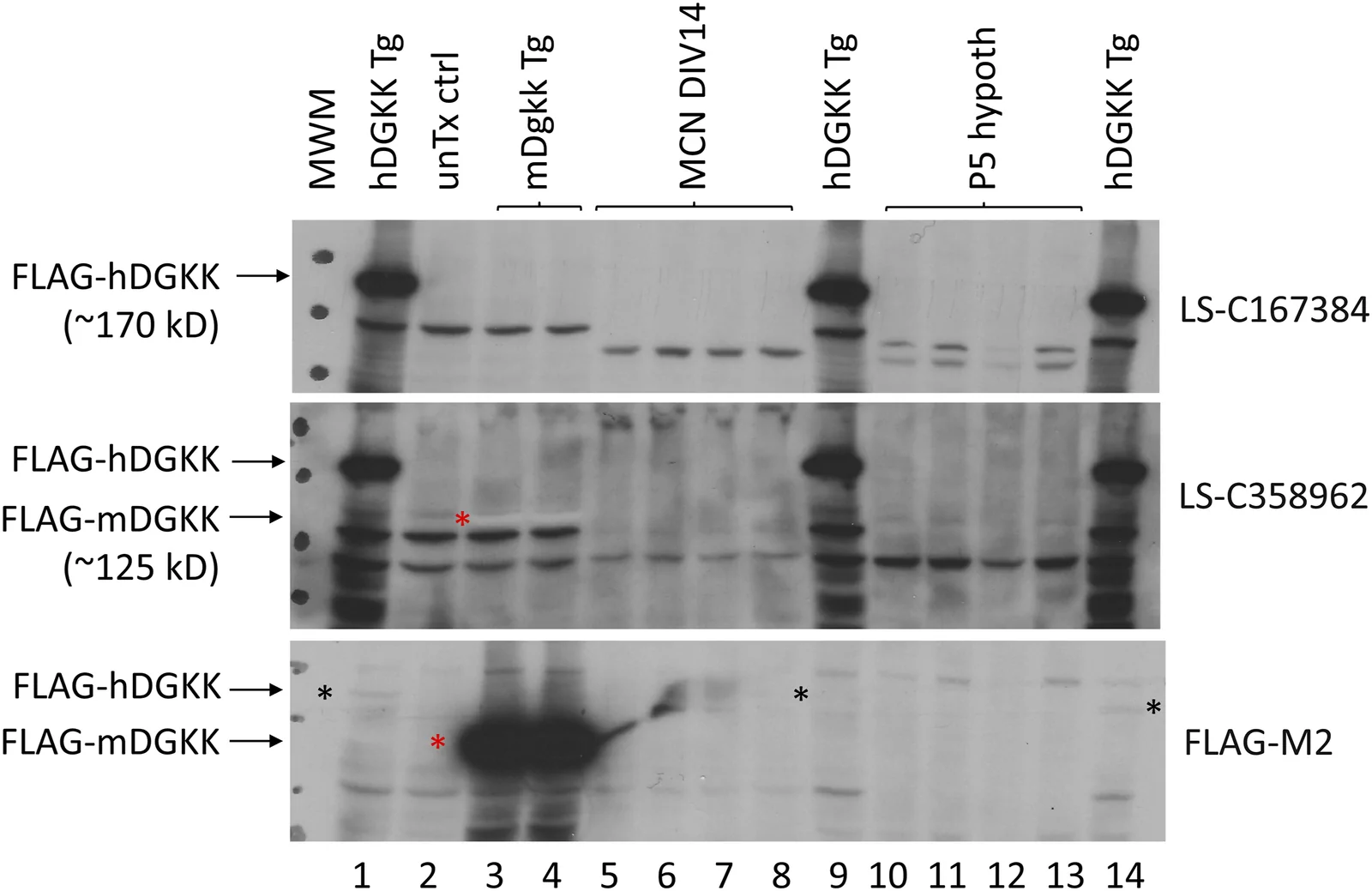
Validation of anti-DGKK antibodies to human DGKK by Western blotting. FLAG-tagged human and mouse DGKK were expressed in HEK293 cells by Lipofectamine 2000–mediated transfection of pcDNA3.1^+^hDGKK/C-FLAG or pcDNA3.1^+^mDGKK/N-FLAG. The FLAG-tagged hDGKK migrated near 170 kD (lanes 1, 9, 14), and the FLAG-tagged mDGKK (lanes 3 and 4) migrated near 125 kD. Lysates from DIV16 MCNs (lanes 5–8), lysates from P5 mouse hypothalamus (lanes 10-13), and the untransfected HEK293 cell lysate (lane 2, negative control) were also included in the Western blots. The same blot was sequentially blotted with LS-C167384 to DGKK, FLAG-M2 to the FLAG tag, and LS-C358962 to DGKK. The FLAG-tagged hDGKK was detected by LS-C167384, LS-C358962, and FLAG-M2 although the bands detected by anti-FLAG M2 antibody were weak. FLAG-M2 strongly detected the FLAG-tagged mDGKK, but the same bands were not detected by either LS-C167384 or LS-C358962 (marked by a red *). The molecular weight markers on the far-left lane are 250, 150, 100, and 75 kD, respectively.

**Figure S5.**
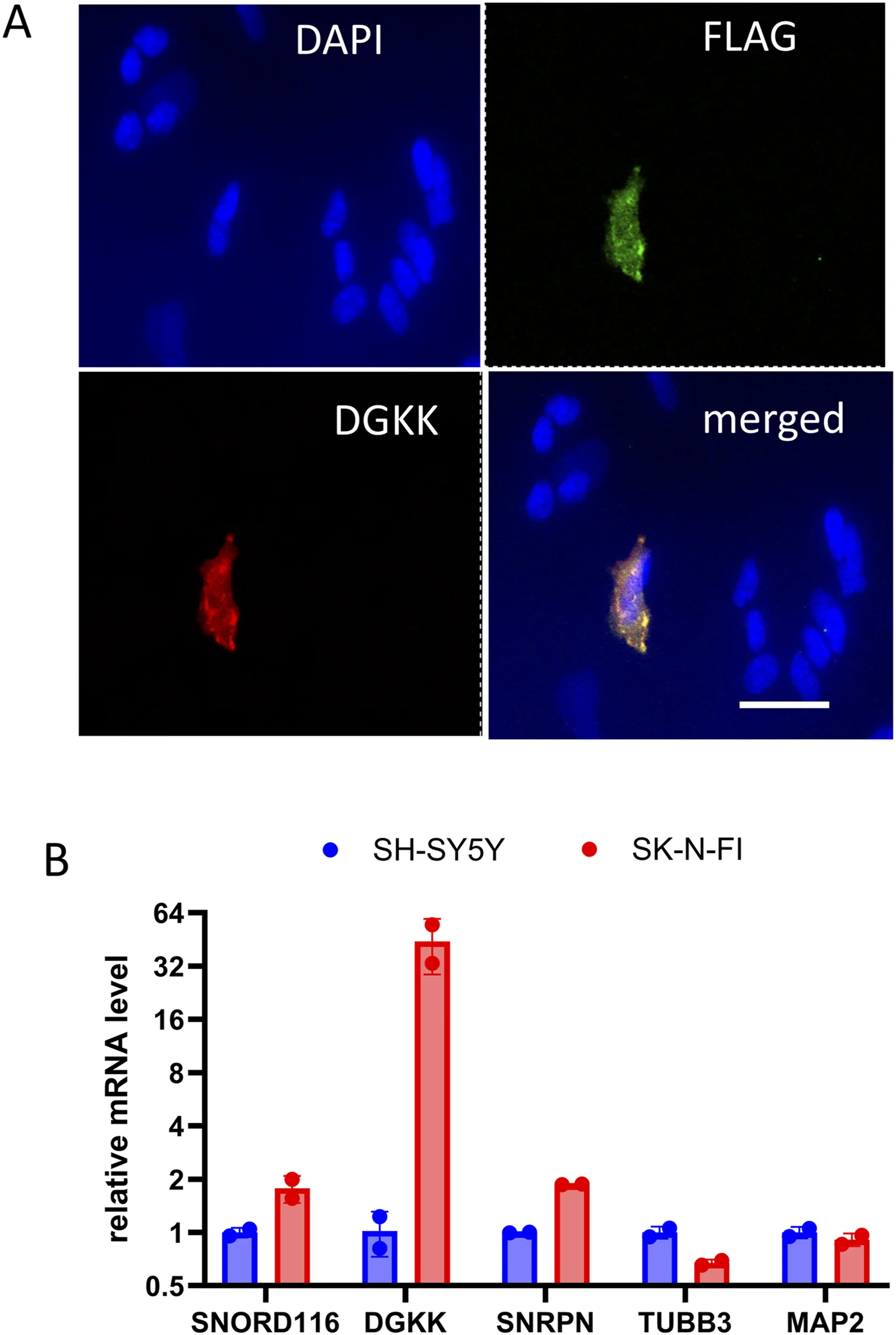
Validation of anti-DGKK antibodies to human DGKK by immunofluorescence staining. **(A)** FLAG-tagged human DGKK was expressed in SH-SY5Y cells by Lipofectamine 2000–mediated transfection of pcDNA3.1^+^hDGKK/C-FLAG. 72 h post-transfection, cells were fixed with 4% PFA in PBS for 10 min at room temperature and treated with 0.3% Triton X-100 to permeabilize the cells. Cells were then incubated with LS-C358962 rabbit polyclonal antibody and FLAG-M2 mouse monoclonal antibody overnight and developed with Alexa 656–conjugated anti-rabbit antibody and Alexa 488–conjugated anti-mouse antibody, respectively. Transfected cells expressing FLAG-tagged human DGKK were positive for both DGKK and the FLAG tag. The scale bar is 20 μm. **(B)** Comparison of the expression levels of *DGKK, SNORD116, SNRPN, TUBB3*, and *MAP2* in two neuroblastoma cell lines used in this study, SH-SY5Y and SK-N-FI. mRNA levels were normalized to the housekeeping genes *PPIA* and *TBP* and expressed as relative levels to those of SH-SY5Y cells, which were defined as 1.

We investigated the effect of *SNORD116* deficiency on DGKK protein levels. Using FLAG-tagged recombinant human and mouse DGKK proteins expressed in HEK293 and SH-SY5Y cells, we tested several commercially available DGKK antibodies. Two of the antibodies tested (LS-C167384 and LS-C358962) detected human DGKK by Western blotting and immunofluorescence (Figs S4 and S5A). By Western blotting, these antibodies and the anti-FLAG antibody (FLAG-M2) detected the same FLAG-tagged human DGKK protein migrating at ∼170 kD (Fig S4). The position of the FLAG-tagged human DGKK migrating on the SDS–PAGE that we detected is consistent with the previously reported result, which is significantly higher than the predicted molecular weight of the FLAG-tagged human DGKK, likely because of the N-terminal glutamate/proline-rich segment unique to the human DGKK protein (Imai et al, 2005). Immunofluorescence staining patterns by LS-C358962 and FLAG-M2 were superimposable in SH-SY5Y cells transfected with plasmids expressing FLAG-tagged human *DGKK* cDNA (Fig S5A). No endogenous DGKK protein in SH-SY5Y cells was detected by LS-C358962 in the immunofluorescence assay, likely because of the low DGKK expression levels in these cells (Fig S5A and B). Neither LS-C167384 nor LS-C358962 was able to detect the FLAG-tagged mouse DGKK protein in Western blots (Fig S4). A specific 170 kD DGKK band, like the FLAG-tagged human DGKK protein expressed in HEK293 cells, was also detected in human iPSCs using LS-C167384 (Fig 4C). Compared with the WT iPSCs, DGKK protein levels were elevated by 2.8-fold in the isogenic iPSCs with the PWS T1 del (*P* < 0.05) (Fig 4D). These results suggest that *SNORD116* negatively regulates *DGKK* mRNA and protein expression in both mice and humans.

**Figure 5.**
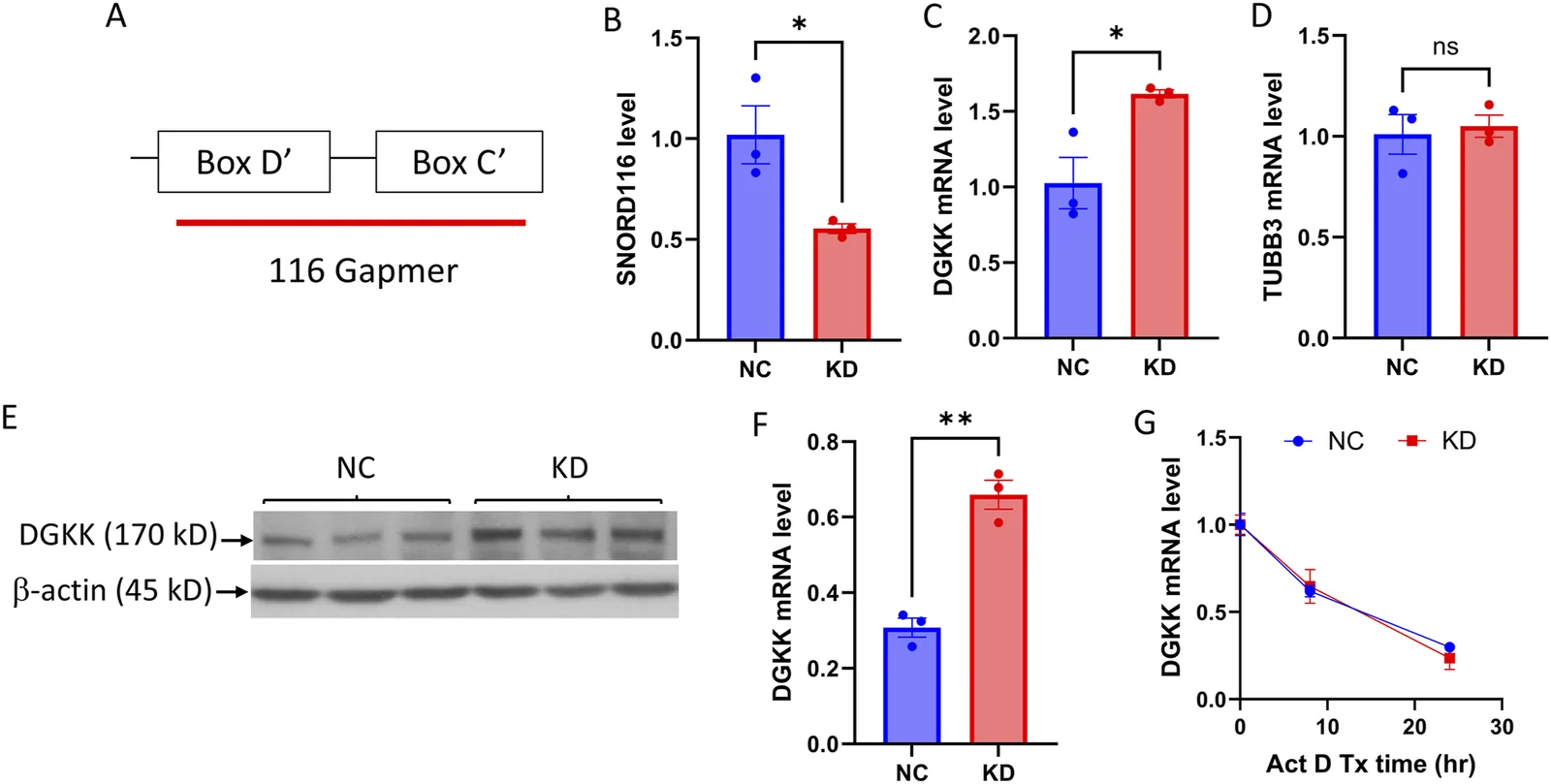
ASO-mediated *SNORD116* KD increases *DGKK* mRNA and protein levels in human neuroblastoma SK-N-FI cells. **(A)** Region of human *SNORD116* targeted by the ASO (116Gapmer) (Baldini et al, 2022). **(B, C, D)** Transcript levels of *SNORD116* (B), *DGKK* (C), and *TUBB3* (D) in *SNORD116* KD (KD) and control cells (NC) at 48 h post-transfection with the 116Gapmer and NC ASO. **(E)** Western blotting analysis of DGKK and β-actin protein levels in *SNORD116* KD (KD) and control cells (NC) at 48 h post-transfection. **(F)** Quantification of the DGKK protein in the Western blot. The DGKK protein level is expressed as DGKK-to-β-actin ratios. **(G)** *DGKK* mRNA decay rates in *SNORD116* KD (KD) and control cells (NC) after the treatment with the transcription inhibitor actinomycin D. Data are shown as the mean ± SEM of three technical replicas in a typical KD experiment. The experiments were repeated at least three times. * and ** indicate *P* < 0.05 and 0.01, respectively, KD versus NC by a two-tailed *t* test. NC, negative control.

**Figure S6.**
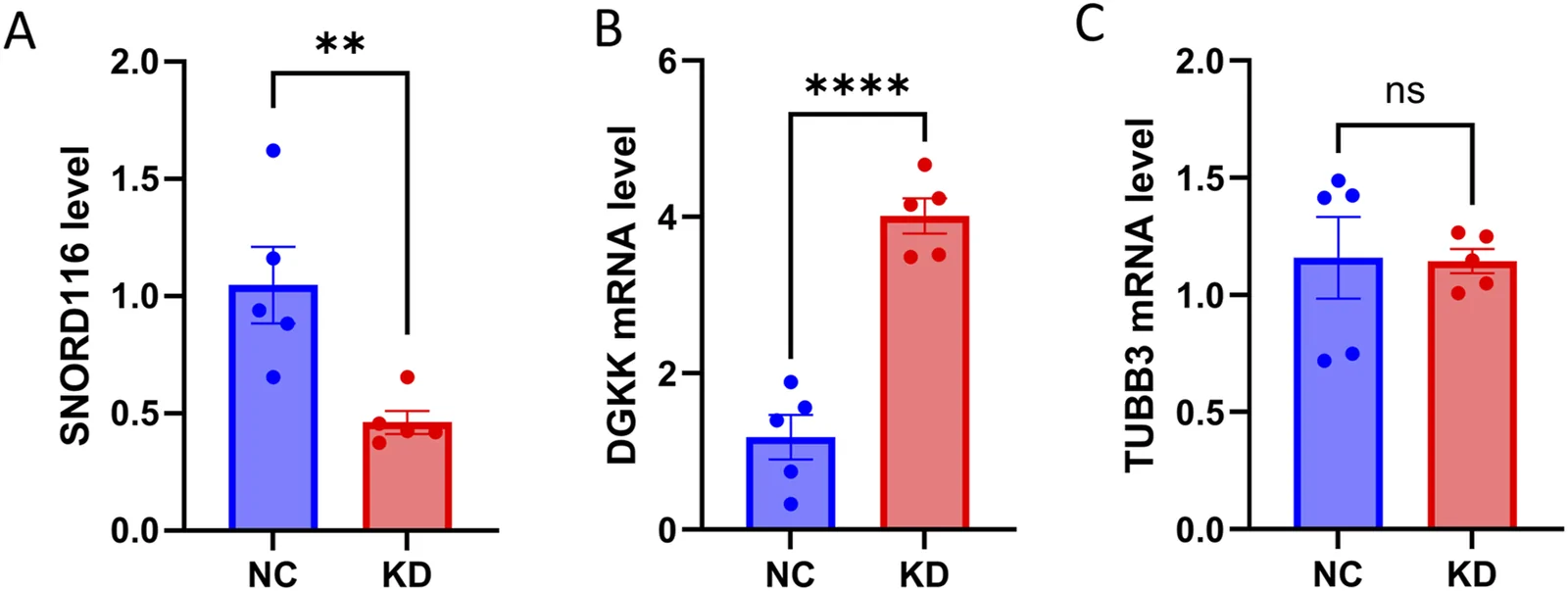
ASO-mediated *SNORD116* KD increases *DGKK* mRNA level in human neuroblastoma SH-SY5Y cells. **(A, B, C)** Transcript levels of *SNORD116* (A), *DGKK* (B), and *TUBB3* (C) in *SNORD116* KD (KD) and control cell (NC) at 24 h post-transfection of 116Gapmer and the NC ASO. Data are shown as the mean ± SEM. **, and **** indicate *P* < 0.01, and 0.0001, respectively, by two-tailed *t* test, KD versus NC. NC, negative control.

We examined the acute effects of *SNORD116* KD on *DGKK* mRNA and protein levels in human neuronal cell models. Transfection of human neuroblastoma SK-N-FI cells with a previously reported antisense oligo (ASO, 116Gapmer) (Baldini et al, 2022), targeting the sequence spanning the boxes D′ and C′ of group I and II *SNORD116* elements (Fig 5A), resulted in an ∼50% decrease in the *SNORD116* level with a concomitant 58% increase in *DGKK* mRNA levels and a 2.1-fold increase (*P* < 0.05) in DGKK protein levels, relative to the cells transfected with the negative control ASO (Fig 5B, C, E, and F). The transcript level of β3-tubulin (*TUBB3*), a neuronal marker, was not affected by *SNORD116* KD in these cells (Fig 5D). The effect of ASO-mediated *SNORD116* KD on *DGKK* mRNA expression in SK-N-FI cells was consistent with the result observed in HeLa S3 cells previously reported by Baldini et al (2022). To assess the mechanisms underlying the increased *DGKK* mRNA level associated with *SNORD116* deficiency, we assessed *DGKK* mRNA stability in the presence of the transcription inhibitor actinomycin D. After 8 and 24 h of actinomycin D treatment, the remaining fractions of *DGKK* mRNA relative to those at the beginning of actinomycin D treatment (0 h) were not significantly different between *SNORD116* KD and negative control cells (Fig 5G), suggesting that *SNORD116* KD had no significant effect on *DGKK* mRNA stability in SK-N-FI cells. Similar effects of acute *SNORD116* KD on *DGKK* mRNA were also observed in human neuroblastoma SH-SY5Y cells. Transfection of SH-SY5Y cells with 116Gapmer led to a 59% decrease (*P* < 0.01) in the *SNORD116* mRNA level, which was accompanied by a 3.4-fold increase in *DGKK* mRNA levels, relative to the cells transfected with the negative control ASO (Fig S6A and B). Again, ASO-mediated *SNORD116* KD had no significant effects on the transcript level of *Tubb3* in these cells (Fig S6C).

### *DGKK* mRNA is 2′-O-methylated in the predicted *SNORD116*-interacting region, and *SNORD116* deficiency decreases the 2′-O-methylation level

**Figure 6.**
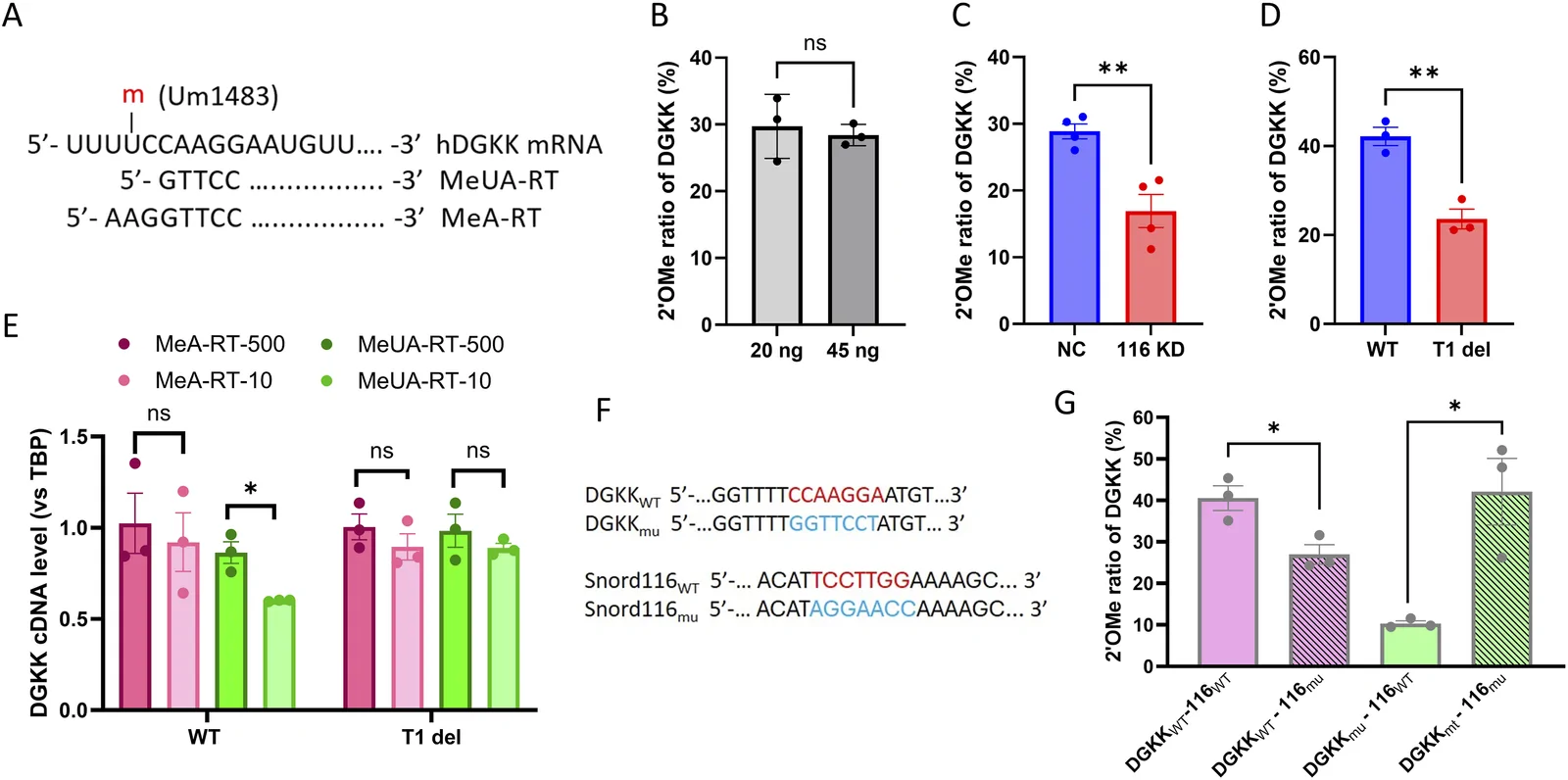
Predicted interaction between *SNORD116* and *DGKK* mRNA is required for 2′-O-methylation of U1483 of human *DGKK* mRNA. **(A)** Predicted 2′-O-methylation site (U1483) in the *SNORD116*-interacting region of human *DGKK* mRNA and the positions of 2-OMe anchored (MeA-RT) and unanchored (MeUA-RT) RT primers relative to U1483 used in the RTL-P assay. **(B)** Percentages of Um1483 of *DGKK* mRNA determined by the Nm-VAQ assay with two different RNA inputs. Data from three technical replicas are shown. **(C)** Effect of ASO-mediated KD of *SNORD116* (KD) on the percentage of Um1483 in SK-N-FI cells. Cells transfected with the NC ASO served as controls. **(D)** Percentages of Um1483 in the WT and PWS T1 del iPSCs determined by the Nm-VAQ assay. **(E)** RTL-P assay results of Um1483 in the WT and PWS T1 del iPSCs. **(F)** Seeding sequences of the WT and mutant *DGKK-FLAG* reporter gene and mouse *Snord116* in the predicted interacting sites of *DGKK* and *Snord116*. The WT sequences are in red letters and the mutant sequences in blue letters. **(G)** Compensatory mutation analysis results in HEK293 cells. Except (B), data from 3–4 experimental replicas are shown. ns, *, and ** indicate not significant, *P* < 0.05, and *P* < 0.01, respectively, between the two samples that are compared as shown by a two-tailed *t* test. NC, negative control.

A recognized function of SNORDs is to promote 2′-O-methylation of rRNA targets; for example, *SNORD27* promotes the 2′-O-methylation of A27 in 18S rRNA (Falaleeva et al, 2016). *SNORD116* associates with the protein complex responsible for 2′-O-methylation of rRNAs, that is, the methyltransferase fibrillarin (FBL), NOP56, NOP58, and NHP2L1 (Reichow et al, 2007), suggesting that *SNORD116* could potentially guide these enzymes to their 2′-O-methylation targets. *SNORD116*-dependent 2′-O-methylation of C1484 of *DGKK* mRNA, predicted to be mediated by group II *SNORD116*s, has been observed recently in neurons derived from human embryonic stem cells using RibOxi-seq (Ye et al, 2025 *Preprint*). Based on the canonical targets of SNORD/FBL-mediated 2′-O-methylation, group I *SNORD116*-guided 2′-O-methylation of human *DGKK* mRNA is predicted to occur at U1483 (NM_001013742.4), 5 base pairs upstream of the D′ box (CUGA) on the opposite strand (Figs 3D and 6A). Thus, we examined whether U1483 of *DGKK* mRNA is 2′-O-methylated and whether—as predicted—2′-O-methylation of U1483 is *SNORD116*-dependent.

**Figure S7.**
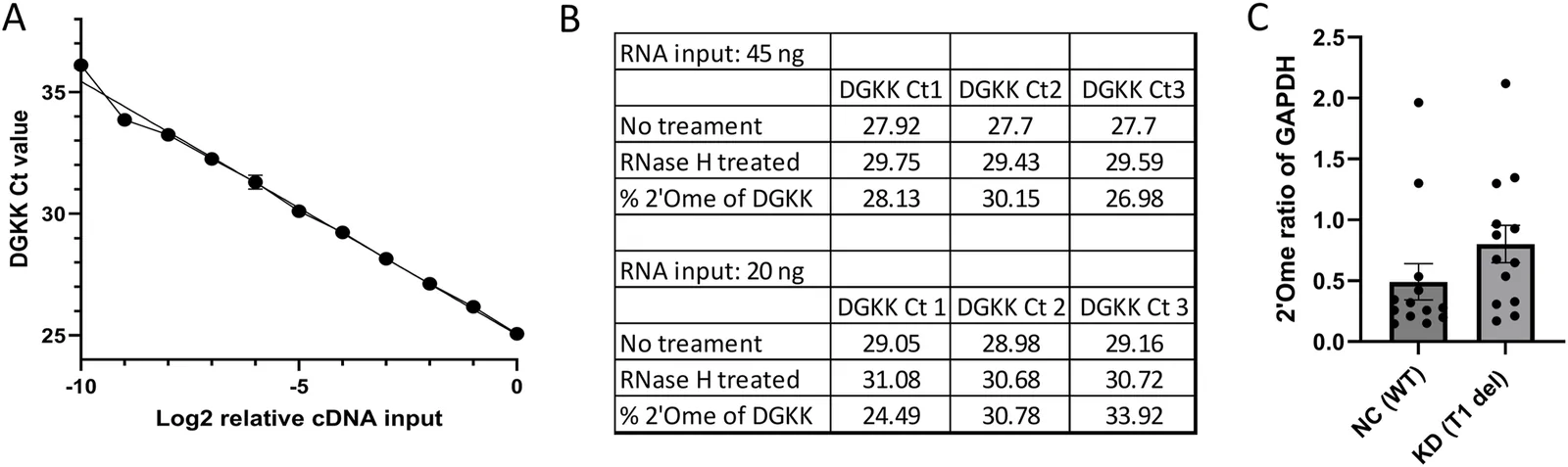
Validation and controls for Nm-VAQ assays. **(A)** Exponential amplification range of the SYBR Green–based *DGKK* qPCR assay. RT samples from SK-N-FI cells were subjected to serial dilations by the factor of 2. The serial diluted samples were then used in qPCR analysis using SYBR Green–based PCR reagents. Ct values of the serial diluted samples (in duplicates) were plotted against Log2 of relative cDNA (RT) inputs. **(B)** *DGKK* Ct values and the calculated percentages of Um1483 in *DGKK* mRNA (% 2′-OMe of DGKK) in two samples with cDNA inputs corresponding to 20 and 45 ng of the original total RNA inputs in an Nm-VAQ assay. qPCR analysis was performed in triplicates. **(C)** Percentages of 2′-OMe G585 of human *GAPDH* mRNA, which was used as a negative control in all Nm-VAQ assays. Data from multiple experiments, including *SNORD116* KD and *FBL* KD in SK-N-FI cells and WT and PWS T1 del iPSCs, were pooled.

We first used ribose 2′-O-methylation (Nm)-validation and quantification (Nm-VAQ) assay to investigate whether U1483 of human *DGKK* mRNA is 2′-O-methylated in SK-N-FI. The Nm-VAQ assay is based on the principle that RNA:DNA hybrid duplexes with a 2′-O-methylated nucleotide at the 3′- end of the duplex are protected from RNase H cleavage, whereas such hybrid duplexes with an unmodified nucleotide at the 3′-end the duplex are sensitive to RNase H cleavage (Yu et al, 1997; Tang et al, 2024). After the hybridization of total RNA with the *DGKK* chimeric probe specific to U1483, about 29% of *DGKK* mRNA was protected from RNase H digestion in SK-N-FI cells (Fig 6B). When the Ct values of *DGKK* in Nm-VAQ assays fell within the exponential amplification range of PRC reactions (25–33 cycles) (Fig S7A and B), Ct values of the technical replicas were tight and the proportion of 2′-O-methylated U1483 (Um1483), that is, the percentage of protection from RNase H digestion, was not affected by the amount of RNA input (Fig 6B). As a control, no appreciable amount of protection (average <1%) from RNase H digestion was observed for G585 of human *GAPDH* mRNA (NM_001289745.2) (Fig S7C), which has been previously shown not to have 2′-O-methylation at that nucleotide (Tang et al, 2024).

We then examined whether 2′-O-methylation of U1483 of *DGKK* mRNA is *SNORD116*-dependent in SK-N-FI cells. We found that the proportion of Um1483 was decreased by 40% in *SNORD116* KD cells (16.9%) relative to the proportion in the negative controls (28.8%) (Fig 6C), suggesting that *SNORD116* promotes/mediates 2′-O-methylation of U1483. We also determined the 2′-O-methylation status of U1483 in *DGKK* mRNA from the WT iPSC line (WT) and the isogenic line with PWS T1 del that were used above to determine *DGKK* mRNA levels (Fig 4B). The percentages of Um1483 were on average 42% and 25%, respectively, in the WT and T1 del iPSCs (Fig 6D), suggesting that 2′-O-methylation of U1483 in human iPSCs is also *SNORD116*-dependent. It was unexpected that ∼25% of *DGKK* mRNA was resistant to RNase H digestion in T1 del iPSCs because these cells do not express any *SNORD116*. It has been previously shown that the sensitivity of RNA:DNA hybrid duplexes to RNase H digestion may vary depending on the nucleotide sequence of RNA-DNA chimeric probes and their RNA targets (Chan et al, 2006; Hagedorn et al, 2017). Thus, it is possible that the residual methylation signal observed in T1 del iPSCs might result from incomplete digestion of the *DGKK* (U1483) RNA:DNA hybrid duplexes. The presence of *SNORD116*-dependent 2′-O-methylation of U1483 in iPSCs was confirmed independently using the RTL-P assay, another site-specific 2′-O-methylation assay (Dong et al, 2012). The RTL-P assay is based on the principle that 2′-O-methylation of a ribonucleotide renders reverse transcription (RT) of the RNA less efficient at a low but not at a high concentration of deoxynucleotides (dNTP). We performed the RTL-P assay using an anchored (MeA-RT) and an unanchored (MeUA-RT) primer in the RT reactions. The positions of these RT primers relative to U1483 are shown in Fig 6A. When RT reactions were primed with the unanchored primer, the amounts of *DGKK* cDNA generated from RNA samples isolated from WT iPSCs were decreased by 30% at 10 μM dNTP (0.601 ± 0.003) compared with those generated at 500 μM dNTP (0.864 ± 0.059) (*P* < 0.05) (Fig 6E). In contrast, the amounts of *DGKK* cDNA generated from RNA samples isolated from T1 del iPSCs were not significantly affected by the dNTP concentration (0.890 ± 0.025 at 10 μM vs. 0.983 ± 0.090 at 500 μM, *P* = 0.37). As controls, when RT reactions were primed with the anchored primer, dNTP concentration had no significant effect on the amount of *DGKK* cDNA generated from RNA samples isolated from either WT or T1 del iPSCs (Fig 6E), although the amounts of *DGKK* cDNA generated at 10 μM dNTP were slightly lower than those generated at 500 μM in both WT and T1 del iPSCs. The RTL-P results are fully consistent with a role for *SNORD116* in the methylation status of *DGKK* mRNA.

To further assess whether the predicted interaction between *SNORD116* and *DGKK* is required for the apparent *SNORD116*-dependent 2′-O-methylation of U1483, we performed compensatory mutation analysis in HEK293 cells, which do not express endogenous *DGKK* or *SNORD116*, using the *DGKK-FLAG* reporter gene and a plasmid expressing the mouse *Snord116* previously reported by Falaleeva et al (2015). As shown in Fig 3D and E, mouse *Snord116* shares full sequence homology with group I human *SNORD116* in the predicted *DGKK*-interacting site. Mutation of the 7-bp seeding sequence (WT sequence in red and mutant in blue letters) in the respective predicted interacting site in either *DGKK* or *Snord116* would be expected to disrupt the predicted interaction between *DGKK* and *Snord116*, and therefore 2′-O-methylation of U1483. Conversely, mutation of the 7-bp seeding sequences in both *DGKK* and *Snord116* would restore the sequence complementarity between *DGKK* and *Snord116* and restore the predicted interaction between the two molecules and cause 2′-O-methylation of U1483 (Fig 6F). Consistent with the predictions, 2′-O-methylation of U1483 was markedly decreased in cells cotransfected with the combinations of *DGKK*_WT_/*Snord116*_mu_ or *DGKK*_mu_/*Snord116*_WT_ constructs, compared with those in cells cotransfected with *DGKK*_WT_*/Snord116*_WT_ constructs (Fig 6G). Importantly, 2′-O-methylation of *DGKK* was restored in cells cotransfected with *DGKK*_mu_/*Snord116*_mu_ constructs (Fig 6G). Residual 2-OMe signals on U1483 were observed in cells transfected with *DGKK*_WT_/*Snord116*_mu_ or *DGKK*_mu_/*Snord116*_WT_ constructs, again suggesting a possible suboptimal digestion of the *DGKK* (U1483) RNA:DNA hybrid duplexes by RNase H. Despite the technical limitation of the Nm-VAQ assays, the compensatory mutation analysis results confirmed that the sequence complementation between *DGKK* and *Snord116* at the predicted interacting site is important in mediating 2′-O-methylation of U1483 of *DGKK* mRNA.

**Figure 7.**
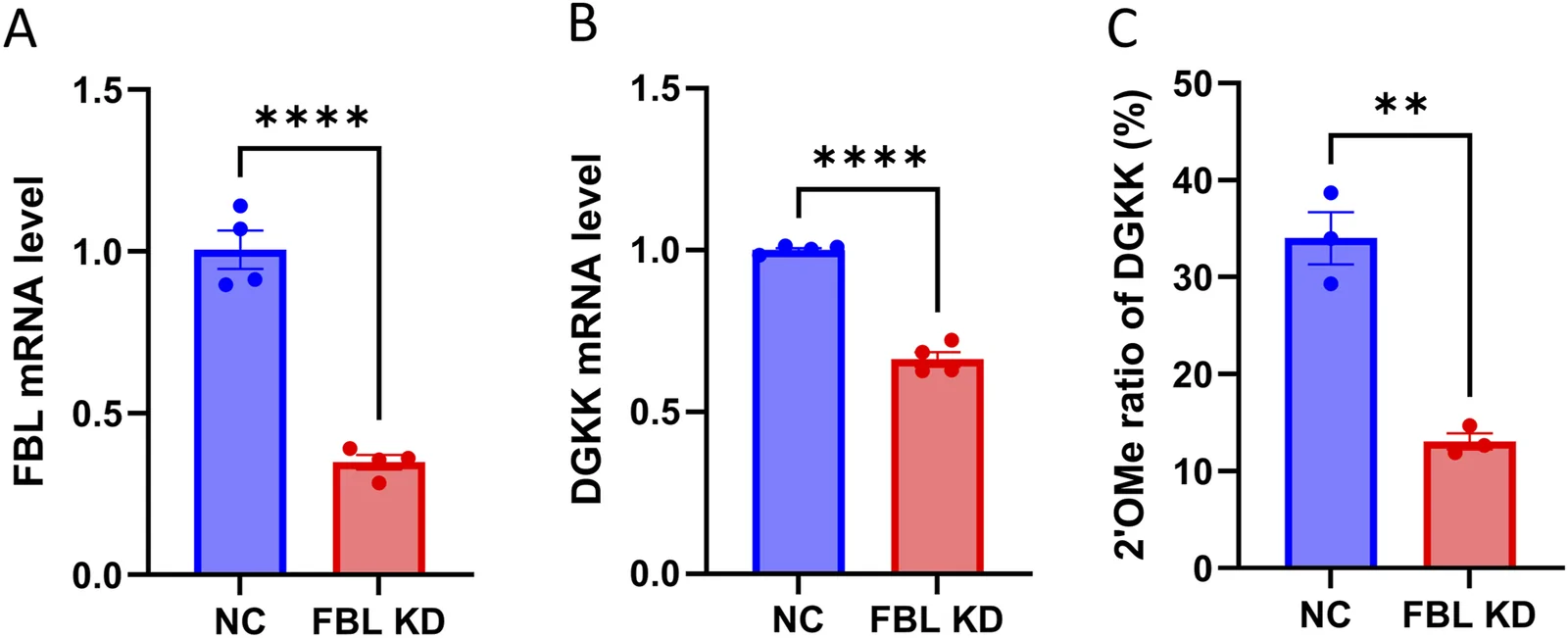
Effects of FBL KD on *DGKK* mRNA expression and 2′-O-methylation of U1483 of *DGKK* mRNA. **(A, B)** siRNA-mediated *FBL* KD and the effects on *FBL* (A) and *DGKK* (B) expression in SK-N-FI cells. Results of a typical *FBL* KD experiment with four technical replicas are shown. **(C)** Effects of *FBL* KD on 2’-O-methylation of U1483 in *DGKK* mRNA in SK-N-FI cells. Results from three independent Nm-VAQ assays are shown. Cells transfected with the NC siRNA (NC) served as controls. ** and **** indicate *P* < 0.01 and 0.0001, respectively, *FBL* KD versus NC by a two-tailed *t* test (A, B, C). NC, negative control.

*FBL*, a RNA 2′-O-methyltransferase, is a critical component of many SNORD-guided ribonucleoprotein complexes (snoRNPs) that catalyze 2′-O-methylation of rRNA, tRNA, snRNA, and mRNAs (Kiss-László et al, 1996; Falaleeva et al, 2016; Monaco et al, 2018; Elliott et al, 2019). We investigated whether *FBL* is involved in 2′-O-methylation of U1483 of human *DGKK* mRNA. siRNA-mediated *FBL* KD decreased *FBL* and *DGKK* mRNA levels by ∼65% and 34%, respectively (Fig 7A and B). The percentage of 2′-O-methylation of U1483 decreased from 29.3% in the negative controls to 11.9% in *FBL* KD cells (Fig 7C), indicating that *FBL* is involved in the 2′-O-methylation of U1483. However, the discordant effects of *SNORD116* KD and *FBL* KD on the *DGKK* mRNA level in SK-N-FI cells suggest that 2′-O-methylation of U1483 may not be the primary mechanism underlying elevated *DGKK* expression associated with *SNORD116* deficiency. snoRNAs are known to form both canonical and noncanonical snoRNPs that perform multiple cellular functions, including pre-RNA processing, such as polyadenylation and splicing, independent of or in addition to guiding 2′-O-methylation of their targets (Gerbi, 1995; Falaleeva et al, 2016; Huang et al, 2017; Ojha et al, 2020; Han et al, 2022). These functions are not mutually exclusive. Based on cell morphology and total RNA yields (Table S7), no apparent cellular toxicity associated with *FBL* KD was observed in SK-N-FI cells.

Table S7. Total RNA yield (ng) from *FBL* KD and negative control SK-N-FI cells with 4 experimental replicas in a typical *FBL* KD experiment.

### *Dgkk* is expressed in mouse brain regions key to the regulation of energy balance, feeding behavior, and circadian rhythm

**Figure S8.**
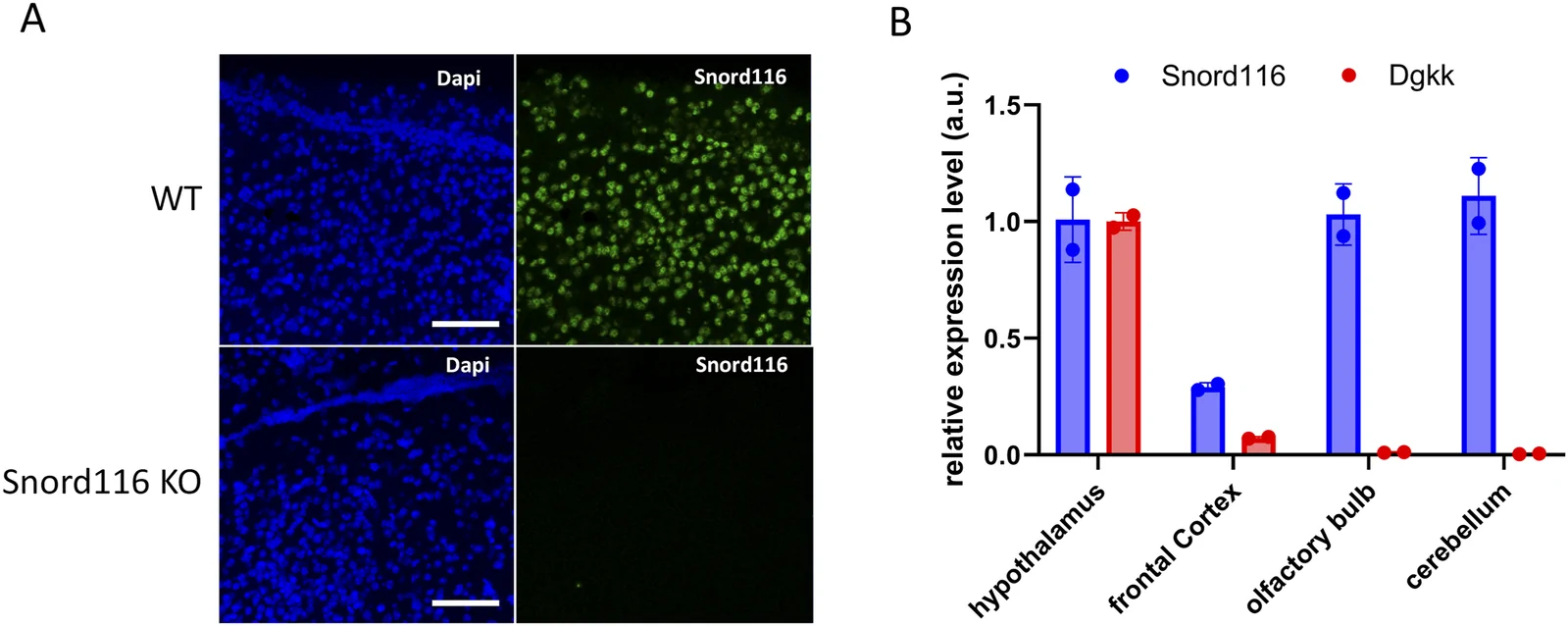
Validation of the RNAscope probes for mouse *Snord116* and *Dgkk*. **(A)** Detection of *Snord116* in the WT brain section but not in the *Snord116* KO brain section using the RNAscope probe specific to mouse *Snord116*. DAPI staining shows the nuclei in WT and *Snord116* KO brain sections. Scale bars are 50 μm. **(B)** qRT–PCR analysis of the expression of *Dgkk* and *Snord116* in the indicated brain regions of 8-wk-old male WT mice. The qRT–PCR results are consistent with the expression patterns of *Dgkk* and *Snord116* detected by RNAscope (Figs 8 and S9).

**Figure 8.**
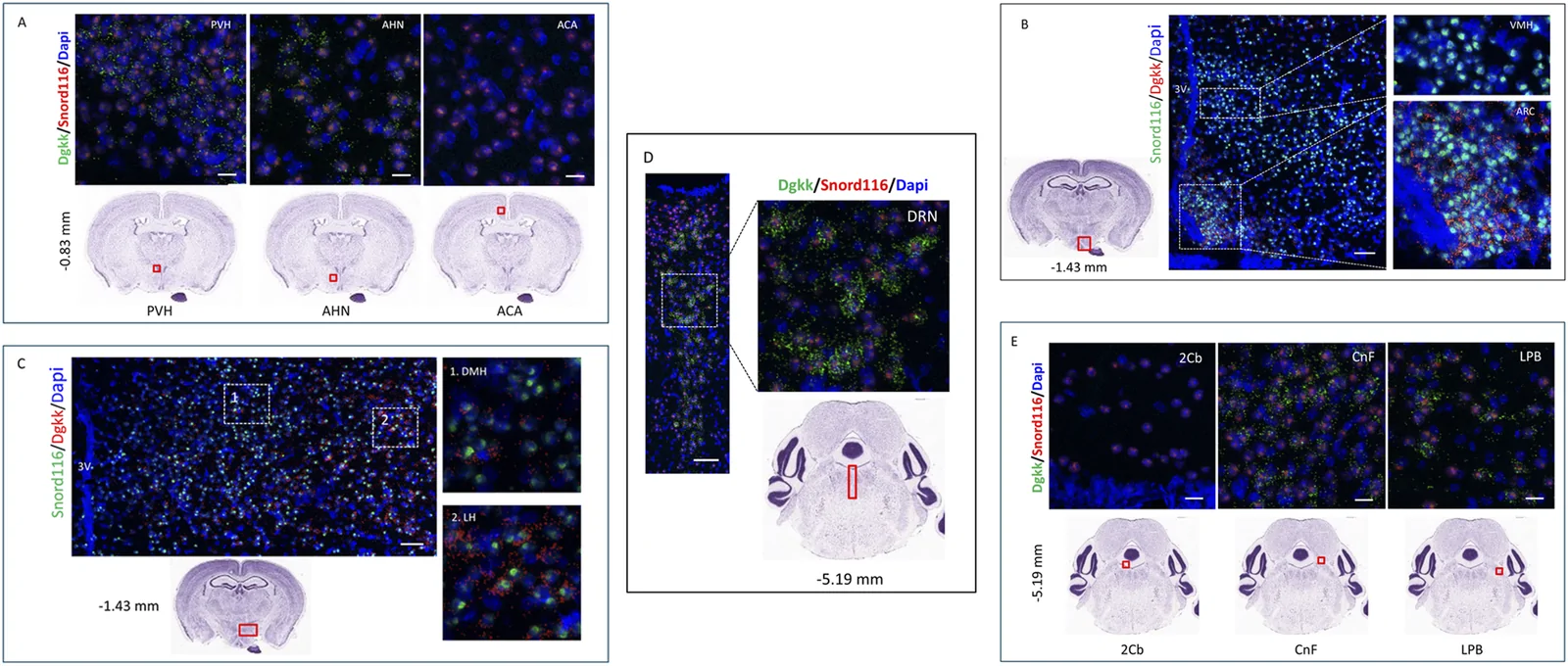
*Dgkk* is expressed in brain regions implicated in regulating feeding behavior and energy balance. **(A)** *Snord116* and *Dgkk* staining in the PVH, AHN, and ACA in the same coronal section. PVH and AHN express high and moderate levels of *Dgkk*. **(B)** *Snord116* and *Dgkk* staining in the arcuate (ARC) and ventromedial (VMH) nuclei of the hypothalamus. *Dgkk* is highly expressed in the ARC but not in the adjacent VMH. **(C)** *Snord116* and *Dgkk* staining in the lateral (LH) and dorsomedial (DMH) nuclei of the hypothalamus in the same section. LH and DMH express high and moderate levels of *Dgkk*, respectively. **(D)** *Snord116* and *Dgkk* staining in the DRN. DRN expresses one of the highest levels of *Dgkk* in the mouse brain. **(E)** *Snord116* and *Dgkk* staining in the 2Cb, CnF, and LPB nucleus of the same section. *Dgkk* is expressed at high levels in CnF and LPB, but not in 2Cb. *Snord116* and *Dgkk* staining are either in red and green or in green and red as indicated in each panel. Nuclei were stained blue by DAPI. Scale bars are 20 μm in (A) and (E) and 50 μm in (B, C, D), respectively. The gross anatomic regions of *Snord116*- and *Dgkk*-stained images are indicated by the red boxes on the corresponding Nissl-stained brain sections. The distance of each section to the bregma is indicated next to the Nissl-stained section. 3V, 3rd ventricle. RNAscope was performed on brain sections from 9-wk-old male C57BL/6J mice. PVH, paraventricular nucleus of the hypothalamus; AHN, anterior hypothalamic nucleus; ACA, anterior cingulate area; DRN, dorsal raphe nucleus; 2Cb, lobule 2 of the cerebellar vermis; CnF, cuneiform nucleus; PLB nucleus, lateral parabrachial nucleus.

**Figure S9.**
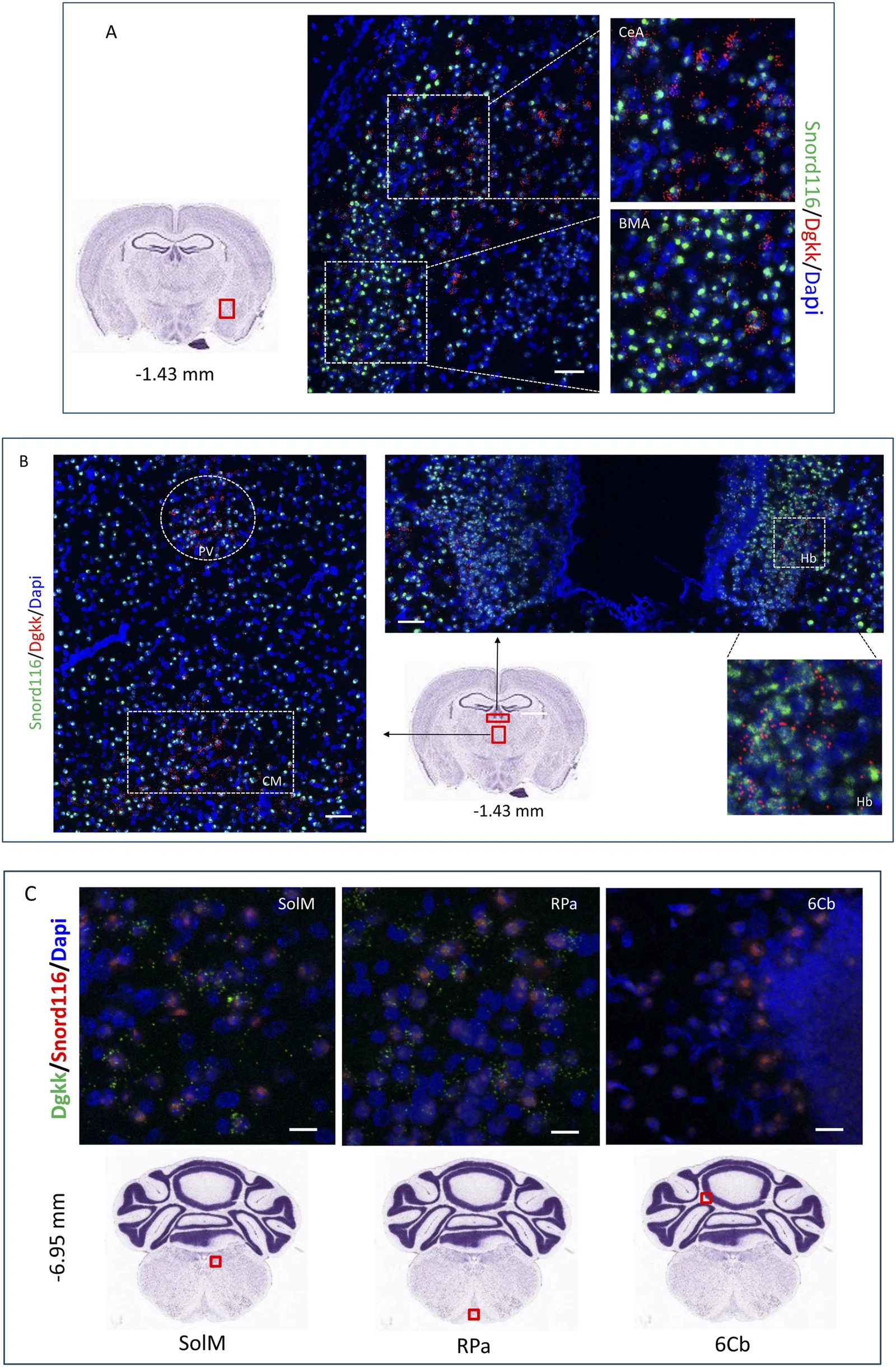
Expression of *Dgkk* and *Snord116* in various mouse brain regions. **(A)** *Snord116* and *Dgkk* staining in the central amygdaloid (CeA) nucleus and basomedial amygdaloid (BMA) nucleus. **(B)** *Snord116* and *Dgkk* staining in the paraventricular (PV) thalamic nucleus, central medial (CM) thalamic nucleus, and medial/lateral habenular (Hb) nucleus. **(C)** *Snord116* and *Dgkk* staining in the medial part of the solitary nucleus (SolM), raphe pallidus (RPa) nucleus, and lobule 6 of the cerebellar vermis (6Cb) on the same coronal section. *Snord116* and *Dgkk* staining were in green and red or red and green as indicated in each panel. Nuclei were stained blue by DAPI. Scale bars are 50 μm. The gross anatomic regions of the *Snord116*- and *Dgkk*-stained images are indicated by the red boxes on the Nissl-stained brain section(s) shown in each panel. The distance of the section to the bregma is indicated next to the Nissl-stained section(s).

**Figure 9.**
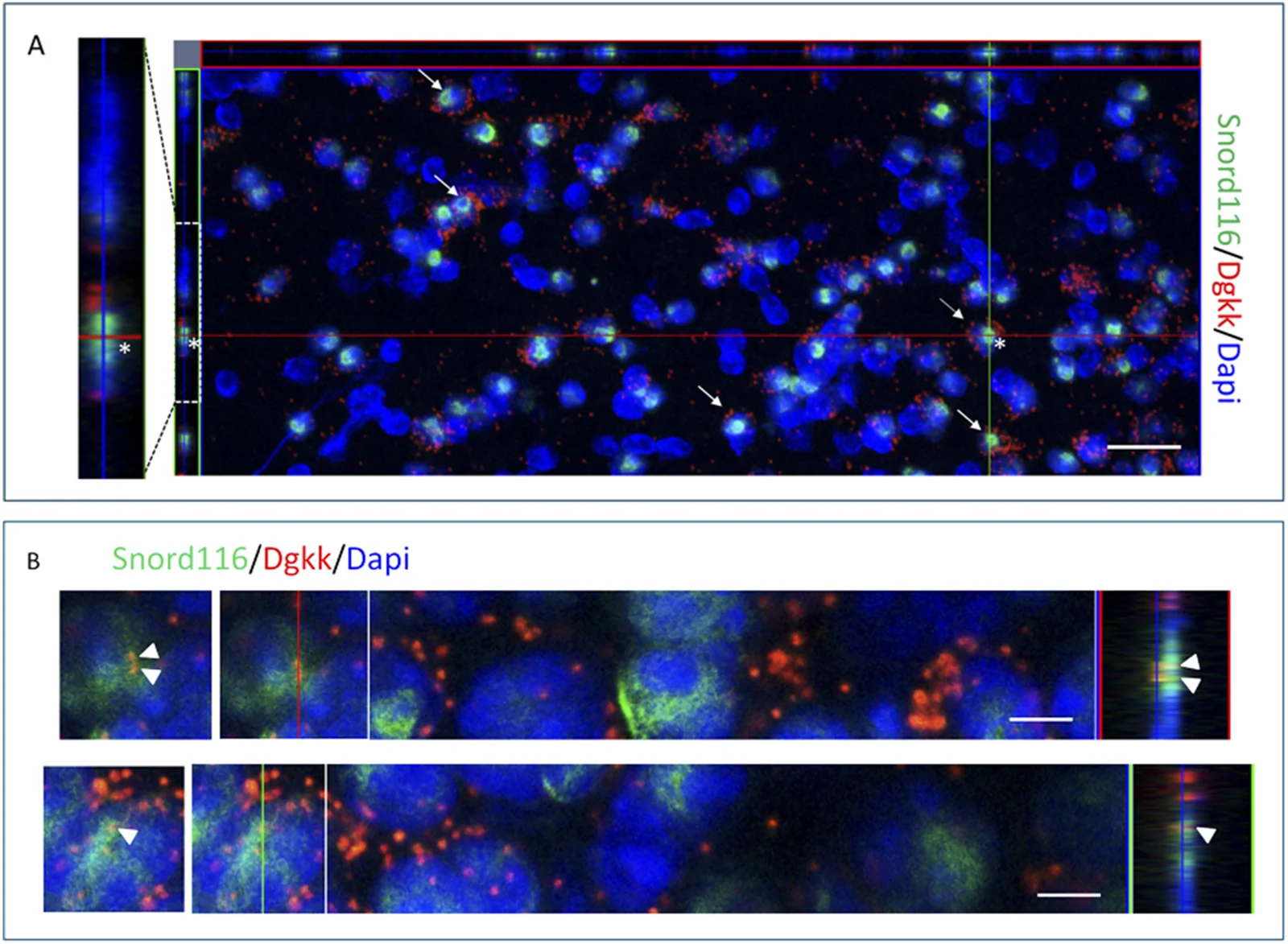
*Dgkk* is co-expressed and colocalized with *Snord116* in the nucleus of hypothalamic neurons. **(A)** Confocal images of the staining of *Snord116* (green) and *Dgkk* (red) and nuclei (blue) in the lateral hypothalamic nucleus with a Z-stack. Arrows indicate neurons positive for both *Snord116* and *Dgkk* staining. The left panel shows the enlarged Z-stack image in the white box that shows the relative positions of *Snord116* and *Dgkk* staining in the indicated neuron (white asterisk). **(B)** Confocal images of the staining of *Snord116* (green) and *Dgkk* (red) and nuclei (blue) in the arcuate nucleus (ARC) with a Z-stack. Two neurons were shown (upper and lower panels). Arrowheads indicate colocalizations of *Dgkk* with *Snord116* in the nuclei in the 2D images (left) and the corresponding Z-stack images (right). Scale bars are 20 μm in (A) and 10 μm in (B), respectively. RNAscope was performed on brain sections from 9-wk-old male C57BL/6J mice.

DGKK is one of the 10 known DGKs, which convert *sn*-1,2-DAG to PA. Although the physiological functions of DGKK are not well understood, several lines of evidence suggest that DGKK may be involved in the growth and maintenance of dendritic spines in the brain (Imai et al, 2005; Tabet et al, 2016a). A search of the GTEx and NCBI expression databases suggests that in the adult human brain, the expression of *DGKK* is one of the lowest among all DGK genes and is limited mainly to the striatum and hypothalamus. To explore *DGKK* expression profiles in a range of organs, developmental stages, and species, we searched the EVO-devo mammalian organs database generated in the Kaessmann Lab. Although a *DGKK* expression profile is not available in the human database, *Dgkk* expression in the brain is strongly developmentally regulated in rodents and Macaques, high during the later embryonic and early postnatal stages, decreasing precipitously to much lower levels throughout the rest of life stages (https://apps.kaessmannlab.org/evodevoapp/) (Cardoso-Moreira et al, 2019). We examined the expression patterns of *Snord116* and *Dgkk* in the adult mouse brain using a modified RNAscope technique. Because *Snord116* is highly expressed in the adult mouse brain, we were able to perform double staining of *Snord116* and *Dgkk* using RNAscope-based reagents. The *Snord116* and *Dgkk* probes were expected to target no other genes in the mouse genome except *Snord116* and *Dgkk*, respectively, and their specificities were confirmed by RNAscope analysis using WT and *Snord116* KO brain sections or by cross-referencing RNAscope results with qRT–PCR results on *Dgkk* expression patterns in various brain regions (Fig S8A and B). We found that *Snord116* was widely expressed in all brain regions examined, excluding cells of the ventricular linings and the blood–brain barrier, consistent with an enrichment in neuronal populations. In contrast, *Dgkk* mRNA expression in the adult mouse brain was highly restricted. In the hypothalamus, high levels of *Dgkk* expression were observed in the arcuate nucleus but not in the adjacent VMH (Fig 8A). High levels of *Dgkk* expression were also observed in the paraventricular and lateral nuclei of the hypothalamus, the dorsal raphe nucleus, and lateral parabrachial nucleus (Fig 8B–E). These brain regions have been repeatedly implicated in the regulation of feeding behavior. The cuneiform nucleus, which is located adjacent to the lateral parabrachial nucleus and is involved in movement, pain modulation, and cardiovascular regulation (Mohebbati et al, 2021), also expresses high levels of *Dgkk* (Fig 8E). Lower levels of *Dgkk* expression were found in the central amygdala, habenula, and medial nucleus of solitary tract (Fig S9A–C). A summary of *Snord116* and *Dgkk* expression in all brain regions examined is shown in Table 1. At the cellular and subcellular levels, *Dgkk* appeared to be mostly co-expressed with *Snord116*; *Snord116* staining was detected in the nucleus, whereas most of *Dgkk* staining appeared in the perinuclear region of the cytoplasm (Fig 9A). A small fraction of *Dgkk* staining was colocalized with *Snord116* in the nucleus (Fig 9B), suggesting that the nucleus is likely the location where these two molecules physically interact. The co-expression of *Dgkk* with *Snord116* in the key regions of the mouse brain known to be involved in regulating energy homeostasis, circadian rhythm, and motivation/reward, which are also dysregulated in PWS patients, supports an inference that the dysregulation of *DGKK* expression associated with *SNORD116* deficiency may contribute to the pathogenesis of PWS.

**Table 1.** *Dgkk* mRNA and *Snord116* expression in brain regions implicated in the regulation of energy balance, feeding behavior, and circadian rhythm.

Brain region	Distance to bregma (mm)	Relative *Dgkk* mRNA signal strength	*Snord116* expression
Nucleus accumbens, shell	1.4	**+**	yes
Anterior cingulate area	−0.83	**-**	yes
Paraventricular nucleus	−0.83	**+++**	yes
Anterior hypothalamic nucleus	−0.83	**++**	yes
Arcuate nucleus	−1.43	**+++**	yes
Ventromedial hypothalamic nucleus	−1.43	**-**	yes
Dorsomedial hypothalamic nucleus	−1.43	**++**	yes
Lateral hypothalamus	−1.43	**+++**	yes
Central amygdaloid nucleus	−1.43	**++**	yes
Medial amygdaloid nucleus	−1.43	**+**	yes
Central medial thalamic nucleus	−1.43	**++**	yes
Paraventricular thalamic nucleus	−1.43	**+**	yes
Medial/lateral habenular nucleus	−1.43	**+**	yes
Ventral tegmental area	−3.07	**++**	yes
Deep gray layer of superior colliculus	−3.07	**+**	yes
Substantia nigra, compact part	−3.07	**+**	yes
Lobule 2 of the cerebellar vermis	−5.19	**-**	yes
Dorsal raphe nucleus, caudal	−5.19	**++++**	yes
Lateral parabrachial nucleus	−5.19	**+++**	yes
Cuneiform nucleus	−5.19	**+++**	yes
Lateral (dentate) cerebellar nucleus	−6.11	**-**	yes
Lobule 4/5 of the cerebellar vermis	−6.11	**-**	yes
Nucleus of the solitary tract	−6.83	**++**	yes
Raphe pallidus nucleus	−6.83	**+**	yes

Expressions of *Snord116* and *Dgkk* were detected using the RNAscope assay following the protocol recommended by the manufacturer. RNAscope experiments were performed on 20-μm coronal sections from 9-wk-old male C57BL/6J mice containing the indicated brain regions. Fluorescent images were taken using a Zeiss confocal microscope under the same exposure condition, and the relative DGKK expression levels were estimated by visual examination based on the intensity and density of fluorescence staining in the specific brain regions. Signal strength is indicated from + to ++++, decreasing in the order of ++++ > +++ > ++ > +. However, ++++ should not be taken as an indication that it was two times stronger than ++ or four times stronger than +.

### *Snord116* deficiency attenuates PKC and ERK signaling associated with the activation of group 1 metabotropic glutamate receptors

**Figure S10.**
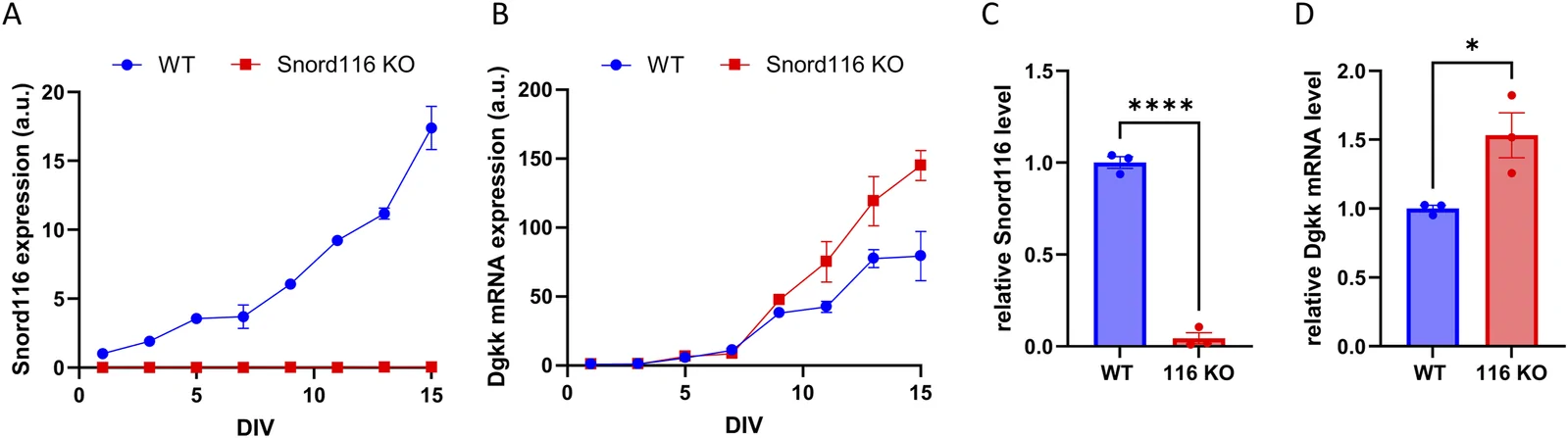
Expression of *Snord116* and *Dgkk* in MCNs isolated from newborn pups (MCNs). **(A, B)** Time course of *Snord116* (A) and *Dgkk* (B) expression in MCNs that have been cultured in vitro (days in vitro, DIV) for 1, 3, 5, 7, 9, 11, 13, and 15 d. **(C, D)** *Snord116* (C) and *Dgkk* (D) expression in the WT and *Snord116* KO (116 KO) DIV14 MCNs used for the mGluR1/5 signaling study shown in Fig 10. * and **** indicate *P* < 0.05 and 0.0001 respectively, *Snord116 *KO versus WT by two-tailed *t* test.

**Figure 10.**
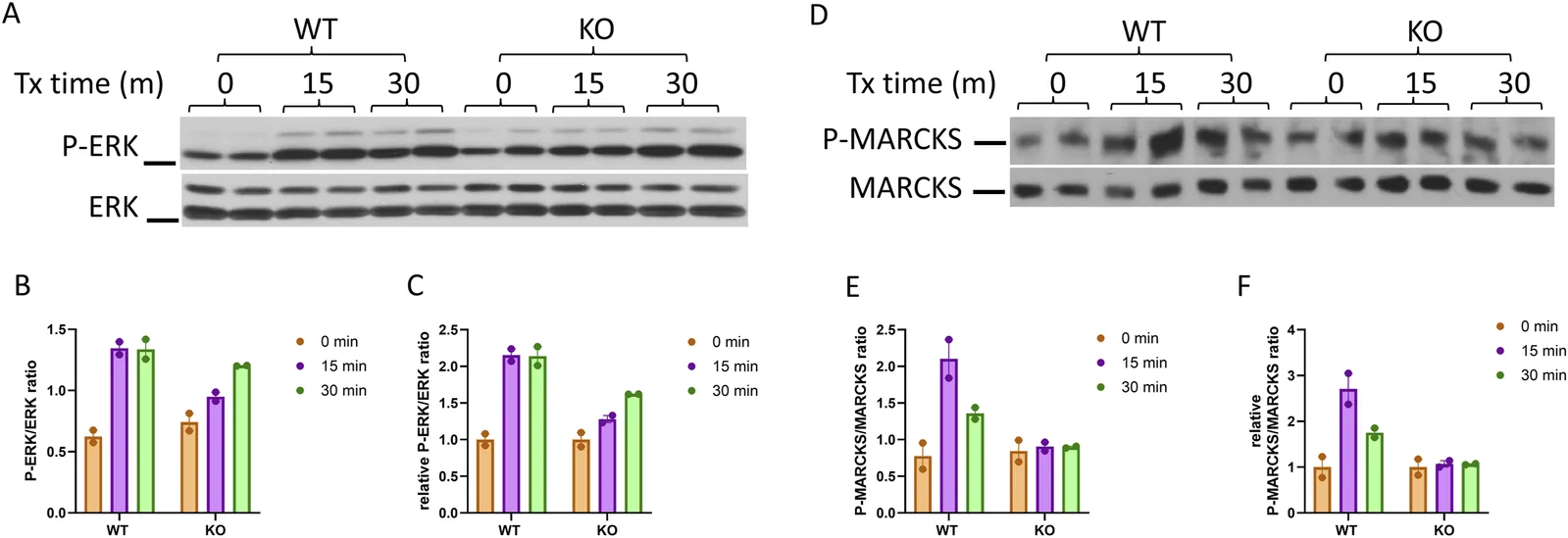
Attenuated ERK and PKC signaling in response to group 1 metabotropic glutamate receptor activation in *Snord116* KO MCNs. **(A)** DIV14 MCNs isolated from newborn pups were treated with 100 μM DHPG, a selective group I metabotropic glutamate receptor agonist for 0, 15, and 30 min. Phospho-ERK1/2 (P-ERK) and total ERK1/2 (total ERK) were detected by Western blotting using rabbit anti-P-ERK and mouse anti-total ERK antibodies. The horizontal bars indicate the migratory position of a 37-kD protein marker. **(B)** Amounts of P-ERK and total ERK in A were quantified using densitometry, and the activation level of ERK is expressed as the ratio of P-ERK to total ERK. **(C)** DHPG-induced ERK activation is shown as the relative P-ERK-to-total ERK ratio normalized to the respective basal P-ERK-to-total ERK ratio (time 0) in the WT and *Snord116* KO MCNs. Two-way ANOVA showed that the effect of DHPG treatment and the interaction between genotype and DHPG treatment were statistically significant, both *P* < 0.01, and Dunnett’s multiple comparisons test showed that DHPG-induced ERK signaling is significant at both 15 and 30 min in the WT MCNs (*padj* < 0.01 and 0.05, respectively) but not significant at either 15 or 30 min in *Snord116* KO MCNs (*padj* = 0.15 and 0.14, respectively). **(D)** Phospho-MARCKS and total MARCKS in the same samples shown in A were detected by Western blotting using rabbit anti-P-MARCKS and mouse anti-total MARCKS antibodies. The horizontal bars indicate the migratory position of a 75-kD protein marker. **(E)** Amounts of P-MARCKS and total MARCKS in (D) were quantified using densitometry, and the activation level of PKC is expressed as the ratio of P-MARCKS to total MARCKS. **(F)** DHPG-induced PKC activation is shown as the relative P-MARCKS-to-total MARCKS ratio normalized to the respective basal P-MARCKS-to-total MARCKS ratio (time 0) in the WT and *Snord116* KO MCNs. By two-way ANOVA, the effect of DHPG treatment and the interaction between genotype and DHPG treatment were statistically significant, both *P* < 0.05, and the genotype effect was approaching statistical significance, *P* = 0.0501. Dunnett’s multiple comparisons test showed that DHPG-induced PKC activation is significant at 15 min (*padj* < 0.01) and approaching significance at 30 min (*padj* = 0,08) in the WT MCNs but not significant at either 15 or 30 min in *Snord116* KO MCNs (*padj* = 0.94 and 0.95). The experiments were repeated three times, and the results of a typical experiment with two technical replicas are shown.

DAG, a substrate of DGKK, is an important downstream effector of Gq-coupled receptors, which include group 1 metabotropic glutamate receptors (mGluR1/5) and many other neuropeptide/neurotransmitter receptors, such as those for orexin A and melanin-concentrating hormone. The elevated plasma membrane DAG level, as a result of activation of these receptors and subsequently phospholipase C, activates ERK through several downstream effectors, including PKC and RASGRP (Pierret et al, 2000; Brose & Rosenmund, 2002; Jones et al, 2002; Chen et al, 2017). ERK signaling activities in neurons play a critical role in regulating dendritic spine formation and stability, and therefore synaptic plasticity and signaling (Pierret et al, 2000; Balazs, 2006; Leal et al, 2014; Vithayathil et al, 2018; Bagni & Zukin, 2019). Elevated *Dgkk* expression in *Snord116*-deficient neurons could dysregulate signaling activities mediated by these receptors and their related cellular functions. We examined the effects of *Snord116* deficiency on ERK and PKC signaling pathways downstream of mGluR1/5 in mouse cortical neurons (MCNs) isolated from newborn pups that had been cultured in vitro for 14 d (DIV14). *Snord116* and *Dgkk* expression levels were very low in the freshly isolated MCNs and increased as the neurons matured in vitro (Fig S10A and B). Around DIV11, *Dgkk* expression started to diverge between *Snord116* KO and WT neurons and remained elevated in *Snord116* KO neurons relative to WT controls between DIV11 and DIV15 (Fig S10B). Lack of *Snord11*6 expression and elevated *Dgkk* expression in DIV14 *Snord116* KO neurons used for the mGluR1/5 stimulation study were also confirmed (Fig S10C and D). At DIV14, basal levels of ERK activity were slightly (19%) higher in *Snord116* KO MCNs relative to WT MCNs. Treatment of these MCNs with (*S*)-3,5-dihydroxyphenylglycine (DHPG), a selective mGluR1/5 agonist, led to robust activation of ERK in the WT MCNs (Fig 10A-C). However, DHPG-induced ERK activation was significantly attenuated in *Snord116* KO MCNs (Fig 10A–C). In addition, the DHPG treatment rapidly and robustly increased MARCKS phosphorylation, a substrate of PKC. Again, the treatment failed to induce detectable phosphorylation of MARCKS in *Snord116* KO MCNs (Fig 10D–F). These results suggest that *Snord116* deficiency is associated with impaired mGluR1/5-DAG signaling and that elevated *Dgkk* expression may account for the decreased signaling activity in *Snord116* KO neurons.

**Figure 11.**
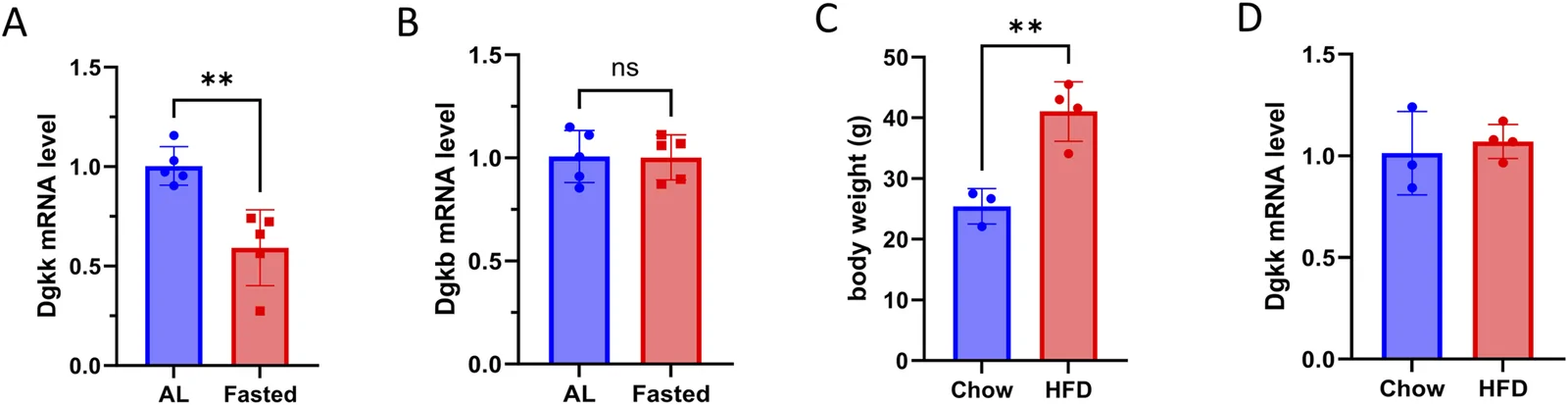
Effects of fasting and diet-induced obesity on *Dgkk* expression in adult mouse hypothalamus. **(A, B)** Transcript levels of *Dgkk* (A) and *Dgkb* (B) in the hypothalamus of 9-wk-old male C57BL/6J mice that had been subjected to a 16-h fasting (fasted) or fed ad libitum (AL). **(C, D)** Body weights (C) and hypothalamic *Dgkk* transcript levels (D) of 20-wk-old male C57BL/6J mice that had been subjected HFD feeding (60% fat diet) or maintained on the regular chow diet (Chow) for 12 wk starting at 8 wk of age. *Dgkk* mRNA expression was normalized to the geomean of two housekeeping genes, *Tbp* and *Ppia*, and expressed as relative levels in an arbitrary unit. Data are shown as the mean ± SEM. ** denotes *P* < 0.01, fasted versus AL or chow versus HFD by a two-tailed *t* test. HFD, high-fat diet.

To explore the potential role of *Dgkk* in the regulation of energy homeostasis, we investigated how fasting and obesity affect *Dgkk* mRNA expression in mouse hypothalamus. In 9-wk-old male mice, 16-h fasting decreased hypothalamic *Dgkk* mRNA level by 46% compared with the ad libitum controls (AL) (Fig 11A). In contrast, the transcript level of *Dgkb*, another DGK isoenzyme, was not significantly altered (Fig 11B). Diet-induced obesity in animals fed a high-fat diet had no significant effect on hypothalamic *Dgkk* transcript levels (Fig 11C and D). These results suggest that hypothalamic *Dgkk* expression is sensitive to alterations in the metabolic status, specifically acute energy deficits. Previous studies have shown that fasting leads to the activation of several MAP kinases, including ERK and P38, in the mouse arcuate nucleus (Morikawa et al, 2004). Decreased *Dgkk* expression in the fasted state may contribute to the activation of MAPK and physiological responses to the acute energy deficits.

**Figure 12.**
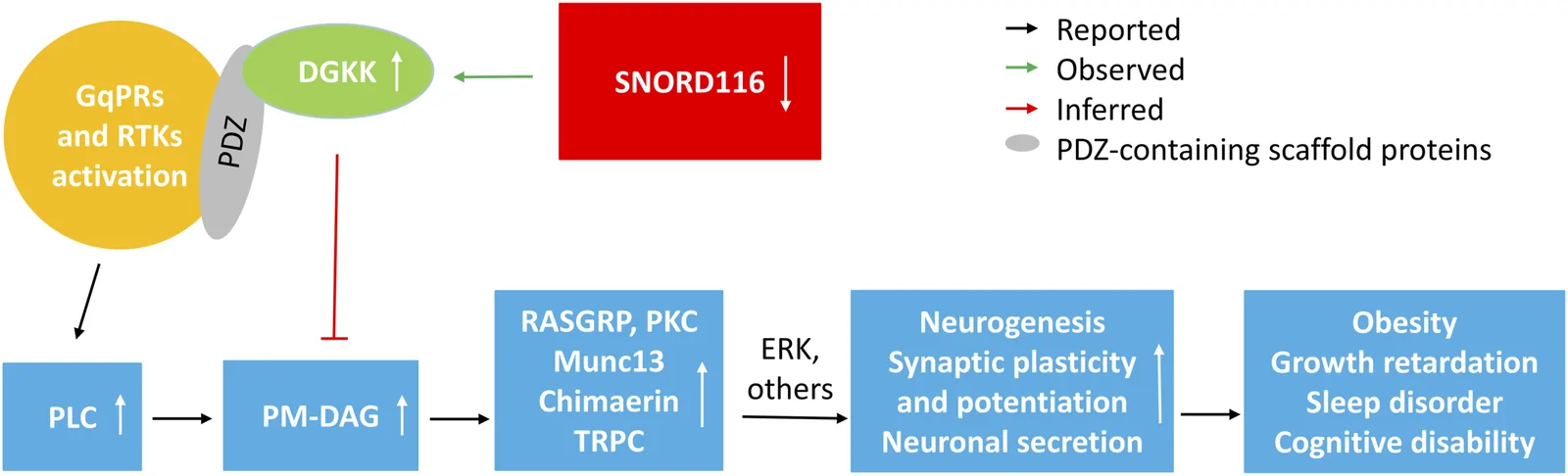
Working model: molecular mechanisms underlying PWS pathophysiology caused by *SNORD116* deficiency and increased *DGKK* expression. *SNORD116* deficiency leads to elevated DGKK expression on the plasma membrane, which is associated with Gq-coupled receptors (GqPRs) and/or receptor tyrosine kinases (RTKs) possibly via PDZ-containing scaffold proteins (PDZ). The activation of these receptors leads to the activation of phospholipase C (PLC), which hydrolyzes membrane phosphoinositides and increases the local concentration of PM-DAG, a second messenger and substrate of DGKK. Elevated DGKK expression associated with *SNORD116* deficiency could attenuate or prematurely terminate the signaling activity of local PM-DAG. PM-DAG is an effector that regulates the activities of multiple proteins, including protein kinase C (PKC), RAS guanyl nucleotide-releasing protein (RASGRP), transient receptor potential channel (TRPC), Munc13, and chimerin, which in turn regulate the activities of extracellular signal-regulated kinase (ERK) and other proteins or protein complexes and affect neuronal functions, such as neurogenesis, synaptic plasticity and potentiation, and the release of neurotransmitters and neuropeptides. Dysregulation and impairment in these neuronal activities/functions may contribute to aspects of PWS pathophysiology, such as obesity, growth deficiency, sleep disorder, and cognitive disability. As shown in the figure, the black arrows indicate processes/relationships that have been previously reported and observed. The green allow indicates the result observed in the current study, and the red T line indicates the inferred suppressive effect of the elevated DGKK expression on PM-DAG signaling based on the results of the current study. PM-DAG, plasma membrane–associated diacylglycerol.

In summary, we have shown that *SNORD116* deficiency (a molecular hallmark of PWS) leads to increased *DGKK* mRNA and protein expression. In *Snord116*-deficient mouse neurons, increased *Dgkk* expression is associated with attenuated DAG-mediated signaling downstream of the activation of mGluR1/5, Gq-coupled receptors, including PKC and ERK. *SNORD116* and *FBL* also promote 2′-O-methylation of U1483 in human *DGKK* mRNA, which requires the predicted RNA:RNA interaction between *SNORD116* and *DGKK*. However, increased *DGKK* expression associated with *SNORD116* deficiency appears to be independent of 2′-O-methylation of U1483 or RNA stability in SK-N-FI human neuroblastoma cells. We have further shown that *Dgkk* is expressed in key mouse brain regions that regulate energy balance, feeding behavior, and neuroendocrine functions and that hypothalamic *Dgkk* expression is sensitive to acute energy deficit. DGKK, PKC, and ERK have all been previously implicated in synaptic plasticity, which in turn has been implicated in the creation and response of neural circuits essential for brain functions, such as energy homeostasis, learning ability, skeletal motor activities, and social behaviors (Bouret et al, 2004; Krashes et al, 2014; Tabet et al, 2016a; Wang et al, 2019; Grzelka et al, 2023). Thus, dysregulation of *DGKK* expression because of *SNORD116* deficiency could contribute to the pathogenesis of PWS. Fig 12 depicts a working model for the roles of *SNORD116* and *DGKK* in the regulation of DAG-mediated signaling and their connection to the pathogenesis of PWS.

## Discussion

### Cellular pathways affected by *Snord116* deficiency in mice

We conducted transcriptomic profiling of neonatal hypothalami of two independent mouse models of *Snord116* deficiency to capture the effects of *Snord116* deficiency on the developing hypothalamus (Wang et al, 2012; Maggi et al, 2014; Burbridge et al, 2016). The expression of E2F target genes, which are involved in regulating cell cycle progression, was negatively enriched in *Snord116*-deficient mice, suggesting that *Snord116* deficiency may decrease cell proliferation and/or accelerate neuronal differentiation. Our results were consistent with a recent report by Helwak et al (2024), which showed that *Snord116*-deficient neurons derived from LUHMES human neural progenitor cells undergo a slightly accelerated maturation process compared with WT neurons, supporting the notion that *Snord116* may influence neuronal differentiation processes.

Genes involved in cholesterol synthesis were also markedly down-regulated. The brain is one of the most cholesterol-rich organs in vertebrates. In the developing mouse brain after the establishment of the blood–brain barrier around E15, the CNS synthesizes most of its cholesterol locally to enable the rapid expansion of brain mass (Quan et al, 2003). Cholesterol plays a critical role in regulating cellular patterning and synaptogenesis in addition to its role as a major component of the myelin sheath and plasma membranes (Lu et al, 2022). Congenital deficiency of 7-dehydrocholesterol reductase (DHCR7), an enzyme in one of the final steps of cholesterol biosynthesis, is the cause of Smith–Lemli–Opitz syndrome (SLOS) (Nowaczyk & Irons, 2012). In SLOS, some of these protean effects may be conveyed via impaired function of cholesterol-deficient primary cilia, such as Hedgehog signaling (Huang et al, 2016). *Dhcr7* expression was also reduced in P5 hypothalami of both IC del and *Snord116* KO mice along with several other genes in the cholesterol synthesis pathway. Some of the neurodevelopmental/behavioral abnormalities associated with SLOS, such as intellectual disability, sleep-cycle disturbance, self-injurious behavior, and autism spectrum disorder, have also been observed in PWS. Hypotonia, feeding difficulty, and growth retardation are commonly found in infants with SLOS and PWS (Nowaczyk & Irons, 2012). As expected, the genes most affected in the E2F and cholesterol synthesis pathways overlap between IC del and *Snord116* KO mice. Impaired cholesterol synthesis because of *Dhcr7* hypomorphic mutation leads to abnormal patterns of neuroprogenitor proliferation in the mutant forebrain and accelerated cortical differentiation in primary neuron culture from mutant mice (Driver et al, 2016), suggesting that decreased cholesterol synthesis and/or the accumulation of DHCR7 substrates may be causally related to the decreased proliferating cell population in the P5 hypothalamus of *Snord116*-deficient mice.

The Slits-Robos synthesis and signaling pathways were both positively enriched in the two *Snord116*-deficient models relative to their WT controls. Slit-Robo signaling mediates axon repulsion in the developing brain, including the hypothalamus (Blockus & Chédotal, 2014; Gonda et al, 2020; Romanov et al, 2020). The pathway has also been implicated in regulating neuron migration and positioning (Kim et al, 2019; Gonda et al, 2020). Loss of Slit-Robo signaling impaired both the production and positioning of periventricular dopamine neurons in the mouse hypothalamus (Romanov et al, 2020).

### *DGKK* as a direct target of *SNORD116*

Convergent lines of evidence implicate *DGKK* as a bona fide target of *SNORD116* in both humans and mice. *Dgkk* expression was increased in P5 hypothalami of both IC del and *Snord116* KO mice. The elevated hypothalamic *Dgkk* expression persists into adulthood in *Snord116* KO mice. In human iPSCs, PWS T1 del is associated with elevated *DGKK* mRNA and protein levels relative to an isogenic nondeleted control. In human neuroblastoma cells, ASO-mediated *SNORD116* KD increases *DGKK* mRNA and protein levels, although no significant changes in *DGKK* mRNA stability were observed. Double staining of *Dgkk* and *Snord116* in the adult mouse brain using RNAscope revealed that *Dgkk* is co-expressed with *Snord116* in selected groups of neurons and that a small fraction of *Dgkk* mRNA is colocalized with *Snord116* in the nuclei of these cells. A well-recognized function of SNORDs is to facilitate Nm of small RNAs by guiding the 2′-O-methylation RNP complex to RNA targets through sequence complementation. In this study, we have shown that *DGKK* is 2′-O-methylated at U1483, which is in the mRNA region predicted to interact with human group I *SNORD116*, and that 2′-O-methylation of U1483 is *SNORD116*-dependent in human neuroblastoma SK-N-FI cells and in iPSCs. Compensatory mutation analysis confirmed that the predicted sequence complementarity between the mouse *Snord116* (and group 1 human *SNORD116*) and *DGKK* is required for *Snord116*-dependent 2′-O-methylation of *DGKK* at U1483. Ye et al have shown that in stem cell–derived human neurons, *DGKK* mRNA is 2′-O-methylated at C1484 in a *SNORD116*-dependent manner (Ye et al, 2025 *Preprint*, PMID: 41446073), which is predicted to be mediated by human group II *SNORD116* elements. It is possible that human groups I and II *SNORD116* may guide the 2′-O-methylation of *DGKK* mRNA at U1483 and/or C1484, respectively. The Nm-VAQ and RTL-PCR assays used in our study and the RibOxi-seq method used in the study by Ye et al are not expected to be able to distinguish whether either or both U1483 and C1484 are 2′-O-methylated based on the principles of these assays. It might be possible to further examine these possibilities, which would not alter our conclusions regarding the proposed physical interactions between *SNORD116*s and *DGKK*, using additional methods and techniques. Notwithstanding the technical limitations, the observations that *SNORD116* mediates 2′-O-methylation of *DGKK* mRNA in different biological systems with different experimental approaches further support the inference that *DGKK* is a bona fide *SNORD116* target. However, it remains unclear whether the predicted interaction based on the sequence complementation between *SNORD116* and *DGKK* and/or the methylation of U1483 are necessarily the underlying mechanisms for the increased *DGKK* expression associated with *SNORD116* deficiency.

Gene expression profiling has been used to identify *SNORD116* molecular targets in various model systems, including postmortem hypothalami from PWS patients (Bochukova et al, 2018), fibroblast lines from PWS patients (Burnett et al, 2017), HEK293 cells overexpressing *Snord116* (Falaleeva et al, 2015), and neurons derived from WT and *SNORD116*-deficient human ES cells or human LUHMES neuronal progenitor cells (Gilmore et al, 2024; Helwak et al, 2024). The potential *SNORD116* target genes identified in these studies showed little overlap with each other or with the shared hypothalamic DEGs between IC del and *Snord116* KO mouse models identified in the current study, including *Dgkk*. The lack of such overlap may have multiple causes, including differences in the model systems, genetic background (between samples within a study and between different studies), and differentiation stage of the neurons analyzed. *DGKK* has been implicated as a potential *SNORD116* target in two isogenic cellular models (Baldini et al, 2022; Ye et al, 2025 *Preprint*). Consistent with these reports, our physical and physiological data obtained from isogenic animal and cellular models provide independent and substantial evidence that *DGKK* is a bona fide functional target of *SNORD116*.

We also found that *FBL* KD decreased 2′-O-methylation of *DGKK* U1483 in SK-N-FI cells. The concordant decreases in the level of Um1483 in *SNORD116* KD and *FBL* KD SK-N-FI cells suggest that both *SNORD116* and *FBL* promote 2′-O-methylation of U1483 in human *DGKK* mRNA. U1483 constitutes the first base of the Ser446 codon UCC of human *DGKK* mRNA. The modification could potentially affect *DGKK* mRNA and protein levels via multiple mechanisms, including changes in RNA secondary structure and translation efficiency (Elliott et al, 2019; Abou Assi et al, 2020; Ojha et al, 2020). However, the discordant effects of *SNORD116* KD *(increase)* and *FBL* KD *(decrease)* on *DGKK* mRNA levels suggest that 2′-O-methylation of U1483 is unlikely to be the molecular mechanism underlying the elevated *DGKK* mRNA and protein levels in *SNORD116-*deficient cells. Both canonical and noncanonical snoRNPs exist in cells, and the same snoRNAs could be assembled into different protein complexes for distinct functions (Kass et al, 1990; Dragon et al, 2002; Schubert et al, 2012; Youssef et al, 2015; Falaleeva et al, 2016; Huang et al, 2017; Ojha et al, 2020; Han et al, 2022). For example, *SNORD27* is involved in 2′-O-methylation of A27 in 18S rRNA and also regulates alternative splicing of the transcription factor E2F7 pre-mRNA through direct RNA–RNA interaction without methylating the RNA (Falaleeva et al, 2016). U3 (*SNORD3*), a member of the box C/D family of snoRNA, is a central component of a large processing complex called the small subunit processome, which is required for nucleolar processing of pre-18S ribosomal RNA, but is not involved in modifying 18S rRNA (Kass et al, 1990; Dragon et al, 2002). *Snord50A*, a U/A-rich box C/D snoRNA, inhibits mRNA 3′ processing by blocking the Fip1-poly(A) site interaction. *Snord50A* depletion alters the Fip1-RNA interaction landscape and changes the alternative polyadenylation profiles and/or transcript levels of a large number of genes (Huang et al, 2017). SNORDs are also known to be associated with chromatin and to affect accessibility of the associated DNA (Schubert et al, 2012; Han et al, 2022). Thus, it is possible that *SNORD116* may regulate *DGKK* expression via multiple molecular mechanisms. Alternatively, despite the absence of evidence that *FBL* KD induced cellular stress/toxicity in SK-N-FI cells, the possibility that cellular stress/toxicity associated with *FBL* KD may underlie the discordant effects of *SNORD116* KD and *FBL* KD on *DGKK* mRNA levels in these cells cannot be ruled out.

### Implications of the increased *DGKK* expression in the pathogenesis of PWS

**Figure S11.**
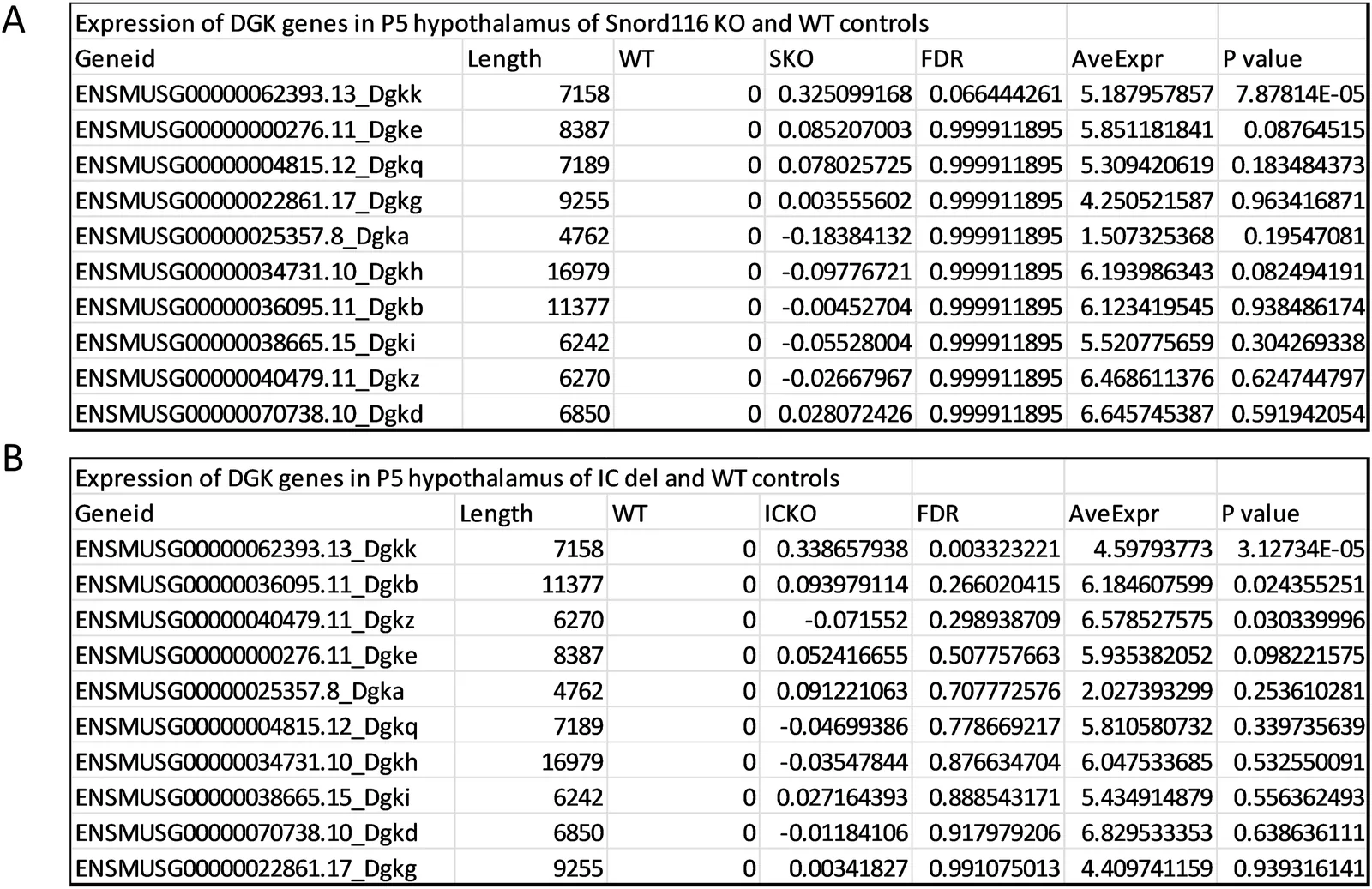
Relative expression levels of all known DGKs in the P5 hypothalamus of *Snord116*-deficient mouse models relative to their WT controls. **(A)** Log2FC in the expression of DGKs in the P5 hypothalamus of *Snord116* KO mice (3 males and 2 females) relative to their sex-matched WT controls. A total of 20 DEGs were detected in the P5 hypothalamus between *Snord116* and WT controls (FDR < 0.1). **(B)** Log2FC in the expression of DGKs in the P5 hypothalamus of IC del mice (5 males and 5 females) relative to their WT controls. A total of 700 DEGs were detected in the P5 hypothalamus between IC del and WT controls (FDR < 0.1). *Dgkk* is the only one among all known DGKs differentially expressed in *Snord116* KO and IC del models (FDR < 0.1). Log2FC, Log2 fold change.

DGKs catalyze the phosphorylation of DAG to PA. Both DAG and PA are lipid signaling molecules that regulate numerous downstream signaling pathways and influence many cellular functions, including neurogenesis, synaptogenesis, and metabolism (Blouet et al, 2008; Barber & Raben, 2020; Sakane et al, 2020). DGKK is one of the 10 known DGKs (Imai et al, 2005; Barber & Raben, 2020; Sakane et al, 2020). These DGKs differ in their tissue and subcellular distributions, substrate selectivity, and interacting partners, which could influence or even determine the specific physiological function of each DGK in each tissue/cell (Barber & Raben, 2020; Sakane et al, 2020). DGKK is expressed primarily in CNS neurons, and the protein is localized primarily on the plasma membrane (Imai et al, 2005; Tabet et al, 2016a). sn-1,2-DAG, a second messenger derived mainly from the hydrolysis of membrane phosphoinositides as a result of activation of phospholipase Cs, acts downstream of Gq-coupled receptors and receptor tyrosine kinases. Thus, DGKK has the potential to modulate signaling activities of these receptors by decreasing the second messenger DAG levels. Indeed, we found that increased DGKK expression is associated with attenuated ERK and PKC signaling, both regulated by sn-1,2-DAG, in *Snord116* KO mouse neurons after the activation of mGluR1/5. Although we cannot rule out the possibility that elevated expression of other DGKs may contribute to the inhibitory effects on ERK and PKC signaling in *Snord116* KO mouse neurons, only *Dgkk* expression appeared to be affected by *Snord116* deficiency in the P5 hypothalamus of both *Snord116* KO and IC del mice (Fig S11A and B). ERK and PKC are important regulators of synaptogenesis and dendritic spinogenesis (Pierret et al, 2000; Balazs, 2006; Leal et al, 2014; Vithayathil et al, 2018; Bagni & Zukin, 2019). Decreased DGKK expression in cortical neurons because of the lack of Fragile X mental retardation protein destabilizes dendritic spines (Tabet et al, 2016a, 2016b). It is possible that DGKK may interact with mGluR1/5 via its PDZ binding domain (SQL) located at the C terminus of DGKK (Imai et al, 2005). mGluR1/5 are known to be associated with Homer and other PDZ-containing scaffolding molecules, which are required for efficient activation of these receptors (Brakeman et al, 1997; Hwang et al, 2005). In addition to modulating PKC and ERK signaling downstream of mGluR1/5, DGKK may modulate other cellular processes, including neuronal secretion and ion channel activity by interacting with other receptors and DAG-binding molecules through the putative PDZ binding domain and other protein/lipid-interacting domains, such as the pleckstrin homology domain (Bauer et al, 2007; Kume et al, 2016; Michelassi et al, 2017; Liu et al, 2020), as shown schematically in Fig 12. Effects of explicit normalization of DGKK expression in cellular and animal models on PKC and ERK activations downstream of mGluR1/5 and on specific PWS phenotypes are needed to confirm the role of DGKK in regulating mGluR1/5 signaling and pathophysiology of PWS.

One striking finding is that in the adult mouse brain *Dgkk* expression is restricted mainly to the regions that are part of the mesolimbic and autonomic systems implicated in the regulation of energy balance, feeding behavior, and sleep patterns. Consistent with our RNAscope results in mouse brain, appreciable levels of *DGKK* expression are found mainly in the same human hypothalamic nuclei that are part of the neural circuitry involved in the regulation of energy balance and food intake, such as ARC, DMH, PVH, and LH (Broad Single Cell Portal) (Campbell et al, 2017; Herb et al, 2023; Tang et al, 2023). Relatively high levels of *DGKK* expression are found in human VMH but not in mouse VMH, suggesting an evolutionary divergence in gene regulation. Further analysis of single-cell RNAseq data from the mouse arcuate nucleus and the median eminence shows that *Dgkk* is expressed almost exclusively in neurons and that the number/fraction of *Dgkk*-positive cells, as well as *Dgkk* expression levels, is highly variable among different neuronal clusters. High numbers/fractions of *Dgkk*-positive neurons are found in the neuronal clusters bearing molecular signatures of *Agrp/Gm8773*, *Pomc/Anxa2*, *Ghrh*, and *Htr36*; very low frequency of *Dgkk*-positive neurons are found in those bearing the signatures of *Nr5a1/Bdnf* and *Rgs16/Vip* (Broad Single Cell Portal) (Campbell et al, 2017). In contrast, the expression of *Dgkd*, another member of the DGK family, is found in both neuronal and nonneuronal cells of the arcuate nucleus and median eminence, underlining the divergence in physiological function of different DGKs (Broad Single Cell Portal) (Campbell et al, 2017).

*DGKK* likely also plays an important role in the development of CNS. In mice, *Dgkk e*xpression in the forebrain and cerebellum peaks in the late embryonic and early postnatal stages and then drops to a much lower but constant level from P28 onward (Cardoso-Moreira et al, 2019). However, very little impact on body weight, length, or metabolic characteristics was observed in a congenital *Dgkk* KO mouse line (mouse phenotyping consortium). Several other DGKs, including *Dgkb, Dgkg, and Dgkz,* are also expressed in the hypothalamus and have been implicated in regulating dendritic development and synaptic plasticity (Yakubchyk et al, 2005; Kim et al, 2009; Shirai et al, 2010; Hozumi et al, 2015, 2021). Thus, genetic compensation by other DGKs in the *Dgkk* KO mouse line cannot be ruled out. In the developing human hypothalamus (GW ≤ 25), single-cell sequencing data revealed that *DGKK* expression levels are very low (<2 CPM). The precise role of *DGKK* in human hypothalamic development remains unknown (Herb et al, 2023).

We have demonstrated that *Dgkk* expression is sensitive to energy homeostasis perturbations. Acute energy deficits, like fasting, markedly down-regulate *Dgkk* mRNA levels in the adult mouse hypothalamus. On the other hand, we did not detect significant changes in hypothalamic *Dgkk* mRNA expression in mice with high dietary fat-induced obesity relative to the chow-fed control mice. Previous studies have shown that fasting activates ERK in both arcuate and PVH in mice (Morikawa et al, 2004). It remains to be determined whether decreased *Dgkk* expression in the fasted state contributes (possibly via increased DAG) to the increased ERK activation. The role of *DGKK* in the development of hyperphagic obesity associated with PWS is unknown. Mouse models of PWS, including the two used in the current study, do not develop hyperphagic obesity that is characteristic of PWS individuals (Yang et al, 1998; Ding et al, 2008; Cassidy et al, 2012; Butler et al, 2019). The lack of hyperphagia and obesity phenotype in PWS mouse models may be due to divergence in the development of the feeding circuits (Herb et al, 2023), to runting in these mouse models (Yang et al, 1998; Ding et al, 2008; Cassidy et al, 2012; Butler et al, 2019), and to differences in the genetic organization of the PWS regions in human and mouse (Good & Kocher, 2017; Baldini et al, 2022; Fig 1). The mouse *Snord116* cluster encodes virtually identical *Snord116* elements, whereas human *SNORD116* cluster encodes S*NORD116* elements with diverse sequences (Fig 1), which may be expected to interact with molecular targets beyond those shared with mouse *Snord116* (Gilmore et al, 2024). It is also of interest to note that human DGKK contains a unique segment composed of ∼33 EPAP repeats (Glu48-Pro179) near the N terminus, which is absent in mouse DGKK (Imai et al, 2005). The role of this domain is unknown, but it could potentially mediate interactions with proteins that are specific to human DGKK and may account for some of the phenotypic differences between PWS patients and mouse models of PWS.

The current study has several limitations. The deletion of the *Snord116* locus in the *Snord116* KO mouse model used here results in *Snord116* deficiency, as well as deficiencies of several long noncoding RNAs in the region, including *116HG, SPAs, sno-lncRNAs*, and *Ipw*, whose 5’-end sequence overlaps with the 3’-end of the *Snord116* locus (Ding et al, 2008; Yin et al, 2012; Wu et al, 2016). We found that deletion of the *Snord116* cluster reduced *Ipw* expression (including the 3’-end of IPW) and *Snord64*, which is located outside of the deleted segment in *Snord116* KO mice (Ding et al, 2008). Thus, we cannot rule out the possibility that deficiencies of these lncRNAs and *Snord64* may contribute to part or all the physiological and molecular phenotypes observed in the current study. In addition, multiple genes are predicted to be the targets of *SNORD116* (Good & Kocher, 2017; Bochukova et al, 2018; Kocher et al, 2021; Baldini et al, 2022); several of them, including *DGKK*, have been shown to be affected by *SNORD116* KD in HeLa S3 cells (Baldini et al, 2022). We failed to detect differential expressions of several of these genes, including *Nhlh2*, *Nlgn3*, and *Rsbn1l*, in P5 hypothalami from IC del and *Snord116* KO mice. However, the possible contribution of these and other potential *SNORD116* targets to the physiological and molecular phenotype in *SNORD116*-deficient animals and cells cannot be ruled out.

In conclusion, we have identified several pathways and genes dysregulated in the developing hypothalamus of *Snord116*-deficient mice that could affect the proliferation, differentiation, and migration of neural progenitor cells. Our results provide new insights into the molecular function of *Snord116* and the underlying mechanisms by which *Snord116* regulates *Dgkk* expression and RNA modification, as well as the potential role of DGKK in regulating PKC and ERK activities downstream of the mGluR1/5-PLC-DAG signaling pathway. Implication of the elevated DGKK activity in PWS pathogenesis underlines the possibility of using DGK inhibitors (Nakano et al, 2014; Sakane et al, 2016; Noessner, 2017; Hayashi et al, 2019; Sabnis, 2025), as a potential pharmacologic intervention for PWS. The detailed mapping of *Dgkk* expression in the adult mouse brain could provide a road map for a mouse modeling system, which can be used to determine how deficiency of *Snord116* and its interactions with *Dgkk* in different mouse brain regions may contribute to specific PWS phenotypic traits such as hyperphagia, abnormal neuroendocrine function, cognitive deficits, and motor learning disability.

## Materials and Methods

### Cell lines, reagents, and chemicals

Plasmids encoding C-terminal FLAG-tagged human *DGKK* in pcDNA3.1^+^/C-DYK vector (pcDNA3.1^+^hDGKK/C-FLAG) and N-terminal FLAG-tagged mouse *Dgkk* cDNA in pcDNA3.1^+^/N-DYK vector (pcDNA3.1^+^mDGKK/N-FLAG) were purchased from GenScript. The plasmid encoding mouse *Snord116* and its immediate flanking 5’ intron sequence (pcDNA3.1(+) MBII-85-5'/3'ss mut, Cat No. 67645) was constructed by Falaleeva et al and obtained from Addgene (Falaleeva et al, 2015). QuikChange II XL Site-Directed Mutagenesis Kit (Cat No. 200521) was obtained from Agilent. Rabbit mAb to phospho-P42/44 ERK1/2 (Cat No. 4370), mouse mAb to P42/44 ERK1/2 (Cat No. 4696), rabbit mAb to phospho-MARCKS (Ser167/170) (D13E4) (Cat No. 8722), and mouse mAb to β-actin (8H10D10) (Cat No. 3700) were from Cell Signaling Technology. Mouse mAb to MARCKS (JK-8) (Cat No. sc-100777) was obtained from Santa Cruz. Rabbit pAbs to DGKK (Cat No. LS-C167384 and Cat No. LS-C358962) were obtained from LSBio. Mouse mAb to FLAG (M2) (Cat No. F1804) was purchased from Sigma-Aldrich. iQ SYBR Green Supermix was from Bio-Rad. A mutant SH-SY5Y line, A16, in which *SNORD116* expression was knocked down by 50%, and the parental SH-SY5Y line were provided by Bochukova et al (2018). Human iPSC line with the PWS T1 del and the isogenic normal control iPSC line (MGH2069) were provided by Dr. Michael Talkowski. Unmodified SH-SY5Y (CRL-2266) and SK-N-FI (CRL- 2142) human neuroblastoma cells used for *SNORD116* and *FBL* KD experiments were from American Type Culture Collection. LNA gapmer (ASO, 5′-TCA​CTC​ATT​TTG​TTC​A-3′) to human *SNORD116* (116Gapmer) and the LNA gapmer negative control A (5′-AAC​ACG​TCT​ATA​CGC-3′) were from Qiagen as previously reported (Baldini et al, 2022). Silent Select siRNAs to human *FBL* (Cat No. 4392420, siRNA ID s4822) and Silencer Select negative control siRNA (Cat No. 4390843) were from Thermo Fisher Scientific. Total RNA Purification Plus Micro Kit was from Norgen Biotek (Cat No. 48500). All RNAscope reagents, including Multiplex Fluorescent Reagent Kit v2 (Cat No. 323100), probes for mouse *Snord116* (Cat No. 1222641-C2) and mouse *Dgkk* (Cat No. 1128671-C1), and positive and negative control probes were from Advanced Cell Diagnostics. Lipofectamine 2000, Lipofectamine RNAiMAX, Pierce Primary Neuron Isolation Kit (Cat No. 88280), M-MLV reverse transcriptase, random hexamer, Pierce BCA Protein Assay Kits (Cat No. 23227), and all tissue culture reagents were from Thermo Fisher Scientific. PCR primers, TaqMan probes, and chimeras for the Nm-VAQ assay were from Integrated DNA Technology; their sequences are reported in Tables S8, S9, and S10. DHPG (Cat No. 0805) was purchased from Tocris. 5-Fluoro-2-deoxyuridine (F0503), laminin/poly-D-lysine coating solution (Cat No. LPDL001), and all other chemicals were from Sigma-Aldrich.

Table S8. qRT–PCR primers used for gene expression analyses.

Table S9. Primers used for genotyping and sex determination.

Table S10. Sequences of primers (5′-3′) used in 2′-O-methylation assays and mutagenesis.

### Animal husbandry, genotyping, and tissue collection

*B6.Cg-Snord116*^*tm1.1Uta*^*/J* (Stock number: 008149, *Snord116* deletion line) and B6.129-S*nrpn*^*tm2Cbr*^*/*J (Stock number 012443, IC del deletion line) were obtained from Jackson Laboratory. Male heterozygous *B6.Cg-Snord116*^*tm1.1Uta*^*/J* mice were crossed with female WT C57BL/6J mice to generate *Snord116* KO mice (*Snord116*^*p-/m+*^, hereafter referred to as *Snord116* KO) and their WT littermates for molecular and phenotypic characterizations. Male heterozygous B6.129-Snrpn^tm2Cbr^/J mice were crossed with female FVB mice to generate F1 progeny segregating for the *Snrpn*^*tm2Cbr*^ mutation. The F1 progeny heterozygous for the *Snrpn*^*tm2Cbr*^*/*J mutation (hereafter referred to as IC del) and their WT littermates were used for molecular and phenotypic characterization. Ear notch or tail DNAs were used for genotyping as previously described (Yang et al, 1998; Ding et al, 2008). A compounded TaqMan assay for *Sry*, a gene located on the Y chromosome (present only in males), and for a DNA segment from chromosome 6 (present in both sexes) was used to determine the sex of young pups. The sequences of primers and probes used for genotyping and sex determination are shown in Supplementary Information. All mice were kept in a barrier facility and maintained at 22^o^C with 12:12-h light: dark cycle and ad libitum access to rodent breeder chow (PicoLab Rodent Chow 5058) or a high-fat diet containing 60% fat calories (D12492; Research Diets Inc) as indicated, and water. Both diets contained 20% protein calories. Mice were euthanized with CO2 asphyxia followed by cervical dislocation. After euthanasia, the hypothalamus was collected, flash-frozen in liquid nitrogen, and stored at −80^o^C for future analyses. All animal husbandry and procedures were in compliance with the accepted standards of animal care and were approved by the Columbia University Institutional Animal Care and Use Committee.

### RNAseq and data analysis

Total RNA was extracted using Total RNA Purification Plus Micro Kit following the manufacturer’s instructions. RNA quality was assessed using an Agilent Bioanalyzer 2100. Only samples with RNA integrity numbers >9 were used for RNAseq. Ribo-Zero Plus rRNA Depletion Kit and TruSeq Stranded Total RNA Library Prep Kit Gold (Illumina) were used for library preparation before cDNA was sequenced on a NovaSeq 6000 with paired-end 100 bp read length, 40 million read counts per sample. Library preparation and RNAseq were performed by JP Sulzberger Columbia Genome Center. RNAseq data were processed using Galaxy Version 21.01 (Afgan et al, 2018). Data quality was assessed using FastQC, and reads with quality scores < 20 were removed from the dataset. Reads were aligned to the Mouse GRCm38 genome using HISAT2, and aligned reads were counted over genomic features using FeatureCounts (Liao et al, 2014; Kim et al, 2015a). Differential expression analysis was performed using edgeR with minimal counts per million (CPM) of 0.5 in the number of samples equal to or greater than the smaller sample number (n) of the two groups being compared (Robinson et al, 2010). Statistical significance was set at threshold-adjusted *P*-value (FDR) <0.1 using Benjamini and Hochberg's algorithm. GSEA was performed using Galaxy fGSEA tool and curated MSigDB Hallmark, KEGG, and Reactome gene sets (Mootha et al, 2003; Subramanian et al, 2005).

### Enrichr analysis

GSEA for the DEGs was analyzed using the web-based Enrichr program (http://amp.pharm.mssm.edu/Enrichr) (Kuleshov et al, 2016).

### IntaRNA analysis

*In silico* analysis of RNA:RNA interaction was performed using a web-based IntaRNA 2.0 program (IntaRNA - RNA-RNA interaction) (Mann et al, 2017).

### qRT–PCR

Total RNA was reverse-transcribed into first-strand cDNA using M-MLV reverse transcriptase and random hexamer. Quantitative PCR was carried out using gene-specific primer and Bio-Rad iQ SYBR Green Supermix or gene-specific TaqMan assays. Two housekeeping genes, cyclophilin A (*Ppia*) and TATA-box binding protein (*Tbp*), were used as controls to normalize the initial RNA input. Delta Ct was calculated using the geometric mean of *Tbp* and *Ppia* for each sample. The delta–delta Ct method was used to calculate relative expression levels in samples assayed together. qRT-PCR primers used in this study are listed in Supplementary Information.

### Gene KD and other procedures in cultured cells

SH-SY5Y human neuroblastoma line A16 and its parental SH-SY5Y lines were grown in DMEM/F-12 supplemented with 15% FBS, 100 IU and 100 μg/ml penicillin/streptomycin, and 2 mM GlutaMAX. SK-N-FI human neuroblastoma cells were cultured in DMEM supplemented with 10% FBS and 0.1 mM nonessential amino acids, 100 IU and 100 μg/ml penicillin/streptomycin, and 2 mM GlutaMAX. Cells were maintained at 37°C with 5% CO2 in a humidified chamber. For *SNORD116* KD in SH-SY5Y and SK-N-FI cells, 30 pmole of antisense LNA gapmer to human *SNORD116* was introduced into 0.35 million cells using Lipofectamine 2000 following the reverse transfection protocol (Zhang et al, 2007; Baldini et al, 2022, ref 13). LNA gapmer negative control A was used as a negative control. At 48 and 24 h post-transfection, respectively, SK-N-FI and SH-SY5Y cell lysates were collected for RNA and protein analyses. For siRNA-mediated KD of human *FBL* in SK-N-FI cells, Silent Select siRNAs to human *FBL* (15 pmole) were reverse-transfected into 0.35 million cells using Lipofectamine RNAiMAX. Silencer Select negative control siRNA was used as a negative control for *FBL* KD. Transfected cells were cultured in 1 ml culture medium in a 12-well plate. 48 h post-transfection, cell lysates were collected for analysis. For *DGKK* mRNA stability assay in SK-N-FI cells with or without *SNORD116* KD, cells were treated with 5 μg/ml actinomycin D, a transcription inhibitor, for 0, 8, and 24 h before cell lysates were collected for RNA analysis. For overexpression of FLAG-tagged human *DGKK* and mouse *DGKK* in HEK293 cells or SH-SY5Y cells, plasmids encoding FLAG-tagged human *DGKK* and mouse *Dgkk* cDNA were reverse-transfected into HEK293 cells using Lipofectamine 2000 (Zhang et al, 2007). 48 h post-transfection, cell lysates were collected for Western blotting analysis. All experiments using cultured cells were performed in triplicate or quadruplicate and repeated at least three times.

### RNAscope assay

Brains of 9-wk-old WT and *Snord116* KO mice were flash-frozen on powdered dry ice and embedded in OCT. Coronal sections (20 μm) containing brain regions of interest were fixed in freshly prepared 4% PFA and labeled with a specific probe or probes using RNAscope Multiplex Fluorescent Reagent Kit v2 from Advanced Cell Diagnostics following the manufacturer’s recommendation. The RNAscope probe for mouse *Snord116* targets a 45-nt segment of *Snord116* (9–54 nt of LN872796). The RNAscope probe for mouse *Dgkk* targets a 945-nt region of the gene (1091–2035 ant of NM_177914.3). Probes for mouse *Snord116* and mouse *Dgkk* were designed by and purchased from Advanced Cell Diagnostics. Images of RNAscope were captured using a Nikon Eclipse 80i epifluorescence microscope or a Zeiss LSM 710 confocal microscope.

### RTL-P and Nm-VAQ assays for 2′-O-methylation of human *DGKK* mRNA

RTL-P and Nm-VAQ assays were performed as previously described (Dong et al, 2012; Tang et al, 2024). Briefly, for the RTL-P assay of Um1483 of human *DGKK* (Accession: NM_001013742.4), total RNA was reverse-transcribed using anchored and unanchored primers specific to U1483 of *DGKK* at high (500 μM) and low (10 μm) dNTP concentrations. The amount of cDNA in each RT reaction was quantified by qRT–PCR using Bio-Rad iQ SYBR Green Supermix as described. *TBP* was used as a control for the RT efficiency of each sample. The sequences of *DGKK* RT primers and qRT–PCR primers are listed in Supporting Information. For the Nm-VAQ assay of Um1483 of *DGKK* mRNA, total RNA was first hybridized with a methylation site–specific chimera for *DGKK*, followed by RNase H digestion or mock RNase H digestion in RNase H digestion buffer supplied by the manufacturer. Both digested and undigested hybridization mixtures were then subjected to qRT–PCR assay to quantify *DGKK* transcripts. The fraction of *DGKK* mRNA molecules protected from RNase H digestion, that is, the fraction of *DGKK* mRNA that was 2′-O-methylated at U1483, was calculated using the delta–delta Ct method. G585 of human *GAPDH* mRNA (Accession: NM_001289745.2), which was not 2′-O-methylated (Tang et al, 2024), was used as a control. The sequences of the chimera and RT–PCR primers used in Nm-VAQ assays are shown in Supporting Information.

### Compensatory mutation analysis

The plasmid encoding C-terminal FLAG-tagged human *DGKK* in pcDNA3.1^+^/C-DYK vector (pcDNA3.1^+^hDGKK/C-FLAG) and the plasmid encoding mouse *Snord11*6 and its immediate flanking 5′ intron sequence (pcDNA3.1(+) MBII-85-5′/3′ss mut) were used to generate substitution mutation in the respective predicted interacting sites of *DGKK* and *Snord116* using QuikChange II XL Site-Directed Mutagenesis Kit and gene-specific mutagenesis primers (*DGKK*, F: TCC​TAT​GTT​GCT​TCA​GAA​GC; R: ACC​AAA​ACC​GTC​TCC​TAC​AG; *Snord116*, F: AAA​AGC​TGA​ACA​AAA​TGA​G; R: ATG​TTT​GAC​TGG​GAA​TC). The 7-bp seeding sequence (tccttgg) in the predicted interacting site of the WT *Snord116* was substituted with the complementary sequence (aggaacc) in the mutant *Snord116* (*Snord116*_*mu*_) and that 7-bp seeding sequence (ccaagga) in the predicted interacting site of the WT *DGKK* was substituted with the complementary sequence (ggttcct) in the mutant *DGKK* (*DGKK*_*mu*_). The WT and mutant *DGKK* and *Snord116* constructs were then transfected into HEK293A cells in various combinations as described in Results. 48 h post-transfection, total RNA was extracted and used for 2′-O-methylation analysis of *DGKK* at U1483.

### MCN culture and activation of GluR1/5 signaling by DHPG

MCNs were isolated from P0 pups using Pierce Primary Neuron Isolation Kit following the manufacturer’s recommendation with minor modifications. Briefly, the cortex from each pup was digested with papain and dissociated by pipetting up-and-down 15–20 times using P1000 Pipetman. The dissociated cells were filtered through a 40-μm cell strainer (blue) and centrifuged at 500*g* × 5 min to remove tissue debris. Cells were then resuspended in the plating medium (Neurobasal medium supplemented with 10% HI-FBS, 2 mM GlutaMAX) and seeded in a laminin/poly-D-lysine–coated 12-well plate at 0.6 million cells per well in 1 ml plating medium. After 1 h of incubation in a humidified CO_2_ incubator at 37^o^C, the plating medium was removed and replaced with growth medium (Neurobasal medium supplemented with 2% B27, 0.5 mM GlutaMAX, 100 IU and 100 μg/ml penicillin/streptomycin, and 10 mM Hepes, pH 7.4). Cells were cultured for 3 d before they were treated with 5-fluoro-2-deoxyuridine at a final concentration of 5 μM to suppress the proliferation of mitotic cells. Cells were fed weekly by replacing 0.33 ml medium (1/3 of the total medium volume) with fresh growth medium. To activate group 1 glutamate receptors (GluR1/5), cells were treated with 100 μM DHPG, a selective group 1 glutamate receptor agonist for 0–30 min. Cell lysates were collected in RIPA buffer with protease and phosphatase inhibitors as previously described (Guo et al, 2007).

### Protein extraction and immunoblotting

Cell lysates were analyzed by SDS–PAGE and Western blotting using enhanced chemiluminescence as previously described (Guo et al, 2007). Unless otherwise indicated, human DGKK in cell lysates was detected using rabbit anti-human DGKK polyclonal antibodies. FLAG-tagged human and mouse DGKK were also detected using mouse monoclonal anti-FLAG (M2) antibody. Quantification of Western blotting results was performed using Quantity One software (Bio-Rad, Hercules, CA).

### Immunofluorescence

SH-SY5Y cells transfected with FLAG-tagged human *DGKK* were seeded on poly-D-lysine–coated chamber slides. Cells were fixed with freshly prepared 4% PFA in PBS for 15 min at room temperature and permeabilized with 0.3% Triton X-100 for 5 min. Permeabilized cells were blocked for 30 min in PBS/1% BSA/5% normal control serum (from the species in which the fluorescence-conjugated secondary antibody was made), and incubated with primary antibody diluted in PBS/1% BSA overnight at 4^o^C, followed by fluorescence-conjugated secondary antibody for 2 h at room temperature. Vehicle and transfected cells were stained in parallel and photographed using the same exposure settings.

### Statistical analysis

GraphPad Prism was used for statistical analysis. Data are presented as the mean ± SEM of triplicate or quadruplicate samples from a typical single experiment unless otherwise indicated. Differences between control and experimental groups were examined using a two-tailed *t* test or ANOVA; *P* < 0.05 was considered statistically significant.

## Supplementary Material

Reviewer comments

## Data Availability

RNAseq data of P5 hypothalamus of *Snord116* KO mice and WT controls are available at the NCBI GEO database with the Accession number GSE346487. RNAseq data of P5 hypothalamus of mice with PWS Imprint Center deletion and WT controls are available at the NCBI GEO database with the Accession number GSE346491.
