# Supplementary material for: Diacylglycerol kinase kappa is a target of the Prader–Willi syndrome gene SNORD116
Source: Life Sci Alliance. 2026 Nov 4;10(1):e202603723. doi: 10.26508/lsa.202603723 (PMC13736183; doi:10.26508/lsa.202603723)
Supplement: Supplementary file 1 [file LSA-2026-03723_review_history.pdf]

# Diacylglycerol kinase kappa is a target of the Prader-Willi Syndrome gene SNORD116

Yiying Zhang, Kaiying Guo, Gabriel Batzli, Haoquan Zhao, Lisa Burnett, Charles LeDuc, George Stratigopoulos, Daniel Carolan, Claudia Doege, Yufeng Shen, and Rudolph Leibel
**DOI: https://doi.org/10.26508/lsa.202603723**

*Corresponding author(s): Yiying Zhang, Columbia University Irving Medical Center and Rudolph Leibel,*



## Review Timeline:

| | |
|---|---|
| Submission Date: | 2026-03-31 |
| Editorial Decision: | 2026-05-21 |
| Revision Received: | 2026-09-05 |
| Editorial Decision: | 2026-10-06 |
| Revision Received: | 2026-10-16 |
| Accepted: | 2026-10-20 |

*Scientific Editor: Sarita Hebbar*


## Transaction Report:

(Note: With the exception of the correction of typographical or spelling errors that could be a source of ambiguity, letters and reports are not edited. The original formatting of letters and referee reports may not be reflected in this compilation.)

May 21, 2026

Re: Life Science Alliance manuscript #LSA-2026-03723-T

Dr. Yiying Zhang
Columbia University
Pediatrics

Dear Dr. Zhang,

Thank you for submitting your manuscript entitled "Diacylglycerol kinase kappa is a novel target of the Prader-Willi Syndrome gene SNORD116" to Life Science Alliance.

Your manuscript was reviewed by three expert reviewers. As you will read, these reviewers have remarked that the identification of a potential target for SNORD116 regulation is of potential value. That said, the reviewers have raised several significant concerns around claims made in this work and a general lack of clarity and synthesis of the described results. Based on these, we invite you to submit a revised version resolving

- all conceptual concerns (moderation of claims, expand and adjust discussion on findings, include a proper justification/rationale, appropriately contextualise the findings in the context of published information)
- all technical concerns (include relevant controls, data presented in Figure 5, FBL knockdowns, negative control for methylation analyses, and quality of imaging data).

I would be happy to discuss the revision in more detail via email or phone/videoconferencing. Please let me know which option you prefer, if any.

To upload the revised version of your manuscript, please log in to your account: https://lsa.msubmit.net/cgi-bin/main.plex

You will be guided to complete the submission of your revised manuscript and to fill in all necessary information. Please get in touch in case you do not know or remember your login name.

The typical timeframe for revisions is three months. Please note that papers are generally considered through only one revision cycle, so strong support from the referees on the revised version is needed for acceptance.

While you are revising your manuscript, please also attend to the below editorial points to help expedite the publication of your manuscript. Please direct any editorial questions to the journal office. When submitting the revision, please include a letter addressing the reviewers' comments point by point.

Thank you for this interesting contribution to Life Science Alliance. We hope that the comments below will prove constructive as your work progresses, and we are looking forward to receiving your revised manuscript.

Sincerely,

Sarita Hebbar, PhD
Scientific Editor
Life Science Alliance
http://www.lsajournal.org

------------------------------------------------------------------------------

A. THESE ITEMS ARE REQUIRED FOR REVISIONS

-- A letter addressing the reviewers' comments point by point.

-- An editable version of the final text (.DOC or .DOCX) is needed for copyediting (no PDFs).

-- High-resolution figure, supplementary figure and video files uploaded as individual files: See our detailed guidelines for preparing your production-ready images, https://www.life-science-alliance.org/authors

-- Summary blurb (enter in submission system): A short text summarizing in a single sentence the study (max. 200 characters including spaces). This text is used in conjunction with the titles of papers, hence should be informative and complementary to

the title and running title. It should describe the context and significance of the findings for a general readership; it should be written in the present tense and refer to the work in the third person. Author names should not be mentioned.

-- By submitting a revision, you attest that you are aware of our payment policies found here: https://www.life-science-alliance.org/copyright-license-fee

B. MANUSCRIPT ORGANIZATION AND FORMATTING:

Full guidelines are available on our Instructions for Authors page, https://www.life-science-alliance.org/authors

We encourage our authors to provide original source data, particularly uncropped/-processed electrophoretic blots and spreadsheets for the main figures of the manuscript. If you would like to add source data, we would welcome one PDF/Excel-file per figure for this information. These files will be linked as supplementary "Source Data" files.

***IMPORTANT: It is Life Science Alliance policy that if requested, original data images must be made available. Failure to provide original images upon request will result in unavoidable delays in publication. Please ensure that you have access to all original microscopy and blot data images before submitting your revision.***

------------------------------------------------------------------------

Reviewer #1 (Comments to the Authors (Required)):

The study reports the identification of a putative gene target of the Prader-Willi syndrome (PWS) -specific non-coding RNA SNORD116. Based on transcriptional analyses of the hypothalamus, the tissue most affected by PWS, in two mouse models of the disease, a small number of genes with altered transcription levels were identified, including DGKK. The authors then focus their study on DGKK, demonstrating that its expression is similarly increased in human iPSC PWS cells and in cells where SNORD116 is knocked down, including at the protein level. This provides support for the regulation of DGKK by SNORD116. Additionally, the authors present evidence of SNORD116-specific methylation of DGKK mRNA, hypothalamic-specific expression of DGKK, and alterations in diacylglycerol (substrate of DGKK) signaling in cortical neurons of Snord116-KO mice. Finally, they describe a metabolism-dependent regulation of DGKK synthesis and propose a global regulatory mechanism in which DGKK, as a regulator of various Gq receptors, may play a key role in PWS mechanisms.
Overall the study presents a number of interesting data, however, a better justification for focusing on DGKK, clarification of methological details, and resolution of some inconsistencies need to be adressed.
Major issues :
- While the study raises an interesting and potentially important link between SNORD116 and DGKK, the current data do not provide clear evidence to establish DGKK as a direct molecular target of SNORD116. Thus, it would be appropriate to moderate several statements that currently appear too definitive, including the title of the manuscript and one subheading of the Discussion.
- In line with this, the statement in the Introduction that "We identified diacylglycerol kinase kappa (Dgkk) as a potential target of Snord116" is more appropriately balanced. However, an important issue is that this observation is not presented with sufficient acknowledgment of prior work as the SNORD116-DGKK interaction was already reported in Baldini et al., 2021. The same concern applies to Fig. 4B, the intRNA analysis, and the statement in the Results section that "We detected a strong interaction between the antisense element 1 of Snord116 and exon 8 of Dgkk mRNA in both mouse and human." Similarly, transient knockdown with a Snord116-specific Gapmer appears to extend an approach that was already described in Baldini et al. It is very important to more clearly distinguish between findings that are novel and those that confirm or build upon earlier studies. For instance, the sentence "Transfection of human neuroblastoma SK-N-FI cells [...] negative control ASO (Fig. 6B-C)" should explicitly indicates that these results are consistent with and confirm previous work (Baldini 2021). In the same line, the hypothesis that DGKK may undergo 2′-O-methylation directed by SNORD116 has also been mentioned in a recent publication (PMID: 41446073). Thus, more precise positioning of the present study with respect to the literature is necessary, as it is essential to clearly distinguish between novel findings and data that replicate or confirm earlier work.
- The pathway analyses in the two models (Imprinting Center deletion, IC del, and Snord116 KO hypothalamus from postnatal day 5 pups) identified various pathways, but with low stringency (q-value threshold of ~0.1, i.e., 10% false discovery rate). No validation of these data is provided. Why did the authors not further investigate Fgd2 or the other 10 genes reported in Figure 1? It is unclear whether any of these genes could be targeted by Snord116, or if Dgkk is the only possible target among them.
- The use of FBL knockdown for snoRNA-dependent methylation activity is not, to our knowledge, a standard approach. As FBL is an essential factor for cell viability and protein synthesis, even transient downregulation is likely to induce substantial cellular stress, which should have been monitored and documented. In agreement, it is plausible that the discrepancy between the effects of Snord116 and FBL knockdown reflects indirect consequences of FBL depletion. The authors should therefore either provide appropriate controls demonstrating that FBL knockdown did not induce confounding cellular stress, or substantially temper the interpretation of this experiment.
- The Nm-VAq analysis presents three important limitations that should be addressed:
1. In human, sequence variation between SNORD116 group I and group II copies (with group II being predominantly expressed in tissues including the brain) predicts two possible DGKK methylation sites, U1483 and C1482. Since there is no clear rationale for testing only one of these positions the analysis should include both candidate sites.

2. RNase H digestion is often suboptimal in a Gapmer-dependent fashion, which can directly affect the quantification of methylation. To control for this, it is standard practice to use synthetic RNAs corresponding to the sequence of interest, carrying or lacking a 2′-O-methylation at the candidate site. Such controls would help determine whether the methylation percentages reported here reflect true biology or simply incomplete digestion efficiency. In the same line, it is surprising to control unmethylated sites at an unrelated RNA and not another (often close) position on the same RNA. These validation steps should be included.
3. Rather than presenting the results in graphical form, the authors provide tables containing the individual Ct values of three replicates. The conclusions are based on mean Ct values, while standard deviations are not reported. In addition, PCR efficiency appears suboptimal: for example, in Fig. 7B, the use of 4 ng versus 18 ng of cDNA (a 4.5-fold input difference) yields Ct differences of 1.5 and 3.1 for Dgkk Ct1 and Ct2, corresponding to apparent ratios of 2.8 and 8.6, respectively. The PCR conditions should therefore be optimized to achieve robust amplification efficiency, and, most importantly, appropriate statistical analyses should be performed to support the authors' conclusions.
- The RTL-P data should be incorporated into the main figures of the manuscript, together with the appropriate controls, as these experiments are directly relevant to the main conclusions.
- RNA-seq data are classically validated by an orthogonal method (commonly RT-qPCR), at least for the genes that are used to support the main conclusions of the study. This would be particularly important for genes such as Snord64, Dhcr7, and the additional genes belonging to the pathways discussed by the authors. This represents a limited amount of additional work that will strengthen confidence in the dataset and in the corresponding interpretation.
Table 1: The relative expression values of DGKK across brain regions are not clearly defined. Does "++" indicate a two-fold increase compared to "+"?
Figure 5C: The quality of the Western blot (WB) is questionable; T1 del samples appear more loaded than wild-type (WT), and it seems as if number of lanes in ß-actin and in DGKK is different. "D" missing on figure.
Figure 8: The modified RNAscope technique used to assess Dgkk mRNA expression in the adult mouse brain lacks a specificity control (e.g., probe validation in expressing vs. non-expressing cells).
Figure 9A-B: The nuclear localization of DGKK with SNORD116 is unclear due to insufficient image quality. The conclusion cannot be confidently drawn from the provided data.
Figure 10: It is intriguing that cortical neurons were chosen to investigate DAG signaling, given that DGKK is minimally expressed in this brain region (or not at all, according to Figure 9). The authors should justify this choice or discuss potential influence of signaling circuits.
Figure 11: The effect of fasting on Dgkk expression should include details on data normalization.
The statement "Expression of E2f target genes [...] was negatively enriched in Snord116-deficient mice, indicating a decreased proliferating cell population and/or precocious neuronal differentiation" is an overinterpretation. The pathway analysis only provides suggestive evidence, and no validation by other methods was performed. A more cautious tone is warranted.
Other minor comments :
The scheme mentioned in the text (page 5) should be counted and referred to as a figure.
Abbreviations should be listed in alphabetical order.
Graph representations should be standardized across figures.
Page 12: The meaning of MCH is unclear.
Page 14: Typo: Snoed116 → Snord116.
Page 15, line 13: Citation 33 does not seem relevant in this context.

Reviewer #2 (Comments to the Authors (Required)):

Zhang et al describe DGKK as a novel target for SNORD116

SNORD116 is an orphan SNORD that plays a key role in Prader-Willi syndrome. However, targets and mechanistical insights have been elusive. Thus identification of a new target as shown could be highly interesting and important.

The key finding is the sequence complementarity between SNORD116 and DGKK shown in Figure 7A. Do the other genes shown in Figure 1A have SNORD116 binding sites? - or the genes that are similar between the ko models (Figure 1B)?
The 2'0-methylation should be recapitulated with reporter genes, where compensatory mutations and or RNA binding assays (RIP) could be employed. Currently, the data in fig 7 are not fully convincing (see below).

Figure 1A: all the genes and their fold changes should be presented in a table /Xls file. There have been several similar studies looking at SNORD116 knock outs (mostly human), and SNORD116 overexpression, which should be compared as well.

Figure 5 C/D: the label D is missing in the figure. Figure 5C is essential for the paper, but of poor quality. There is a non-specific band under DGKK in the negative control that disappears in the transfected positive control. The actin loading control is overexposed, making the quantification in D questionable. The complete gel including size makers should be shown.

Figure 7 is the key mechanistical insight into the SNORD116-DGKK interaction. SNORD116 was knocked down with a gapmer. Why was not the crispr-based total (genetic) kd made in SHSY cells used? If SNORD116 causes the 2'O-methylation, it should do down to zero in the kd. In addition, ko mouse RNA could be used.

The most reliable way to measure 2'0-methylation is primer extension under limited dNTP concentration, which should be tried, as it gives less errors than the PCR based assays. Finally, a negative control, like 2'O-methylation of a rRNA should be used, which should also respond to the fibrillarin kd.

Figure 10 measures ERK and PKC signaling in response to SNORD116 kd, /DGKK increase. It is hard to make the connection to DGKK as there are at least 10 more diacetyl glycerol kinases in humans that could do the same reaction as DGKK.

Minor point: the paper has 12 figures and should be streamlined. A lot of the discussion about the sequences could go into the supplmenent.

Reviewer #3 (Comments to the Authors (Required)):

Zhang et al show convincing data for Snord116 regulates molecular pathways. Dgkk was isolated from other DEGs shared by both the mouse IC deletion and Snord116 KO. They show Snord116 and Dgkk expression are inversely correlated, and that Snord116/FBL complex may 2'-O methylate the Dgkk transcript. This involvement is unlikely to be exclusive, as there are additionally noncanonical functions of the Snords with potential overlapping functions. Downstream implications suggest dysfunctional nervous system development, impaired synapse maturation, and pathophysiological membrane signaling.
Major Points
1) There is still some debate in the field whether SNORD116 mutations alone are sufficient to recapitulate the full spectrum PWS phenotype. The phenotypic overlap with MAGEL2 truncating mutations, that cause SYS, supports the notion that PWS in most cases is caused by a combined loss of both SNORD116 and MAGEL2. That changes the impact of the 700 transcripts which were DE in the brain of the IC deletion mouse - which would include both SNORD116 and MAGEL2. There is, in fact, no discussion of MAGEL2 in the manuscript. The authors need to address this discrepancy and perhaps take a closer look at genes regulated by the loss of BOTH SNORD116 and MAGEL2. Many more DEGs detected for the IC Del vs Snord116 mutant, I would like to seem the differences addressed before focusing on the overlap only. What are the top DEGs in IC del? Where are the overlap DEGs found in the ranked list of DEGs for the IC del? Do they stratify near the top for logFC? Or are other genes/pathways with more change in the IC del group. I'd like to see the remaining 6 significant DEGs for Snord116 ko addressed, are those DEGs meaningful?
2) Along these same lines, the authors do not justify why the did NOT take a closer look at the Fgd2 gene, which in Figure 1B is up regulated just as much as Dgkk. Why not look into this regulation?
3) If they think that loss of SNORD116 up regulates Dgkk in critical regions of the brain controlling feeding behavior and energy balance, why did they not look at either the IC del mouse or even the SNORD116 KO mouse brain for elevated Dgkk? Certainly they would want to show this by RNAscope!
4) Given the focus on feeding behavior and metabolism, wouldn't the SNORD116 KO mice display hyperphagia? Apparently, they do not - according to the literature.

Minor points
1) Fig 5c, the western blot shows a large nonspecific band that is also missing in WT. There is only one experimental replicate for this western blot and the western blot in Figure 10. The western in figure 10 contains two technical replicates(?) on a single gel and suggests a link with mGluR activity, however additional experimental replicates are needed to support this claim. The figure is labeled DIV14 but the caption shows DIV16, need to correct.
2) Snord116 was misspelled at the beginning of the discussion.

## Point-by-point response to Reviewer's comments

**Reviewer #1 (Comments to the Authors (Required)):**

The study reports the identification of a putative gene target of the Prader-Willi syndrome (PWS) -specific non-coding RNA SNORD116. Based on transcriptional analyses of the hypothalamus, the tissue most affected by PWS, in two mouse models of the disease, a small number of genes with altered transcription levels were identified, including DGKK. The authors then focus their study on DGKK, demonstrating that its expression is similarly increased in human iPSC PWS cells and in cells where SNORD116 is knocked down, including at the protein level. This provides support for the regulation of DGKK by SNORD116. Additionally, the authors present evidence of SNORD116-specific methylation of DGKK mRNA, hypothalamic-specific expression of DGKK, and alterations in diacylglycerol (substrate of DGKK) signaling in cortical neurons of Snord116-KO mice. Finally, they describe a metabolism-dependent regulation of DGKK synthesis and propose a global regulatory mechanism in which DGKK, as a regulator of various Gq receptors, may play a key role in PWS mechanisms.

Overall the study presents a number of interesting data, however, a better justification for focusing on DGKK, clarification of methodological details, and resolution of some inconsistencies need to be addressed.

Major issues :
1) -While the study raises an interesting and potentially important link between SNORD116 and DGKK, the current data do not provide clear evidence to establish DGKK as a direct molecular target of SNORD116. Thus, it would be appropriate to moderate several statements that currently appear too definitive, including the title of the manuscript and one subheading of the Discussion.

**Response**: We appreciate Reviewer's comments and endeavor to make accurate statements that are supported by the data and in reference to published studies in the literature.  In the revised manuscript, we add new experimental results, including compensatory mutation analysis, which further support the inference that *DGKK* is a direct target of *SNORD116*. Considering our data in totality as well as results in the literature, we modified the title to "Diacylglycerol kinase *kappa* is a target of the Prader-Willi Syndrome gene *SNORD116*" and kept the referred subtitle in Discussion as is, i.e., "DGKK is a direct target of *Snord116*".

2) - In line with this, the statement in the Introduction that "We identified diacylglycerol kinase kappa (Dgkk) as a potential target of Snord116" is more appropriately balanced. However, an important issue is that this observation is not presented with sufficient acknowledgment of prior work as the SNORD116-DGKK interaction was already reported in Baldini et al (Baldini et al, 2022, PMCID: PMC8789076). The same concern applies to Fig. 4B, the intRNA analysis, and

the statement in the Results section that "We detected a strong interaction between the antisense element 1 of Snord116 and exon 8 of Dgkk mRNA in both mouse and human." Similarly, transient knockdown with a Snord116-specific Gapmer appears to extend an approach that was already described in Baldini et al (Baldini et al, 2022, PMCID: PMC8789076). It is very important to more clearly distinguish between findings that are novel and those that confirm or build upon earlier studies. For instance, the sentence "Transfection of human neuroblastoma SK-N-FI cells [...] negative control ASO (Fig. 6B-C)" should explicitly indicate that these results are consistent with and confirm previous work (Baldini et al, 2022, PMCID: PMC8789076). In the same line, the hypothesis that DGKK may undergo 2′-O-methylation directed by SNORD116 has also been mentioned in a recent publication (PMID: 41446073). Thus, more precise positioning of the present study with respect to the literature is necessary, as it is essential to clearly distinguish between novel findings and data that replicate or confirm earlier work.

**Response:** We agree with the reviewer that explicit acknowledgement of previous work is very important. We have made changes in the appropriate places (Introduction, Results and Discussion) in the revised manuscript, including the following paragraph in Discussion (Page 19): "Gene expression profiling has been used to identify *SNORD116* molecular targets in various model systems, including post-mortem hypothalami from PWS patients (Bochukova et al, 2018, PMC5896230, ref 17), fibroblast lines from PWS patients (Burnett et al, 2017, PMC5199710), HEK293 cells overexpressing *Snord116* (Falaleeva et al, PMC5586535), and neurons derived from WT and *SNORD116*-deficient human ES cells or human LUHMES neuronal progenitor cells (Gilmore et al, 2024, PMC11662933;Helwak et al, 2024 PMC11608373). The potential *SNORD116* target genes identified in these studies showed little overlap with each other or with the shared hypothalamic DEGs between IC del and *Snord116* KO mouse models identified in the current study, including *DGKK*. The lack of such overlap may have multiple causes, including differences in the model systems, genetic background (between samples within a study and between different studies), and differentiation stage of the neurons analyzed. *DGKK* has been implicated as a potential *SNORD116* target in two isogenic cellular models (Baldini et al, 2022 and Ye et al, 2025). Consistent with these reports, our physical and physiological data obtained from isogenic animal and cellular models provide independent and substantial evidence that *DGKK* is a *bona fide* functional target of *SNORD116*."

Please see Pages 6, 7, 9, 11 for other relevant modifications addressing this point.

Additionally, using compensatory mutation analysis, we have obtained additional evidence suggesting that the predicted interaction between *SNORD16* and DGKK is required for 2′-O-methylation of U1483 in human DGKK mRNA (Fig 6F-6G in the revised manuscript). Briefly, we mutated the seeding sequence in the predicted respective interacting sites of the DGKK-FLAG reporter construct and *SNORD116*-expressing plasmid. Disruption of the sequence complementarity by mutating only *Snord116* or DGKK significantly decreased 2′-O-methylation of U1483 in HEK293 cells co-transfected with *SNORD116* and *DGKK*-FLAG reporter constructs. In contrast, restoration of the sequence complementarity by mutating both the *DGKK*-

FLAG reporter and *SNORD116* restored 2′-O-methylation of U1483 to a level similar to that of the WT *DGKK*-FLAG reporter and *Snord116*. These new results support the inference that *DGKK* is a direct target of *SNORD116*. We discussed the new results and have modified discussions to reflect more concise/accurate interpretations of the relevant results (Pages 12, 13, 18-19).

3) - The pathway analyses in the two models (Imprinting Center deletion, IC del, and Snord116 KO hypothalamus from postnatal day 5 pups) identified various pathways, but with low stringency (q-value threshold of ~0.1, i.e., 10% false discovery rate). No validation of these data is provided. Why did the authors not further investigate Fgd2 or the other 10 genes reported in Figure 1? It is unclear whether any of these genes could be targeted by Snord116, or if Dgkk is the only possible target among them.

**Response:** These are important points which were also raised by Reviewers 2 and 3. We performed IntaRNA and RT-qPCR analyses on all shared DEGs in Fig 2B (of the revised MS) as part of the preliminary vetting process. These results are now included in the revised manuscript as Supplemental Table S6 and Figs S2-S3. Briefly, IntaRNA analyses showed that several of the shared DEGs, including *Ankrd52, Ank, Hipk3, and Tmem231*, may interact with *Snord116* with similar strength as *Dgkk* (minimal free energy ≤ -13 kcal/mol). qRT-PCR confirmed the differential expression status of several shared DEGs in P5 hypothalamus from an independent cohort of *Snord116* KO and WT controls (Supplemental Fig S2). RT-qPCR analyses also confirmed the differential expression status of *Dgkk* in P5 hypothalamus of the IC del model (Supplemental Fig S2). However, none of the shared DEGs, except *Dgkk* were differentially expressed in the hypothalami of a small cohort of 12-week-old *Snord116* KO and WT controls (n=5) (Supplemental Fig S3). Based on these vetting results, we decided to focus on *Dgkk* in the subsequent studies. Results (Pages 9-10) has been modified to provide rationales for focusing on *Dgkk* in the subsequent studies.

4) - The use of FBL knockdown for snoRNA-dependent methylation activity is not, to our knowledge, a standard approach. As FBL is an essential factor for cell viability and protein synthesis, even transient downregulation is likely to induce substantial cellular stress, which should have been monitored and documented. In agreement, it is plausible that the discrepancy between the effects of Snord116 and FBL knockdown reflects indirect consequences of FBL depletion. The authors should therefore either provide appropriate controls demonstrating that FBL knockdown did not induce confounding cellular stress or substantially temper the interpretation of this experiment.

**Response:** We appreciate the point. *FBL* knockdown has been previously used to evaluate snoRNA-dependent 2′-O-methylation of *Pdx* (Elliot BA et al, PMC6667457) and global 2′-O-methylation of mRNAs (Tang et al, PMC10985248). We used the same siRNA as that used in the

study by Elliot et al., which is commercially available, to knock down human *FBL*. In our study, SK-N-FI cells transfected with siRNA to *FBL* and negative control siRNA were microscopically indistinguishable during the daily examination of the transfected cells. The average RNA yields (341 vs 309 ng/ul in 20 ul) and the average Ct values for the house-keeping genes *PPIA* (18.01 vs 18.15) and *TBP* (22.06 vs 22.13) were similar between *FBL* KD and NC controls, in one of the *FBL* knockdown experiments, for example. These results suggest that there was no gross cellular stress/toxicity associated with siRNA-mediated *FBL* knockdown in SK-N-FI cells. However, despite the absence of evidence that *FBL* knockdown induced cellular stress/toxicity in SK-N-FI cells, we agree with the reviewer that this possibility exists and have addressed this point in the Discussion in the revised manuscript (Page 20).

5) - The Nm-VAq analysis presents three important limitations that should be addressed:
1. In human, sequence variation between SNORD116 group I and group II copies (with group II being predominantly expressed in tissues including the brain) predicts two possible DGKK methylation sites, U1483 and C1482. Since there is no clear rationale for testing only one of these positions the analysis should include both candidate sites.

**Response:** 1. Strong RNA:RNA interactions were predicted between *DGKK* and mouse *Snord116* and group I human *SNORD116*s, which were predicted to facilitate/guide the methylation of U1483 of human *DGKK*. As the reviewer points out, the recent report by Ye et al showed that 2′-O-methylation of C1484 of human *DGKK* is *SNORD116*-dependent in stem cell derived human neurons (Ye et al, 2025, PMID: 41446073), which is expected to be mediated by group II *SNORD116* (We assume that the reviewer meant C1484, not C1482 because at the nucleotide at the position 1482 of human DGKK is a U not a C). Our study was focused on mouse *Snord116* and group I human *SNORD116*s, which share 100% sequence homology with each other at the predicted interacting site with *DGKK*. Consistent with the prediction, our results from human neuroblastoma SK-N-FI cells showed that U1483 of DGKK was 2′-O- methylated and that both *SNORD116* and *FBL* were involved in promoting the methylation of this site. Additionally, the compensatory mutation analysis described above (see Response to Major Point #1) showed that the predicted interaction (based on the sequence complementarity) between *Snord116* and *DGKK* was necessary to promote 2′-O-methylation of U1483. The RNA-DNA chimera probe (gapmer) to *DGKK* that we used in the Nm-VAQ assay was designed to detect 2′-O-methylation of U1483 specifically. We did not test if C1484 was also methylated. Based on the molecular principle of the Nm-VAQ method, we would not be able to distinguish if only U1483 or both U1483 and C1484 are methylated by using a new RNA-DNA chimera probe with the DNA sequence complementary to both U1483 and C1484 because methylation of either or both of the nucleotides would confer resistance to RNase H digestion in Nm-VAQ assays. Similarly, Ye et al did not detect 2′-O-methylation of U1483 in human ES-derived neurons. Based on the molecular principle of RioOxi-Seq method used in that study, Ye et al would not be able to distinguish whether only C1484 or both U1483 and C1484 were methylated in these cells.

Taken together, the results from our study and those of Ye et al suggest that both U1483 and/or C1484 of human *DGKK* may be modified by 2′-O-methylation. It might be possible to further examine these possibilities using additional methods and techniques, but we felt that extension would be beyond the scope of the current work and our expertise. We thank the reviewer for raising this important point. In the revised manuscript, we have cited the work by Ye et al and discussed the results from both our study and Ye et al and the possibility that human *SNORD116*s may promote 2′-O-methylation of these consecutive nucleotides of DGKK mRNA in human cells. This possibility would not alter our conclusions regarding the proposed physical interactions between *SNORD116*s and *DGKK*.

6) - RNase H digestion is often suboptimal in a Gapmer-dependent fashion, which can directly affect the quantification of methylation. To control for this, it is standard practice to use synthetic RNAs corresponding to the sequence of interest, carrying or lacking a 2′-O-methylation at the candidate site. Such controls would help determine whether the methylation percentages reported here reflect true biology or simply incomplete digestion efficiency. In the same line, it is surprising to control unmethylated sites at an unrelated RNA and not another (often close) position on the same RNA. These validation steps should be included.

**Response**: We thank the reviewer for raising this important point. We were aware of this potential problem and monitored RNase H activity in every Nm-VAQ assay by including the negative control G585 of human *GAPDH* mRNA (NM_001289745.2), which has been previously shown to be unmethylated (Tang et al, 2024, Ref 36). We found that the gapmer (chimera probe)-dependent digestion of GAPDH mRNA by RNase H was complete because very low background methylation signals for G585 on GAPDH mRNA were detected in every experiment (Supplemental Fig S7C). Despite the evidence of optimal digestion of the GAPDH-gapmer complex by RNase H, we acknowledge that there was evidence of suboptimal digestion of the DGKK-gapmer complex by RNase H in Nm-VAQ assay of 2′-O-methylation of U1483. The presence of residual methylation signals at U1483 of human DGKK in iPSCs with PWS type 1 deletion, which do not express any *SNORD116*, was consistent with suboptimal RNase H digestion of the DGKK-gapmer complex in these cells. Furthermore, the compensatory mutation analysis showed that there were ~10-25% residual methylation signals in HEK293 cells co-transfected with the combinations of *DGKK*-FLAG reporter construct and *Snord116*-expressing plasmid that were not expected to interact with DGKK. Because HEK293 cells do not express endogenous *DGKK* or *SNORD116*, these results again suggested that the residual methylation signals likely resulted from suboptimal or incomplete digestion of DGKK-gapmer complexes by RNase H. Despite the technical limitation imposed by the suboptimal digestion of DGKK-gapmer complexes by RNase H in Nm-VAQ assays, our results consistently demonstrated the presence of *SNORD116*-dependent 2′-O-methylation of U1483 of human *DGKK* in multiple cellular models by two different methylation detection assays. We address this point specifically in Results and Discussion in the revised manuscript (Pages 12-13, 18-19).

Multiple reports, including Ye et al, suggest that 2′-O-methylation of mRNAs occurs at much higher rates than had been previously expected and that most of these methylations may not be mediated by snoRNAs. Lack of 2′-O-methylation at G585 of human GAPDH mRNA (NM_001289745.2) has been demonstrated specifically by Tang et al (Ref 36) and was confirmed in our study (Supplemental Fig S7C). Because we did not know if any other nucleotides on *DGKK* were truly unmethylated, we considered that G585 of GAPDH mRNA was a reasonable control for monitoring RNase H activity in Nm-VAQ assays.

7) Rather than presenting the results in graphical form, the authors provide tables containing the individual Ct values of three replicates. The conclusions are based on mean Ct values, while standard deviations are not reported. In addition, PCR efficiency appears suboptimal: for example, in Fig. 7B, the use of 4 ng versus 18 ng of cDNA (a 4.5-fold input difference) yields Ct differences of 1.5 and 3.1 for Dgkk Ct1 and Ct2, corresponding to apparent ratios of 2.8 and 8.6, respectively. The PCR conditions should therefore be optimized to achieve robust amplification efficiency, and, most importantly, appropriate statistical analyses should be performed to support the authors' conclusions.

**Response**: We thank the reviewer for the comment. It is expected that qRT-PCR efficiency and reproducibility are reduced for lowly-expressed genes, resulting in high and variable Ct values (of technical replicas). In response to the reviewer's comment, we performed a full range PCR calibrations (Supplemental Fig S7A) and confirmed the key Nm-VAQ results using Taqman qPCR method, which in theory has lower nonspecific fluorescent background compared to SYBR Green-based qPCR assay. As shown in Supplemental Fig S7A, the SYBR Green-based qPCR assay exponentially amplified *DGKK* cDNA up to Ct value of 33 in the tested range from 25-36 cycle. We also repeated the qPCR part of some Nm-VAQ assays using higher RNA input (more RT mix), which resulted in tighter Ct values for technical replicas (Supplemental Fig S7B). Per the reviewer's suggestion, we performed statistical analysis on data obtained from at least three independent experiments. These results are now reported in Fig 6 of the revised manuscript. As the reviewer points out, previous publications usually present methylation levels in bar graphs. To be consistent with the convention, and to improve the readability, we have presented all methylation data in bar graphs in the revised Figure 7.

8) - The RTL-P data should be incorporated into the main figures of the manuscript, together with the appropriate controls, as these experiments are directly relevant to the main conclusions.

**Response:** We have now included the RTL-P data as a new subpanel in Fig 6 of the revised MS. The RTL-P data from iPSCs with PWS T1 deletion and the isogenic WT controls provide an independent validation of the results obtained using Nm-VAQ assay in the same cellular PWS model. In the WT iPSCs the percentage of Um1483 is ~ 30% by RTL-P assay and 42% by Nm-VAQ assay. However, when the percentages of Um1483 in WT iPSCs were arithmetically

reduced by the respective percentages of Um1483 in iPSCs with the PWS T1 deletion (the *Snord116* deficient control), the percentages of 2′-O-methylation of U1483 that were *SNORD116*-depdent were 20.9% by RTL-P and 17.2% by Nm-VAQ. As discussed above, the reasons for the presence of residual methylation signals in iPSCs with PWS T1 deletion were likely due to suboptimal digestion of DGKK-gapmer complexes by RNase H in the Nm-VAQ assay, and slightly lower RT efficiencies at the lower dNTP concentration in RTL-P assay. It is unlikely - but not impossible - that there was *SNORD116*-independent methylation of U1483 in human *DGKK*. Notwithstanding technical limitations in the detection of 2′-O-methylation, the results from both RTL-P and Nm-VAQ assays suggest that a significant fraction of 2′-O-methylation of U1483 of DGKK mRNA was *SNORD116*-dependent.

9) - RNA-seq data are classically validated by an orthogonal method (commonly RT-qPCR), at least for the genes that are used to support the main conclusions of the study. This would be particularly important for genes such as Snord64, Dhcr7, and the additional genes belonging to the pathways discussed by the authors. This represents a limited amount of additional work that will strengthen confidence in the dataset and in the corresponding interpretation.

**Response:** We performed additional qRT-PCR analyses on selected DEGs (please see Supplemental Figs S2-S3). We confirmed the differential expression status of *Snord64, Ank, and Dgkk* in P5 hypothalamus of *Snord116* KO and/or IC del models. By RNA sequencing, *Hsd17b7* was the most differentially expressed gene in the cholesterol biosynthesis pathway in both *Snord116* KO and IC del mice relative to their WT controls (log2FC=-0.15, FDR=0.9 in *Snord116* KO and log2FC=-0.14, FDR<0.01 in IC del). We performed qRT-PCR validation of *Hsd17B7*, which showed that *Hsd17B7* expression was indeed decreased (~15% lower) in P5 hypothalamus of *Snord116* KO mice relative to WT controls, but the difference did not reach statistical significance (p=0.25) (Supplemental Fig S2). The result was not unexpected given the relatively small changes in the transcript level of *Hsd17b7* and non-significant FDR in *Snord116* KO model. The log2FCs for *Dhcr7*, another gene in the cholesterol biosynthesis pathway, were even smaller than *Hsd17b7* (log2FC=-0.09, FDR=0.9 in *Snord116* KO and log2FC=-0.12, FDR=0.21 in *IC del*). No further validation of *Dhcr7* by qRT-PCR was carried out.

We agree with the reviewer that it would be ideal to confirm differential expression status of the genes belonging to significantly enriched pathways identified by GSEA. However, this approach may not be possible for some genes/pathways because changes in the transcript level of individual genes in the pathway are small. As reported in the original publication describing GSEA, the change in any individual gene in a pathway is often too small to be detected by conventional DEG analysis. GSAE was designed to detect the impact of genetic mutations/manipulations on a specific pathway by examining the changes of all genes in the pathway to increase statistical power to detect aggregate differences. (Mootha, et al, 2003,

PMID: 12808457; Subramanian et al, 2005, PMCID: PMC1239896).

10) Table 1: The relative expression values of *DGKK* across brain regions are not clearly defined. Does "++" indicate a two-fold increase compared to "+"?

**Response:** We thank the reviewer for raising this point. Our RNAscope experiments were not intended to be quantitative but only to show where *Dgkk* expression was detected in adult mouse brain and roughly the strength of the signals based on visual examination. We intended to show that the signal strength decreased in the order of ++++ > +++ > ++ > +. However, ++++ should not be taken as an indication that it was 2 times stronger than ++ or four times stronger than +. We have clarified this point in the legend of Table 1 in the revised manuscript.

11) Figure 5C: The quality of the Western blot (WB) is questionable; T1 del samples appear more loaded than wild-type (WT), and it seems as if number of lanes in ß-actin and in DGKK is different. "D" missing on figure.

**Response:** We agree with the reviewer that more T1 del samples were inadvertently loaded on the gel than the controls due to variations in protein concentrations of the cell lysates. We normalized the differences in the amounts of samples loaded by quantifying the volume of DGKK and beta-actin bands (both the size and intensity) and presented DGKK protein levels as the ratios of DGKK to beta-actin volumes above the background. Although the method is not fully quantitative, our approach is a standard practice for quantifying protein level by Western blotting.

There is a reasonable explanation for the apparent difference in the number of lanes in beta-actin and DGKK blots, which was due to increased size of beta-actin bands relative to DGKK bands on Western blot. DGKK is a very large protein (~170 kD) compared to b-actin (~45 kD). We used low concentration polyacrylamide gels (6%) in order to obtain a good separation of the DGKK band from the nonspecific band just below the DGKK band. The nonspecific bands were visible in both iPSCs and transfected or mock-transfected HEK293 cells, which served as positive and negative controls to identify the position of the true DGKK band on the gel. As a result, smaller protein bands, such as beta-actin, diffused to a greater extent, especially in the outer lanes, during electrophoresis and appeared as broader and thicker bands than those of large protein bands like DGKK. The full uncropped WB images are included in the Source Data for Fig 4 in the revised manuscript.

We intended to present the gel images and the quantification result as one plot (Fig 5C) in the original manuscript (Fig 4 in the revise MS). To make the figure easier to read, we have now separated the gel image and quantification date into two separate subpanels as Fig 4C and 4D in the revised MS, respectively.

12) Figure 8: The modified RNAscope technique used to assess Dgkk mRNA expression in the adult mouse brain lacks a specificity control (e.g., probe validation in expressing vs. non-expressing cells).

**Response:** We appreciate the reviewer's point. The specificity of the *Snord116* probe was validated in *Snord116* KO mice and WT controls (Supplemental Fig S8A in the revised MS). Because we did not have access to *DGKK* KO mouse tissue as control, we used several alternative approaches to evaluate/validate the specificity of the *DGKK* probe. 1. The algorithm used for RNAscope probe design ensured that the probe did not – by specific search- cross react with any known sequence in the mouse genome. 2. The *DGKK* staining pattern obtained using RNAscope was distinct from nonspecific staining. 3. qRT-PCR results on tissue samples collected from various brain regions of adult mice showed that *DGKK* expression was very low in the frontal cortex and cerebellum but high in the hypothalamus (Supplemental Fig S8B). These results were fully consistent with the RNAscope results. In contrast, *Snord116* was expressed at similar levels in all three brain regions examined by RNAscope and qRT-PCR.

13) Figure 9A-B: The nuclear localization of DGKK with SNORD116 is unclear due to insufficient image quality. The conclusion cannot be confidently drawn from the provided data.

**Response:** We apologize for the suboptimal image quality, which was likely compressed during the conversion to PDF. In the revised manuscript, we increased the size and resolution of the Z-stacking image to show more clearly the nuclear co-localization of *Snord116* and *DGKK*. The amount of *DGKK* mRNA co-localized with *Snord116* in the nucleus was relatively low, suggesting that the interaction between *Snord116* and *Dgkk* in the nucleus is likely transient. Although the biological significance of the co-localization of *DGKK* with *Snord116* in the nucleus remains to be fully defined, we have shown that a significant fraction of 2′-O-methylation of U1483 in *DGKK* mRNA is *SNORD116*-depdendent. The compensatory mutation analysis has further shown that the predicated interaction between *SNORD116* and *DGKK* is required for 2′-O-methylation of U1483 of DGKK mRNA. SNORDs have been previously implicated in the regulation of pre-RNA processing and 2'-O-methylation, both of which occur in the nucleus.

14) Figure 10: It is intriguing that cortical neurons were chosen to investigate DAG signaling, given that DGKK is minimally expressed in this brain region (or not at all, according to Figure 9). The authors should justify this choice or discuss potential influence of signaling circuits.

**Response:** Indeed, the cortex in adult mouse brain expresses very little *Dgkk*. The cortical neurons (MCNs) used for investigating mGluR1/5-DAG signaling were isolated from P0 pups and cultured *in vitro* for 14 days. We confirmed the differential expression status of *Dgkk* and *Snord116* in these neurons (Supplemental Fig S10 in the revised MS). Briefly, newly isolated

MCNs did not express significant amount of *DGKK*. As the neurons matured *in vitro*, *Dgkk* expression started to increase in both WT and *Snord116* KO neurons around DIV5 and continued to rise from DIV5 to DIV15, faster in *Snord116* KO neurons than WT controls (Fig S10B). We also determined *Dgkk* mRNA and *Snord116* expression in DIV14 MCNs used for mGluR1/5 signaling study, which showed that *DGKK* mRNA levels were significantly higher in *Snord116* KO neurons relative to WT controls (Fig S10D) and, as expected, *Snord116* was not expressed in KO MCNs (Supplemental FigS10 A and 10C). We were unable to verify DGKK protein expression in mouse neurons due to the lack of working antibody.

The relatively large tissue size and the reliability of methodology for isolating and culturing cortical neurons were the main reasons that we chose to use mouse cortical neurons for investigating mGluR1/5-DAG signaling. Isolating and culturing hypothalamic neurons in large numbers from P0 pups are technically challenging. In the revised MS, we included the *Dgkk* and *Snord116* expression data from MCNs in Supplemental FigS10 and modified the Results to provide clearer rationales for using cortical neurons in these studies (Page 15).

15) Figure 11: The effect of fasting on Dgkk expression should include details on data normalization.

**Response:** The *Dgkk* mRNA levels were quantified by qRT-PCR. RNA input of each sample was normalized using the geomean of two house-keeping genes, *Tbp* and *Ppia*, and expressed as relative levels in an arbitrary unit as described in Methods. *Dgkk* mRNA levels were calculated using delt-delta Ct method.

16) The statement "Expression of E2f target genes [...] was negatively enriched in Snord116-deficient mice, indicating a decreased proliferating cell population and/or precocious neuronal differentiation" is an overinterpretation. The pathway analysis only provides suggestive evidence, and no validation by other methods was performed. A more cautious tone is warranted.

**Response:** We have revised the specific statement to "Expression of E2f target genes [...] was negatively enriched in *Snord116*-deficient mice, suggesting that *Snord116* deficiency may decrease cell proliferation and/or accelerate neuronal differentiation". Interestingly, a recent report by Helwak et al (Helwak et al, 2024 PMCID: PMC11608373) has shown that *SNORD116*-deficient neurons derived from LUHMES human neural progenitor cells appear to undergo a slightly accelerated maturation process compared to WT neurons, supporting the notion that *Snord116* may influence neuronal differentiation process.

Other minor comments :
1) The scheme mentioned in the text (page 5) should be counted and referred to as a figure.

**Response**: Per the reviewer's suggestion, we have included the schematic diagram in the main text as Fig 1 in the revised MS. We indicate in the figure legend that the Figure is a rendering of data from previous studies.

2) Abbreviations should be listed in alphabetical order.

**Response**: Revised as suggested.

Graph representations should be standardized across figures.

**Response**: We converted some black and white bar graphs to colored ones with consistent color schemes representing *Snord116* deficient (KO/KD) in red and wild (WT) type/negative controls in blue.

3) Page 12: The meaning of MCH is unclear.

**Response**: MCH, melanin concentrating hormone, has been spelled out.

4) Page 14: Typo: Snoed116 → Snord116.

**Response**: Corrected. Thank you.

5) Page 15, line 13: Citation 33 does not seem relevant in this context.

**Response**: The citation in the original manuscript was for the IntaRNA program, which we used to predict RNA:RNA interactions. The interaction between *Snord116* and *Dgkk* predicted by IntaRNA was consistent with the results reported by Baldini et al (Baldini et al, 2022, PMCID: PMC8789076, Ref 13). In the revised manuscript, the paragraph was modified and these citations have been placed in more specific contexts.

**Reviewer #2 (Comments to the Authors (Required)):**

Zhang et al describe DGKK as a novel target for SNORD116

1) SNORD116 is an orphan SNORD that plays a key role in Prader-Willi syndrome. However, targets and mechanistical insights have been elusive. Thus identification of a new target as shown could be highly interesting and important.

The key finding is the sequence complementarity between SNORD116 and DGKK shown in

Figure 7A. Do the other genes shown in Figure 1A have SNORD116 binding sites? - or the genes that are similar between the ko models (Figure 1B)?

**Response:** We thank the reviewer for the comments. This point has also been raised by Reviewer 1. Please see our response to **Major point #9 by Reviewer 1** above. Again, we performed IntaRNA and qRT-PCR analyses on all shared DEGs shown in Fig 1B as part of the preliminary vetting process. These data are now included as Supplemental Table S6 and Figs S2-S3 in the revised manuscript. Briefly, several of the shared DEGs, such *Ankrd52, Ank, Hipk3, and Tmem231* were predicted to interact with mouse *Snord116* with similar strength as *Dgkk* (minimal free energy ≤ -13 kcal/mol). We confirmed the differential expression status of *Snord64, Ank,* and *Dgkk* in P5 hypothalamus of *Snord116* KO mice and that of *Dgkk* in 4- and 12-week-old *Snord116* KO mice (Fig 3A-C in the revised MS) and P5 IC del hypothalamus (Supplemental Fig S2 in the revised MS). However, none of the other shared DEGs were differentially expressed in 12-week-old hypothalamus of *Snord116* KO model. These results suggested that *Dgkk* was a strong candidate as a *Snord116* target in the mouse brain, the primary reason for focusing on this gene in the subsequent studies. However, we do not rule out the possibility that *Snord116* may (and probably does) interact with and influence the expression of other genes in the mouse brain. Additionally, three DEGs from *Snord116* KO vs WT (*Nkain1, Tll2, Dhx35*) (Supplemental Table S2), which were not shared by the IC del model (Supplemental Table S1), were also analyzed by IntaRNA. None of these genes were predicted to have as energetically favorable interactions with *Snord116* as *Dgkk* (minimal free energy > -10 kcal/mol) (data not shown).

In the revised manuscript, we include the preliminary vetting results, i.e., the IntaRNA and RT-qPCR data described above in Supplemental Table S6 and Figs S2 and S3 and modified the relevant part of Results to provide a clearer rationale for focusing on *Dgkk* in the subsequent studies (Pages 9-10)

2) The 2'-O-methylation should be recapitulated with reporter genes, where compensatory mutations and or RNA binding assays (RIP) could be employed. Currently, the data in fig 7 are not fully convincing (see below).

**Response:** We performed compensatory mutation analysis per reviewer's suggestion. The experiment have been described in detail in Results, Discussion, and Methods sections of the revised MS. Briefly, we obtained a DGKK-FLAG reporter construct (Gene Script) and the plasmid expressing mouse *Snord116* generated by Falaleeva et al (Falaleeva et al, PMCID: PMC5586535) and generated mutant DGKK-FLAG reporter construct and mutant *Snord116*-expressing plasmid in which the seeding sequence of the putative interacting sites of each RNA was mutated to the complementary sequence, i.e., from CCAAGGA to GGTTCCT in DGKK and from TCCTTGG to AGGACC in *Snord116*, respectively (Fig 6F in the revised manuscript). The mutant *Snord116* was not expected to interact with the WT *DGKK*, and vice

versa. However, the mutant *DGKK* and mutant *Snord116* sequences were fully complementary in the predicted interacting sites and were expected to interact with each other as the WT RNAs. Because HEK293 cells expressed no endogenous *DGKK* or *SNORD116*, we co-transfected HEK293 cells with the wild type or mutant DGKK-FLAG reporter construct in combination with the wild type or mutant *Snord116* plasmid and then determined 2'-O-methylation of U1483 on DGKK-FLAG reporter mRNA using Nm-VAQ method. As shown in Fig 6G of the revised manuscript, the percentage of Um1483 was 40.6% in cells co-transfected the wild type *DGKK* reporter and *Snord116*. In contrast, the percentage of 2'-O-methylation of U1483 was decreased to 27.0% in cells co-transfected with WT DGKK reporter and mutant *Snord116*. Restoration of the sequence complementarity restored 2'-O-methylation of U1483 to the wild type level (42.1%) in cells co-transfected with the mutant *DGKK* reporter and *Snord116* constructs.

The compensatory mutation analysis demonstrated that the predicted interaction between *DGKK* and *Snord116* was indeed required to promote 2'-O-methylation of U1483. The low level of methylation signals detected in cells co-transfected with the combinations of *DGKK* and *Snord116* constructs that lack sequence complementarity were likely due to incomplete digestion by RNase H specific to the *DGKK*-chimera probe complex in Nm-VAQ assay because digestion of the *GAPDH*-chimera probe complex, which was included as a control in all Nm-VAQ assay, was complete (Supplemental Fig7C in the revised MS). As Reviewer 1 has pointed out, incomplete or suboptimal RNase H digestion was often gene-chimera probe specific (Chen et al, 2006, PMID:16700890;Hagedorn et al 2017, PMC5389529). Incomplete digestion by RNase H may also contribute to the residual methylation signals observed in cells with either *SNORD116* KD (16.9%) or *FBL* knocked down (13.0%) or in iPSCs with PWS type 1 deletion (23.6%), which do not express *SNORD116* at all. In the revised MS, we have added the new results from the compensatory mutation study and discussed the residual methylation signals in *SNORD116* null cells likely resulting from the incomplete RNase H digestion of the DGKK-chimera probe complex in Results (Pages 12-13).

3) Figure 1A: all the genes and their fold changes should be presented in a table /Xls file. There have been several similar studies looking at SNORD116 knock outs (mostly human), and SNORD116 overexpression, which should be compared as well.

**Response:** Per the reviewer's suggestion, we have included all differentially expressed genes their fold changes from *Snord116* KO and IC del models as Supplemental Tables S1 and S2.

Multiple efforts have been made to identify *Snord116* targets in various model systems, including post-mortem hypothalamus from individuals with PWS or in PWS neuronal/cellular models of human origin (Burnett et al, 2017, PMCID: PMC5199710; Falaleeva et al, PMCID: PMC5586535; Bochukova et al, 2018, PMCID: PMC5896230; Gilmore et al, 2024, PMCID: PMC11662933; Helwak et al, 2024 PMCID: PMC11608373). The potential *SNORD116*-target gene lists identified in these studies showed little overlap with each other and

with the shared hypothalamic DEGs between IC del and *Snord116* KO mice identified in the current study. The lack of significant overlap among the potential *SNORD116* targets identified in these studies may have multiple causes, including variations in the model system, genetic background (between samples within a study and between different studies), and differentiation stage of neurons examined. We addressed this point in Discussion in the revised manuscript (Page 19). We noticed an interesting parallel in the decreases of expression of genes involved in the regulation of cell proliferation and cell cycle in the P5 mouse hypothalamus of *Snord116* KO and IC del mice and *SNORD116* deficient neurons derived from the LUHMES human neuronal precursor cells (Helwak et al, 2024, PMC11608373), which have shown that *SNORD116* deficiency appears to cause accelerated maturation of neurons compared to WT controls.

Our independent identification of *DGKK* as a potential target of *Snord116* in the mouse hypothalamus is consistent with earlier reports by Baldini et al and Ye et al. Baldini et al identified *DGKK* through phylogenetic analysis of the sequence complementarity between *SNORD116* and *DGKK* and validated the regulation of *DGKK* expression by ASO-mediated *SNORD116* knockdown in Hela S3 cells (Baldini et al, 2022, PMCID: PMC8789076). Ye et al reported that *DGKK* was one of the two genes that was differentially 2'-O-methylated in WT and *SNORD116*-deficient neurons derived from human stem cells (Ye et al, 2025, PMID: 41446073). Thus, evidence supporting *DGKK* as a potential *SNORD116* target gene has been observed across different species (mouse and human) and in different tissue/cellular models (hypothalamus, human neuroblastoma cells, HeLa S3 cells, and human neurons derived from stem cells). In all these studies, gene expression or methylation status were compared in *SNORD116* deficient models with the respective isogenic wild type controls, further strengthening the inference of interaction of these molecules (Baldini et al, 2022, PMCID: PMC8789076; Ye et al, 2025, PMID: 41446073; and the current study).

We have added these points to the Discussion in the revised manuscript (Pages 18-19).

4) Figure 5 C/D: the label D is missing in the figure. Figure 5C is essential for the paper, but of poor quality. There is a non-specific band under the DGKK bands in the negative control that disappears in the transfected positive control. The actin loading control is overexposed, making the quantification in D questionable. The complete gel including size makers should be shown.

**Response:** We intended to present the gel images and quantification result as one plot (Fig 5C, which is Fig 4 in the revised MS) in the original manuscript. To make the figure easier to read, we have separated the gel images and quantification date into two separate subpanels as Fig 4C and 4D in the revised MS.

The complete gel images of the DGKK and beta-actin Western blots including size markers, are shown in Source data in the revised manuscript. We acknowledge that the ability to detect the endogenous DGKK protein in iPSC cells was limited due to the low levels of DGKK protein in

these cells and by the low sensitivity and specificity of the antibody. We were careful in optimizing the electrophoresis conditions with both positive and negative controls, i.e., HEK293 cells transfected with or without FLAG-tagged human DGKK, to obtain clear separation of the true DGKK protein band from the slightly smaller non-specific band on SDS-PAGE. To confirm the accuracy of protein quantification by densitometry, we quantified DGKK protein levels using either light or dark exposures of beta-actin bands. The results of the quantification were very similar. These results are included in Source Data for Fig 4.

The reason for the absence of a visible non-specific band in the positive control was that we intentionally loaded less positive control lysate (PC1) (10 ul of lysate) relative to negative control lysate (NC, 30 ul) and PC2 (30 ul) so that the DGKK band in the positive control, which expressed the recombinant DGKK protein at much higher level than the endogenous DGKK protein in iPSCs, could be clearly separated from the slightly smaller non-specific band and would not be over exposed and bled into the adjacent lanes in Western blot.

We address this point by including the relevant information in the revised Fig 5C and 5D and the figure legend in the revised MS.

5) Figure 7 is the key mechanistical insight into the SNORD116-DGKK interaction. SNORD116 was knocked down with a gapmer. Why was not the crispr-based total (genetic) kd made in SHSY cells used? If SNORD116 causes the 2'O-methylation, it should go down to zero in the kd. In addition, ko mouse RNA could be used.

The most reliable way to measure 2'0-methylation is primer extension under limited dNTP concentration, which should be tried, as it gives less errors than the PCR based assays. Finally, a negative control, like 2'O-methylation of a rRNA should be used, which should also respond to the fibrillarin kd.

**Response:** In SHSY cells with crispr-based genetic knockdown, *SNORD116* expression was decreased by ~ 50% because a transposed *SNORD116* allele also expresses *SNORD116* in this line (Bochukova et al, 2018, PMC5896230, ref 17). Additionally, because *DGKK* expression was much lower in SHSY cells than in SK-N-FI cells (Supplemental Fig S5), the endogenous DGKK protein was not detectable in SHSY cells. The higher level of *DGKK* expression in SK-N-FI cells made the determinations of DGKK protein level and the detection of 2'-O-methylation possible in our experiences. Along the same line, *Dgkk* expression in mouse hypothalamus was also much lower (more than 10 -fold lower) than that of SK-N-FI cells based on their relative Ct values with similar total RNA input, making the detection of 2'-O-methylation of mouse *Dgkk* technically challenging (poorly reproducible data due to high *Dgkk* Ct values in Nm-VAQ assays). Despite the lack of methylation data from mouse hypothalamus, our results obtained from different PWS cellular models, including PWS T1 del (which is *SNORD116* null) and the isogenic WT iPSCs, using different detection methods (Nm-VAQ and RTL-P) as well as the

compensatory mutation analysis in HEK293 cells demonstrate that 2'-O-methylation of U1483 of human DGKK mRNA is *SNORD116*-dependent.

We thank the reviewer for asking why 2'-O-methylation of U1483 was not zero in the *SNORD116* or *FBL* knockdown cells. It might be expected that 2'-O-methylation of U1483 would not be completely abolished in *SNORD116* and *FBL* KD SK-N-FI cells because *SNORD116* or *FBL* expression were decreased but not completely abolished in these knockdown cells. However, we agree with the reviewer that in cells with complete absence of functional *SNORD116*, such iPSC cells with PWS T1 deletion and HEK293 cells co-transfected with the WT *DGKK* and mutant *Snord116*, zero or near zero methylation signals should be expected. The presence of clear methylation signals in these two distinct cellular models with complete deficiency of functional *SNORD116* suggests that the DGKK-chimera probe complexes may be partially resistant to RNase H digestion in Nm-VAQ assay. As the Reviewer 1 points out, suboptimal or incomplete RNase H digestion is often gene-ASO specific, which is also well documented in the literature (Chen et al, 2006, PMID:16700890;Hagedorn et al 2017, PMC5389529), because RNase H digestion was virtually complete for the negative control G585 of GAPDH mRNA in all Nm-VAQ assays that we have done (Supplemental Fig S7C). Independent RTL-P assay confirmed that there was *SNORD116*-dependent 2'-O-methylation of U1483 in the WT iPSCs. The levels of *SNORD116*-dependent 2'-O-methylation of U1483 as determined by RTL-P and Nm-VAQ was similar in the WT iPSCs, 20.9% vs.17.2%, respectively, when the methylation signals in the WT iPWS were subtracted for the residual methylation signals found in *SNORD116*-null T1 del cells. We have included these points in the Results section of the revised MS (Page 12).

We agree that primer extension is a more reliable method than PCR based method, especially for abundant RNA species like rRNA (Yu et al, 1997, PMCID: PMC1369484, Ref 37). However, it has been challenging to apply the primer extension method to genes with low expression levels, such as *DGKK*. One of the problems is that RT primers tend to have much higher nonspecific priming rates in the case of low abundant targeting RNA species. Methods with an additional PCR step, such as Nm-VAQ and RTL-P, enable the detection of methylation signals from low abundance RNAs without having to use radioactive isotopes; the nested PCR primers add another layer of specificity by amplifying only the correctly primed cDNAs in the reverse transcription step.

6) Figure 10 measures ERK and PKC signaling in response to SNORD116 kd, /DGKK increase. It is hard to make the connection to DGKK as there are at least 10 more diacetyl glycerol kinases in humans that could do the same reaction as DGKK.

**Response:** We thank the reviewer for raising this important point. RNAseq data showed that all known DGKs were expressed in P5 hypothalamus. *Snord116* KO or IC del appeared to affect the expression of *Dgkk* but not those of nine other known DGKs (Supplemental Fig S11 in the

revised MS). These data suggested that *Snord116* deficiency specifically affected *DGKK* expression in P5 hypothalamus. We also showed that *Dgkk* expression was significantly elevated in *Snord116* KO mouse cortical neurons (compared to WT controls) used for investigating ERK and PKC signaling (Supplemental Fig S10D in the revised MS). The inference from these results was that elevated *Dgkk* expression likely contributed to the attenuated mGluR1/5 - ERK/PKC signaling in *Snord116* KO MNCs.  We modified the relevant parts of Results and Discussion by including the RNAseq and qRT-PCR results mentioned above, and emphasized that an inference, not definitive conclusion, was drawn based on these data in the revised MS (Pages 15, 20).

Minor point: the paper has 12 figures and should be streamlined. A lot of the discussion about the sequences could go into the supplement.

**Response:** We thank the reviewer for the suggestion. All the analyses results derived from RNAseq, i.e., Figs 1-3 in the original manuscript, have now been combined into one figure with multiple panels (Fig 2 in the revised MS). Additional data derived from RNAseq and preliminary vetting of the shared DEGs have been included as supplemental materials, including differentially expressed genes (DEGs) and enriched pathways in the P5 hypothalamus of IC del and *Snord116* KO models, IntaRNA and qRT-PCR analysis results of the shared DEGs. Additional controls and validation results for Nm-VAQ and RNAscope assays, qRT-PCR analysis results of *Snord116* and *Dgkk* expression in primary mouse neurons used in mGluR1/5 - ERK/PKC signaling experiments are also included as Supplemental data in the revised manuscript.

**Reviewer #3 (Comments to the Authors (Required)):**

Zhang et al show convincing data for Snord116 regulates molecular pathways. Dgkk was isolated from other DEGs shared by both the mouse IC deletion and Snord116 KO. They show Snord116 and Dgkk expression are inversely correlated, and that Snord116/FBL complex may 2'-O methylate the Dgkk transcript. This involvement is unlikely to be exclusive, as there are additionally noncanonical functions of the Snords with potential overlapping functions. Downstream implications suggest dysfunctional nervous system development, impaired synapse maturation, and pathophysiological membrane signaling.
Major Points
1) There is still some debate in the field whether SNORD116 mutations alone are sufficient to recapitulate the full spectrum PWS phenotype. The phenotypic overlap with MAGEL2 truncating mutations, that cause SYS, supports the notion that PWS in most cases is caused by a combined loss of both SNORD116 and MAGEL2. That changes the impact of the 700 transcripts which were DE in the brain of the IC deletion mouse - which would include both SNORD116 and MAGEL2. There is, in fact, no discussion of MAGEL2 in the manuscript. The authors need

to address this discrepancy and perhaps take a closer look at genes regulated by the loss of BOTH SNORD116 and MAGEL2. Many more DEGs detected for the IC Del vs Snord116 mutant, I would like to see the differences addressed before focusing on the overlap only. What are the top DEGs in IC del? Where are the overlap DEGs found in the ranked list of DEGs for the IC del? Do they stratify near the top for logFC? Or are other genes/pathways with more change in the IC del group. I'd like to see the remaining 6 significant DEGs for Snord116 ko addressed, are those DEGs meaningful?

**Response:** We agree with the reviewer that *SNORD116* deficiency alone is unlikely sufficient to recapitulate the full spectrum PWS phenotype and that the majority PWS cases result from the loss of multiple paternally expressed genes in the region, including *SNORD116* and *MAGEL2*. Deficiency of *Magel2* causes Schaaf–Yang syndrome (SYS); the phenotypes are overlapping with *SNORD116* deficiency. IC del in mice abolishes the expression of all paternally expressed genes in this region and, not surprisingly, results in a substantially larger number of differentially expressed genes and pathways than the *Snord116* KO model (Supplemental Tables S1-S2 and S4-S5). As shown in Supplemental Table S1, the top differentially expressed genes in IC del hypothalamus were the maternally imprinted genes in the region, *including Snurf, Snrpn, Snhg14 (*the *Snord116* host gene*), Snord64, Magel2, Ndn, and Mkrn3*, validating the RNAseq results. These results also suggest that deficiencies of other paternally expressed genes in the IC del model, including *Magel2*, had significant effects on P5 hypothalamic transcriptome. *MAGEL2* is known to function as an enhancer for RING-type E3 ubiquitin-protein ligases by recruiting and stabilizing the E2 ubiquitin-conjugating enzyme at the E3:substrate complex. Ubiquitin-mediated protein degradation, a cellular protein turnover mechanism, plays an important role in regulating cell cycle and endosomal recycling. *Magel2* deficiency has been shown to disrupt the normal recycling of secretory granule proteins, leading to decreased numbers of secretory granules and decreased neuropeptide production (Chen et al 2020, PMCID: PMC7526459). Wijesuriya et al showed that *Magel2* controls leptin receptor sorting and degradation through a dynamic ubiquitin-dependent pathway (Wijesuriya et al 2017, PMCID: PMC5886282).  Although *Magel2* is not a transcription factor/regulator, it is conceivable that the loss of Magel2 would have direct/indirect effects on the hypothalamic transcriptome in mice.

The ranking of the shared DEGs among all DEGs identified in *Snord116* KO and *IC* del models have been included in Supplemental Table S6 in the revised manuscript. We found that the shared DEGs between *Snord116* KO and IC del models ranked relatively high among the 700 DEGs identified in IC del model. We also found that several Ubiquitin-mediated protein degradation pathways, such as APC_C_MEDIATED_DEGRADATION_OF_CELL_CYCLE_PROTEINS, and DEGRADATION_OF_BETA_CATENIN_BY_THE_DESTRUCTION_COMPLEX were negatively enriched in the IC del model but not in the *Snord116* KO model (Supplemental Tables S4 and S5). It is possible that *Magel2* deficiency in the IC del model may contribute to the changes in these pathways.

Only three DEGs, *Nkain1, Tll2, and Dhx35* in the *Snord116* KO model were located outside of the targeted region on Chr 7 and were not shared by the IC del model (Supplemental Table S2). The other three DEGs, *Snord107, Ndn and Gm4483*, were located in the targeted region on Chr 7. *Nkain1*, sodium/potassium transporting ATPase interacting 1 is an oncogene. *Tll2*, tolloid like 2, is an astacin-like zinc-dependent metalloprotease. and *Dhx35*, DEAH-box helicase 35, is predicted to be an RNA helicase. We performed IntaRNA analyses on *Nkain1, Tll2, and Dhx35.* No strong interactions between these genes and *Snord116*, similar to that of *DGKK*, were predicted by IntaRNA (minimal free energy >-10 kcal/mole) (data not shown). The biological significance of these DEGs in the pathogenesis of PWS is unclear.

2) Along these same lines, the authors do not justify why they did NOT take a closer look at the Fgd2 gene, which in Figure 1B is up regulated just as much as Dgkk. Why not look into this regulation?

**Response:** This point was also raised by both Reviewers 1 and 2. As in our responses to both above (**Major point #9 by Reviewer 1 and Major point #1 by Reviewer 2)**, we performed preliminary vetting of all the shared DEGs using IntaRNA and RT-qPCR. These data have been included as Supplemental Table S6 and Figs S2-S3 in the revised MS. The vetting results confirmed the differential expression status of *Dgkk, Snord64, and Ank* - but not *Fgd2* -in P5 hypothalamus. Additionally, no strong interaction between *Fgd2* and *Snord116* was predicted by IntaRNA (Supplemental Table S6). None of the shared DEGs, except *Dgkk*, were differentially expressed in 12-week-old *Snord116* KO hypothalamus (Supplemental Fig S3). Our results identified *Dgkk* as a potential *Snord116* target in the mouse hypothalamus and were consistent with the reports by Baldini et al (Baldini et al, 2022, PMCID: PMC8789076) and Ye et al (Ye et al, 2025, PMID: 41446073). Thus, *Dgkk* emerged as a strong and plausible candidate to be a *Snord116* target gene, the main rationale for focusing subsequent studies on *DGKK*.

In the revised manuscript, we include the preliminary vetting results, i.e., the IntaRNA and RT-qPCR data described above in Supplemental Table S6 and Figs S2 and S3 and modified the relevant part of Results to provide a clearer rationale for focusing on *Dgkk* in the subsequent studies (Pages 9-10)

3).If they think that loss of SNORD116 up regulates Dgkk in critical regions of the brain controlling feeding behavior and energy balance, why did they not look at either the IC del mouse or even the SNORD116 KO mouse brain for elevated Dgkk? Certainly they would want to show this by RNAscope!

**Response:** RNA sequencing and qRT-PCR analyses of mouse hypothalamus at different ages and several different human cellular models of PWS have consistently shown that *Snord116* deficiency led to elevated *Dgkk* expression. In the current study the main technical advantage of RNAscope vs RT-qPCR was to provide spatial information on the expression of *Dgkk* and

*Snord116*. We conducted preliminary studies of *Dgkk* expression in WT and *Snord116* KO mouse brains using RNAscope. Although most of *Dgkk*-expressing cells also expressed *Snord116*, there were substantial numbers of cells that appeared positive for only one or the other. We were not able to distinguish *Snord116*-expressing or non*Snord116*-expressing cell in *Snord116* KO mouse brain due to the lack of *Snord116* expression in all cells. Because of the technical limitations, RNAscope analysis was focused on determining the spatial distributions of *Dgkk* and *Snord116* expression in the WT mouse brain. We used qRT-PCR to compare expression levels of *DGKK* in *Snord116* KO and WT controls.

4) Given the focus on feeding behavior and metabolism, wouldn't the SNORD116 KO mice display hyperphagia? Apparently, they do not - according to the literature.

**Response:** We appreciate this vexing point. We showed that fasting led to decreased *Dgkk* expression but not *Dgkb*, one of the known DGKs, suggesting that the effect of fasting on *Dgkk* expression was specific. While these results implicate *Dgkk* in the regulation of energy metabolism in mice, the role of *DGKK* in the development of hyperphagia and obesity in PWS individuals remains unknown.

The following paragraph has been added in Discussion to address this point specifically. "The role of *DGKK* in the development of hyperphagic obesity associated with PWS is unknown. Mouse models of PWS, including the two used in the current study, do not develop hyperphagic obesity that is characteristic of PWS individuals (Refs 1-3, 19). The lack of hyperphagia and obesity phenotype in PWS mouse models may be due to divergence in the development of the feeding circuits (Herb, 2023, PMCID: PMC10631741), to runting in these mouse models (Refs 1-3, 19), and to differences in the genetic organization of the PWS regions in human and mouse (Refs 12-13) (Fig 1). The mouse *Snord116* cluster encodes virtually identical *Snord116* elements whereas human *SNORD116* cluster encodes *SNORD116* elements with diverse sequences (Fig 1), which may be expected to interact with molecular targets beyond those shared with mouse *Snord116* (Gilmore et al, 2024, PMC11662933). It is also of interest to note that human DGKK contains a unique segment composed of ~33 EPAP repeats (Glu48-Pro179) near the N-terminus, which is absent in mouse DGKK (34). The role of this domain is unknown, but it could potentially mediate interactions with proteins that are specific to human *DGKK* and may account for some of the phenotypic differences between PWS patients and mouse models of PWS."

Minor points

1) Fig 5c, the western blot shows a large nonspecific band that is also missing in WT. There is only one experimental replicate for this western blot and the western blot in Figure 10. The western in figure 10 contains two technical replicates(?) on a single gel and suggests a link with mGluR activity, however additional experimental replicates are needed to support this claim. The figure is labeled DIV14 but the caption shows DIV16, need to correct.

**Response:** This point has also been raised by Reviewer 2 **(Major Point #4)**. We performed additional Western blotting analysis for Fig 5C and the results were consistent. The large non-specific band missing (or less visible) in WT samples in Fig 5C was likely due to the lower amounts of WT lysate protein being loaded inadvertently because the protein concentrations were lower in the WT than the mutant lysates, which is consistent with the lighter actin bands in in in the WT samples than in the mutant samples. We quantified both DGKK and actin bands, and DGKK protein levels were expressed as the ratios of volumes of the DGKK to beta-actin bands to normalize differences in the amount of sample loaded.

The nonspecific bands were also much lighter in the positive control than in the negative control because we intentionally loaded only 1/3 amount of positive control (PC, 10 ul) lysate relative to negative control (NC, 30 ul) lysate to obtain better separation of the specific DGKK band and non-specific band on the Western blot.

We performed additional experiments shown in Fig 10 and the results were very similar in three additional experimental replicas. We have added this information in the revised Fig legend. Most of these experiments were done with two technical replicas for each experimental condition due to the limitation in the number of mouse neurons that could be isolated from sex and genotype-matched littermates in a single batch. The experiment shown in Fig 10 was done using DIV14 MCNs. We apologize for the typo and have corrected it in the revised manuscript.

2) Snord116 was misspelled at the beginning of the discussion.

**Response:** Corrected. Thank you.

October 6, 2026

RE: Life Science Alliance Manuscript #LSA-2026-03723-TR

Yiying Zhang

Dear Dr. Zhang,

Thank you for submitting your revised manuscript entitled "Diacylglycerol kinase kappa is a novel target of the Prader-Willi Syndrome gene SNORD116".

Your revised manuscript was reviewed by all the original reviewers, whose comments are appended below. As you will note, Reviewers 2 and 3 acknowledge that your revised manuscript addressed their concerns with some minor suggestions from Reviewer 2. Reviewer 1 notes that revised manuscript is improved whilst reiterating some concerns on mechanistic claims made and the need for careful interpretation of results. That said, we have noted your efforts in acknowledging some concerns (for example the indirect consequences of FBL depletion and technical caveats for assays used in this work) in your revised discussion.

In light of the overall positive evaluation from Reviewers 2 and 3, we would be happy to publish your paper in Life Science Alliance pending resolution of the points below, and final revisions necessary to meet our formatting guidelines.
-Whilst we do not need new experimental evidence to address Reviewer 1, you must explicitly acknowledge missing rescue experiments required to strengthen the conclusion on the effects of DgKK on downstream functional consequences.
-expand on the description of Figure 12 as suggested by Reviewer 2.

Please submit a manuscript in which the changes are highlighted and a point-by-point response to the reviewers' comments.

MANUSCRIPT ORGANIZATION AND FORMATTING:

To avoid unnecessary delays in the acceptance and publication of your paper, please read the following information carefully. Full guidelines are available on our Instructions for Authors page, https://www.life-science-alliance.org/authors

**Submission of a paper that does not conform to Life Science Alliance guidelines will delay the acceptance of your manuscript.**

-Please include a scale bar and size information for Figure S8 and include molecular mass information for blots in Figures 4,5, S4.
-You have the option of using Figure 12 as a Graphical Abstract in which case you would have to remove this figure file and upload it instead as a graphical abstract file, and edit the text accordingly.
-In line 577 you state, "despite the absence of evidence that FBL knockdown induced cellular stress/toxicity in SK-N-FI cells..." please either provide this evidence (absence of induced toxicity) or a citation or remove the claim.
-Please upload all figure files as individual files, including the supplementary figure files; all figure legends should only appear in the main manuscript file.
-Please add ORCID ID for corresponding (and secondary corresponding) author--you should have received instructions on how to do so.
-Please add the X and Bluesky handles of your host institute/organization, as well as your own, and/or one of the authors, in our system.
-The titles in both the system and the manuscript file must be consistent with each other.
-LSA does not permit citation of "data not shown," "manuscript in preparation," "manuscript submitted," etc., in any section of the manuscript. Please either remove the claim or include the evidence for statements in the following lines:238, 267, 509.
-Please remove the "Supplementary information" section on pg. 28.
-Please use the [10 author names et al.] format in your references (i.e., limit the author names to the first 10).
-Please add an Author Contributions section to your main manuscript text.
-Please add your main, supplementary figure, and table legends to the main manuscript text after the references section.
-There is a call-out for Figure 1C, please correct this since there is no panel 1C in the figure.
-Please add callouts for figures 1A-B; 2C; S2A-F; S3A-J; S8A-B; S10C and S11A-B to your main manuscript text.
-Please be sure that the authorship listing and order is correct.

We encourage our authors to provide original source data, particularly uncropped/-processed electrophoretic blots and spreadsheets for the main figures of the manuscript. If you would like to add source data, we would welcome one PDF/Excel-file per figure for this information. These files will be linked as supplementary "Source Data" files.

It is Life Science Alliance policy that if requested, original data images must be made available to the editors. Failure to provide original images upon request will result in unavoidable delays in publication. Please ensure that you have access to all original data images prior to final submission.

If you are planning a press release on your work, please inform us immediately to allow informing our production team and scheduling a release date.

We welcome submissions of potential cover images for the issue of LSA in which your work would appear. If you have high quality images associated with this work, please feel free to email these, with a caption, to the journal office.

LSA encourages authors to provide a 30-60 second video where the study is briefly explained. These videos will be appear embedded with the manuscript online at Life Science Alliance, and on social media to promote the published paper and authors (for examples, see https://docs.google.com/document/d/1-UWCfbE4pGcDdcgzcmiuJl2XMBJnxKYeqRvLLrLSo8s/edit?usp=sharing). Corresponding or first-authors are welcome to submit the video. Please submit only one video per manuscript. The video can be emailed to contact@life-science-alliance.org

FINAL FILES:

To upload the final version of your manuscript, please log in to your account: https://lsa.msubmit.net/cgi-bin/main.plex
You will be guided to complete the submission of your revised manuscript and to fill in all necessary information. Please get in touch in case you do not know or remember your login name.

The following items are required for acceptance.

-- An editable version of the final text (.DOC or .DOCX) is needed for copyediting (no PDFs).

-- High-resolution figure, supplementary figure and video files uploaded as individual files: See our detailed guidelines for preparing your production-ready images, https://www.life-science-alliance.org/authors

-- Summary blurb (enter in submission system): A short text summarizing in a single sentence the study (max. 200 characters including spaces). This text is used in conjunction with the titles of papers, hence should be informative and complementary to the title. It should describe the context and significance of the findings for a general readership; it should be written in the present tense and refer to the work in the third person. Author names should not be mentioned.

**The license to publish form must be signed before your manuscript can be sent to production. A link to the license to publish form will be available to the corresponding author only. Please take a moment to check your funder requirements.**

**Reviews, decision letters, and point-by-point responses associated with peer-review at Life Science Alliance will be published, alongside the manuscript. If you do want to opt out of having the reviewer reports and your point-by-point responses displayed, please let us know immediately.**

Thank you for your attention to these final processing requirements. Please revise and format the manuscript and upload materials as soon as you are able.

Thank you for this interesting contribution to the literature. We look forward to publishing your paper in Life Science Alliance.

Sincerely,

Sarita Hebbar, PhD
Scientific Editor
Life Science Alliance
http://www.lsajournal.org

------------------------------------------------------------------------------
Reviewer #1 (Comments to the Authors (Required)):

Zhang et al. have significantly improved their manuscript, but several major issues remain that need to be addressed in my opinion:

1. The evidence does not yet establish Dgkk as a direct SNORD116 target: the authors show reproducible DGKK dysregulation and use a predicted complementary SNORD116-Dgkk interaction, yet RNA colocalization and computational pairing do not demonstrate an endogenous RNA-RNA interaction. Most importantly, there is no perturbation of the endogenous predicted DGKK target site showing that this sequence is required for SNORD116-dependent regulation. The distinction between a direct

target and a downstream consequence therefore remains unresolved.

2. The evidence for SNORD116-directed 2′-O-methylation at DGKK is not correctly controlled: the RNase H-based assays exhibit substantial remaining signal making low-stoichiometry modification difficult to distinguish from signal background.
As asked in the first revision document, sequence-matched synthetic methylated and unmethylated DGKK standards are required to establish background (as in Tang et al. 2024, https://doi.org/10.1016/j.crmeth.2024.100721), dynamic range and sensitivity at a particular site as it is well known that cleavage efficiency is strongly sequence-dependent. Testing Gapdh or other sites is not a correct control of the experiment.
The fibrillarin-depletion experiment is also difficult to interpret mechanistically because fibrillarin depletion affects DGKK RNA abundance itself. Thus, the present data do not rigorously establish site-specific methylation or its dependence on SNORD116.

3. The mutational experiments do not provide endogenous mechanistic validation, as they are performed in an ectopic expression system and assess methylation rather than SNORD116-dependent regulation of endogenous DGKK. They therefore show that sequence complementarity can influence the methylation readout in an overexpression context, but do not establish that the predicted duplex is required for Dgkk regulation. A direct test would be to determine whether mutation of the predicted Dgkk site reduces or abolishes the response of Dgkk expression to SNORD116 depletion. Moreover, as methylation detection is not adequately controlled, the data are not sufficient to establish that the predicted interaction promotes site-specific 2′-O-methylation of Dgkk.

4. The connection between DGKK regulation and the downstream neuronal/PWS phenotype remains correlative. The manuscript links SNORD116 loss, DGKK dysregulation and altered mGluR-related phenotypes, but does not test whether normalizing DGKK reverses those phenotypes. Without such an epistasis/rescue experiment, DGKK can reasonably be presented as a molecular target candidate associated with the phenotype, but not yet as the mediator of the downstream functional consequences.

**Cross-review comments**
My only comment is that, despite the positive opinions expressed by the other two reviewers, I still feel the issues I raised in my review are of sufficient importance to warrant a major revision. Although the manuscript has improved relative to the initial version, central mechanistic issues remain unclear and therefore require more careful articulation.

Reviewer #2 (Comments to the Authors (Required)):

very convincing revision, including the compensatory mutations.
The last 'model figure' 12 could be improved by spelling out what is reported, observed, inferred, i.e. reported in this paper etc.

since this figure will be scanned by people outside the field, the gene names, pathways should be spelled out within the figure when possible. In the legend, the explanation of the model should be given as a brief overview.

Sup Figure S2, S3, "preliminary vetting", I have never seen this expression in a figure legend, could be omitted.

Reviewer #3 (Comments to the Authors (Required)):

The reviewers adequately addressed my concerns and especially the shared concerns among the reviewers. They make a strong case for Diacylglycerol kinase kappa as a direct target of the PWS associated gene SNORD116. I have no additional concerns.

## Response to Editor's comments and manuscript formatting requirements

-Whilst we do not need new experimental evidence to address Reviewer 1, you must explicitly acknowledge missing rescue experiments required to strengthen the conclusion on the effects of DGKK on downstream functional consequences.

**Response**: We have added the following paragraph in Discussion to address this point: "Effects of explicit normalization of *DGKK* expression in cellular and animal models on PKC and ERK activations downstream of mGluR1/5 and on specific PWS phenotypes are needed to confirm the role of *DGKK* in regulating mGluR1/5 signaling and pathophysiology of PWS." Please see Page 19 (highlighted).

-expand on the description of Figure 12 as suggested by Reviewer 2.

**Response**: This point is addressed in the revised manuscript. Please see Page 44 (highlighted)

Additionally, we have updated Data Availability by including the Accession number for RNA Sequencing data deposited in the NCBI Gene Expression Omnibus (GEO) database. Please see Page 27.

-Please include a scale bar and size information for Figure S8 and include molecular mass information for blots in Figures 4,5, S4.

**Response**: Scale bars and molecular mass information have been added.

-You have the option of using Figure 12 as a Graphical Abstract in which case you would have to remove this figure file and upload it instead as a graphical abstract file, and edit the text accordingly.

**Response**: Fig 12 will remain as a working hypothesis.

-In line 577 you state, "despite the absence of evidence that FBL knockdown induced cellular stress/toxicity in SK-N-FI cells..." please either provide this evidence (absence of induced toxicity) or a citation or remove the claim.

**Response**: We have added in Results the following statement and a Supplemental Table to address this issue: "Based on cell morphology and total RNA yields (Supplemental Table S7), no

apparent cellular toxicity associated with *FBL* KD was observed in SK-N-FI cells." Please see Page 12.

-Please upload all figure files as individual files, including the supplementary figure files; all figure legends should only appear in the main manuscript file.

**Response**: Done

-Please add ORCID ID for corresponding (and secondary corresponding) author--you should have received instructions on how to do so.

**Response**: done

-Please add the X and Bluesky handles of your host institute/organization, as well as your own, and/or one of the authors, in our system.

**Response**: Added

-The titles in both the system and the manuscript file must be consistent with each other.

**Response**: updated.

-LSA does not permit citation of "data not shown," "manuscript in preparation," "manuscript submitted," etc., in any section of the manuscript. Please either remove the claim or include the evidence for statements in the following lines:238, 267, 509.

**Response**: removed.

-Please remove the "Supplementary information" section on pg. 28.

**Response**: removed.

-Please use the [10 author names et al.] format in your references (i.e., limit the author names to the first 10).

**Response**: reformatted per the instruction.

-Please add an Author Contributions section to your main manuscript text.

**Response**: added. Please see Page 29.

-Please add your main, supplementary figure, and table legends to the main manuscript text after the references section.

**Response**: added

-There is a call-out for Figure 1C, please correct this since there is no panel 1C in the figure.

**Response**: corrected. It should be Fig 2C.

-Please add callouts for figures 1A-B; 2C; S2A-F; S3A-J; S8A-B; S10C and S11A-B to your main manuscript text.

**Response**: Callouts for all the above have been added to the main manuscript text.

-Please be sure that the authorship listing and order is correct.

**Response**: confirmed

-------------------------------------------------------------------------------

**Point-by-point response to Reviewers**

Reviewer #1 (Comments to the Authors (Required)):

Zhang et al. have significantly improved their manuscript, but several major issues remain that need to be addressed in my opinion:

1. The evidence does not yet establish Dgkk as a direct *SNORD116* target: the authors show reproducible *DGKK* dysregulation and use a predicted complementary *SNORD116*-Dgkk interaction, yet RNA colocalization and computational pairing do not demonstrate an

endogenous RNA-RNA interaction. Most importantly, there is no perturbation of the endogenous predicted *DGKK* target site showing that this sequence is required for *SNORD116*-dependent regulation. The distinction between a direct target and a downstream consequence therefore remains unresolved.

**Response**: We agree with the reviewer that we have not shown that perturbation of the predicted *DGKK* target site in the endogenous *DGKK* mRNA impacts *SNORD116*-dependent regulation of *DGKK* expression. Using compensatory mutation analysis we have shown that perturbations of the predicted *DGKK*/*SNORD116* interacting site in an overexpression system impacts the methylation of U1483 of human *DGKK*. However, even the existence of a direct interaction between *Snord116* and *DGKK* is not proof of functional consequence. We acknowledge, as we did in the discussion, that it remains unclear whether the predicted interaction based on the sequence complementation between *SNORD116* and *DGKK* and/or the methylation of U1483 are necessarily the underlying mechanisms for the increased *DGKK* expression associated with *Snord116* deficiency. Language emphasizing this point has been added in Discussion on Page 17.

2. The evidence for *SNORD116*-directed 2'-O-methylation at *DGKK* is not correctly controlled: the RNase H-based assays exhibit substantial remaining signal making low-stoichiometry modification difficult to distinguish from signal background.
As asked in the first revision document, sequence-matched synthetic methylated and unmethylated *DGKK* standards are required to establish background (as in Tang et al. 2024, https://doi.org/10.1016/j.crmeth.2024.100721), dynamic range and sensitivity at a particular site as it is well known that cleavage efficiency is strongly sequence-dependent. Testing Gapdh or other sites is not a correct control of the experiment.
The fibrillarin-depletion experiment is also difficult to interpret mechanistically because fibrillarin depletion affects *DGKK* RNA abundance itself. Thus, the present data do not rigorously establish site-specific methylation or its dependence on *SNORD116*.

**Response**: We acknowledge that there are significant background methylation signals at U1483 of human *DGKK* from multiple cellular systems, which may result from incomplete RNase H digestion and/or *SNORD116*-independent modification of the nucleotides in the vicinity. Despite the limitations of the methodologies we used in our study, the evidence obtained using different detection methods in multiple cellular systems, including ASO-mediated *SNORD116* knockdown, iPSC cells with PWS Type 1 deletion, and compensatory mutation analysis in HEK293 cells, supports the inference that there was *Snord116*-dependent 2'-O-methylation of U1483 in human *DGKK* mRNA in the cellular systems examined and that sequence complementation between *Snord116* and human *DGKK* was required for *Snord116*-dependent 2'-O-methylation of U1483 in *DGKK* mRNA in an overexpression model.

3. The mutational experiments do not provide endogenous mechanistic validation, as they are performed in an ectopic expression system and assess methylation rather than *SNORD116*-dependent regulation of endogenous *DGKK*. They therefore show that sequence complementarity can influence the methylation readout in an overexpression context, but do not establish that the predicted duplex is required for Dgkk regulation. A direct test would be to determine whether mutation of the predicted Dgkk site reduces or abolishes the response of Dgkk expression to *SNORD116* depletion. Moreover, as methylation detection is not adequately controlled, the data are not sufficient to establish that the predicted interaction promotes site-specific 2'-O-methylation of Dgkk.

**Response**: We acknowledge that compensatory mutation analysis does not provide endogenous mechanistic validation although the overexpression system is widely used to support results obtained in physiological conditions and in non-overexpressed cellular models.  In our study, compensatory mutation analysis is only one of several lines of evidence suggesting that there was *SNORD116*-dependent 2'-O-methylation of U1483 in human *DGKK* mRNA in the cellular systems that we examined. The conclusion from our study is that *SNORD116* interreacts with *DGKK* through the observed sequence complementation and promotes its methylation. However, as mentioned in the response to Point #1, it remains unclear whether the sequence complementation between *SNORD116* and *DGKK* is required for the elevated *DGKK* expression observed in *SNORD116* deficient cells, which is one of the limitations of the current study. We have further emphasized these reservations in the revised manuscript (Page 17).

4. The connection between *DGKK* regulation and the downstream neuronal/PWS phenotype remains correlative. The manuscript links *SNORD116* loss, *DGKK* dysregulation and altered mGluR-related phenotypes, but does not test whether normalizing *DGKK* reverses those phenotypes. Without such an epistasis/rescue experiment, *DGKK* can reasonably be presented as a molecular target candidate associated with the phenotype, but not yet as the mediator of the downstream functional consequences.

**Response**: We agree with the reviewer that the connection between elevated *DGKK* expression and the downstream neuronal/PWS phenotype is correlative and that rescue experiments by normalizing *DGKK* expression are required to draw a firm conclusion. To address this important point, we have added the following paragraph in Discussion to explicitly acknowledge the limitation of the current study: "Effects of explicit normalization of *DGKK* expression in cellular and animal models on PKC and ERK activations downstream of mGluR1/5 and on specific PWS phenotypes are needed to confirm the role of *DGKK* in regulating mGluR1/5 signaling and pathophysiology of PWS."  Please see Page 19 in the revised manuscript.

**Cross-review comments**
My only comment is that, despite the positive opinions expressed by the other two reviewers, I still feel the issues I raised in my review are of sufficient importance to warrant a major revision. Although the manuscript has improved relative to the initial version, central mechanistic issues remain unclear and therefore require more careful articulation.

**Response**: We acknowledge that additional experiments, such as normalization of *DGKK* expression, are needed for further strengthen the role of *DGKK* in mGluR1/5 signaling and PWS pathogenesis, as well as the possible existence of additional mechanism through which *SNORD116* regulates *DGKK* expression. In efforts to date, we have shown that *DGKK* expression is regulated by *SNORD116* in multiple cellular and animal models. We have also demonstrated *SNORD116*-dependent 2'-O-methylation of U1483 in human *DGKK* mRNA and shown that the observed sequence complementation between *Snord116* and *DGKK* is required for *Snord116*-dependent 2'-O-methylation of U1483. We have also shown that PKC and ERK activations downstream of the group1 metabotropic glutamate receptors (mGluR1/5), which are known to be activated by plasma membrane diacylglycerol, are attenuated or diminished in *Snord116*-deficient mouse neurons. This observation is consistent with the elevated *DGKK* expression in *Snord116*-deficient neurons. We have endeavored to interpret these results in a conservative fashion, and to acknowledge explicitly the limitations of the current study. We agree that much of the argument is inferential but consider it very useful for the design of further mechanistic studies of SNORD biology.

Reviewer #2 (Comments to the Authors (Required)):

very convincing revision, including the compensatory mutations.
The last 'model figure' 12 could be improved by spelling out what is reported, observed, inferred, i.e. reported in this paper etc.

since this figure will be scanned by people outside the field, the gene names, pathways should be spelled out within the figure when possible. In the legend, the explanation of the model should be given as a brief overview.

**Response**: We thank the reviewer for the suggestion. A detailed description of the model, such as gene names and pathways, as well as what is previously reported and what is observed or inferred in the current study, is now provided in the legend. Please see Page 44.

Sup Figure S2, S3, "preliminary vetting", I have never seen this expression in a figure legend, could be omitted.

**Response**: The phase is removed from both figures.

Reviewer #3 (Comments to the Authors (Required)):

The reviewers adequately addressed my concerns and especially the shared concerns among the reviewers. They make a strong case for Diacylglycerol kinase kappa as a direct target of the PWS associated gene *SNORD116*. I have no additional concerns.

**Response**: We thank the reviewer for the endorsement.

Finally, we thank all three reviewers and the editor for their time, generosity, and expert comments and suggestions, which have helped us to improve the manuscript.

October 20, 2026

RE: Life Science Alliance Manuscript #LSA-2026-03723-TRR

Dr. Yiying Zhang
Columbia University Irving Medical Center
Pediatrics
1150 St. Nicholas Ave
New York, NY 10032

Dear Dr. Zhang,

Thank you for responding to our formatting requests and submitting your Research Article entitled "Diacylglycerol kinase kappa is a target of the Prader-Willi Syndrome gene SNORD116".

It is a pleasure to let you know that your manuscript is now accepted for publication in Life Science Alliance. Congratulations on this interesting work.

The final published version of your manuscript will be deposited by us to PubMed Central upon publication.

Your manuscript will now progress through copyediting and proofing. It is journal policy that authors provide original data upon request.

Your article will publish open access upon publication under a CC-BY license.

Reviews, decision letters, and point-by-point responses associated with peer-review at Life Science Alliance will be published, alongside the manuscript. If you do want to opt out of having the reviewer reports and your point-by-point responses displayed, please let us know immediately.

***IMPORTANT: If you will be unreachable at any time, please provide us with the email address of an alternate author. Failure to respond to routine queries may lead to unavoidable delays in publication.***

Scheduling details will be available from our production department. You will receive proofs shortly before the publication date. Only essential corrections can be made at the proof stage so if there are any minor final changes you wish to make to the manuscript, please let the journal office know now.

DISTRIBUTION OF MATERIALS:
Authors are required to distribute freely any materials used in experiments published in Life Science Alliance. Authors are encouraged to deposit materials used in their studies to the appropriate repositories for distribution to researchers.

You can contact the journal office with any questions, contact@life-science-alliance.org

Again, congratulations on a very nice paper. I hope you found the review process to be constructive and are pleased with how the manuscript was handled editorially. We look forward to future exciting submissions from your lab.

Sincerely,

Sarita Hebbar, PhD
Scientific Editor
Life Science Alliance
http://www.lsajournal.org
