# Supplementary material for: Diacylglycerol kinase kappa is a target of the Prader–Willi syndrome gene SNORD116
Source: Life Sci Alliance. 2026 Nov 4;10(1):e202603723. doi: 10.26508/lsa.202603723 (PMC13736183; doi:10.26508/lsa.202603723)
Supplement: Supplementary file 2 [file LSA-2026-03723_TableS10.docx]

**Supplemental Table S10. Sequences of primers (5'-3') used in 2'-O-methylation assays and mutagenesis.**

| **RTL-P assay** | | |
| --- | --- | --- |
| *hDGKK* | Unanchored RT primer  (hDGKK-MeUA-RT) | GCTTCTGAAGCAACATTCCTTG |
|  | Anchored RT primer  (hDGKK-MeA-RT) | CTTCTGAAGCAACATTCCTTGGAA |
|  | PCR primer, forward | AGTGTGTCATGAGAGCTGTGG |
|  | PCR primer, reverse | GAAGCAACATTCCTTGGAAAACC |
| **Nm-VAQ assay** | | |
| *hGAPDH* | Chimera probe | dCdTdTdCUmCmAmUmGmGmUmUmCmAmCmAmCm |
|  | RT primer | GGTCATGAGTCCTTCCACGA |
|  | PCR primer, forward | TTCGTCATGGGTGTGAACCA |
|  | PCR primer, reverse | GGTCATGAGTCCTTCCACGA |
| *hDGKK* | Chimera probe | dAdAdAdACmCmGmUmCmUmCmCmUmAmCmAmGm |
|  | RT primer | CTTCTGAAGCAACATTCCTTGGAA |
|  | PCR primer, forward | AGTGTGTCATGAGAGCTGTGG |
|  | PCR primer, reverse | GAAGCAACATTCCTTGGAAAACC |
| h*DGKK*_mu_ | RT primer | ATCGCCTTTGGGGTCGCTTA |
|  | PCR primer, forward | TCCGCTGCCTGTGGTGTAAT |
|  | PCR primer, reverse | TGGGGTCGCTTAGAGCAGTG |
| **Primers for compensatory mutagenesis** | | |
|  | **forward primer** | **reverse primer** |
| h*DGKK* | TCCTATGTTGCTTCAGAAGC | ACCAAAACCGTCTCCTACAG |
| *Snord116* | AAAAGCTGAACAAAATGAG | ATGTTTGACTGGGAATC |
