# Supplementary material for: Diacylglycerol kinase kappa is a target of the Prader–Willi syndrome gene SNORD116
Source: Life Sci Alliance. 2026 Nov 4;10(1):e202603723. doi: 10.26508/lsa.202603723 (PMC13736183; doi:10.26508/lsa.202603723)
Supplement: Supplementary file 3 [file LSA-2026-03723_TableS3.docx]

**Supplemental Table S3. T statistics (t values) of the shared Hallmark_E2F_Target Leading Edge genes in the P5 hypothalamus of IC del and *Snord116* KO mice**

| **Overlapping E2F target genes** | **Snord116 KO (n=3M & 2F)** | **IC del male**  **(n=5)** | **IC del female**  **(n=5)** |
| --- | --- | --- | --- |
| *Espl1* | -0.803700135 | -2.924288127 | -1.380604834 |
| *Birc5* | -1.461812917 | -3.993585794 | -3.74631447 |
| *Ccnb2* | -0.918971244 | -1.935056365 | -3.732377604 |
| *Cdkn2c* | -0.805824714 | -4.528415128 | -3.404601415 |
| *Hmmr* | -1.167266609 | -3.386164554 | -2.259430062 |
| *Kif4* | -1.062954998 | -2.051817345 | -2.212220206 |
| *Kpna2* | -1.917569829 | -1.885487582 | -3.427426947 |
| *Lig1* | -1.040800982 | -2.078185665 | -3.811745291 |
| *Mki67* | -0.897599101 | -3.324895439 | -1.798349753 |
| *Rrm2* | -0.923200868 | -4.92692636 | -1.85317845 |
| *Top2a* | -0.925703444 | -4.080585756 | -2.839977523 |
| *Cenpe* | -1.233065912 | -3.035041154 | -2.675710478 |
| *Racgap1* | -1.18880768 | -3.079542869 | -4.572579005 |
| *Cks2* | -1.057545453 | -2.207091486 | -2.172699042 |
| *Spc25* | -1.399034437 | -3.98633537 | -1.473254582 |
| *Asf1b* | -0.923786109 | -2.389113053 | -1.864093288 |
| *Ube2t* | -1.764937471 | -2.515193735 | -2.036330565 |
| *Spc24* | -1.400916924 | -1.854667485 | -2.34202611 |
| *Smc4* | -0.761103924 | -3.047253629 | -2.964324505 |
| *Atad2* | -1.189837618 | -4.18570403 | -2.537137853 |
