# Supplementary material for: Diacylglycerol kinase kappa is a target of the Prader–Willi syndrome gene SNORD116
Source: Life Sci Alliance. 2026 Nov 4;10(1):e202603723. doi: 10.26508/lsa.202603723 (PMC13736183; doi:10.26508/lsa.202603723)
Supplement: Supplementary file 4 [file LSA-2026-03723_TableS7.docx]

**Supplemental Table S7: Total RNA yield (ng) from *FBL* knockdown (KD) and negative control (NC) SK-N-FI cells with 4 experimental replicas in a typical *FBL* KD experiment.**

| **NC** | **FBL KD** |
| --- | --- |
| 4403 | 4544 |
| 4242 | 4943 |
| 5105 | 4269 |
| 4954 | 4902 |
