# Supplementary material for: Diacylglycerol kinase kappa is a target of the Prader–Willi syndrome gene SNORD116
Source: Life Sci Alliance. 2026 Nov 4;10(1):e202603723. doi: 10.26508/lsa.202603723 (PMC13736183; doi:10.26508/lsa.202603723)
Supplement: Supplementary file 5 [file LSA-2026-03723_TableS8.docx]

**Supplemental Table S8. qRT-PCR primers used for gene expression analyses.**

| **Gene name** | **Forward primer (5'-3')** | **Reverse primer (5'-3')** |
| --- | --- | --- |
| ***Human genes*** |  |  |
| *SNORD116* | cgatgatgagtcccccataaaaac | cagttccgatgagaacgacg |
| *DGKK* | AGTGTGTCATGAGAGCTGTGG | GAAGCAACATTCCTTGGAAAACC |
| *SNRPN* | gctgcagcacattgactatagaat | cacagtcatggataccaagttctc |
| *MAP2* | GGGTCACAGGGCACCTATTC | CAGGACTGCTACAGCCTCAG |
| *TUBB3* | GGGAGATCGTGCACATCCAG | TAGACGCTGATCCGCTCCAG |
| *PPIA* | CCCACCGTGTTCTTCGACAT | CAAATCCTTTCTCTCCAGTGCTC |
| *TBP* | TGCACAGGAGCCAAGAGTGAA | CACATCACAGCTCCCCACCA |
| ***Mouse genes*** |  |  |
| *Snord116* | ATTCCCAGTCAAACATTCCTTGG | CAGTTCCGATGAGAGTGGCG |
| *Dgkk* | TGTTCCTTCGGAAGTCGTCG | TGAGCAAGGGACAGGAACAA |
| *Dgkb* | TGCCTCCATTGCACACAGAT | CTTCTTGCAGGTGGCTGAGA |
| *Ppia* | CCCACCGTGTTCTTCGACAT | CAAATCCTTTCTCTCCAGTGCTC |
| *Tbp* | CAGCCTTCCACCTTATGCTCA | CTGTGGAGTAAGTCCTGTGCC |
