# Supplementary material for: Diacylglycerol kinase kappa is a target of the Prader–Willi syndrome gene SNORD116
Source: Life Sci Alliance. 2026 Nov 4;10(1):e202603723. doi: 10.26508/lsa.202603723 (PMC13736183; doi:10.26508/lsa.202603723)
Supplement: Supplementary file 6 [file LSA-2026-03723_TableS9.docx]

**Supplemental Table S9. Primers used for genotyping and sex determination.**

| **Gene name** | **Primer sequence (5'-3')** |
| --- | --- |
| ***Snord116 WT and del alleles*** |  |
| PA (Snord116flox R7960) | TTTACGGTACATGACAGCACTCAAG |
| PC (Snord116flox R7958) | AATCCCCAACCTACTTCAAACAGTC |
| PD 9 (Snord116flox R7959) | TGGATCTCTCCTTGCTTGTTTTCTC |
| ***ICdel WT and del alleles*** |  |
| PWSIC-9973 | CCGCATTTCATCATTCTCAGGCTC |
| PWSIC-9974 | GTCACGTCCTGCACGACGCGAG |
| PWSIC-9976 | GGCATTGCTCGTGTGCCTCTTGC |
| ***mouse Sry for sex determination*** |  |
| mSry-F244 | GTGTCACAGAGGAGTGGCAT |
| mSry-R375 | GGAGTACAGGTGTGCAGCTC |
| mSry-probe | TGCAGTGGGACAGGAACCCACATGCC (5' 6-FAM/ZEN/3' IB) |
| ***mouse Chr6*** |  |
| mChr6-F | TTCCCTCGTGATCTGCAACT |
| mChr6-R | CACCTGTTCAATTCCC-CTGC |
| mChr6 probe | TCTTCTGGGCAGGCTTAAAGGCTAACC T- (5' HEX/ZEN/3' IBFQ) |
